# Identification of a novel BET bromodomain inhibitor-sensitive, gene regulatory circuit that controls Rituximab response and tumour growth in aggressive lymphoid cancers

**DOI:** 10.1002/emmm.201202034

**Published:** 2013-07-04

**Authors:** Anouk Emadali, Sophie Rousseaux, Juliana Bruder-Costa, Claire Rome, Samuel Duley, Sieme Hamaidia, Patricia Betton, Alexandra Debernardi, Dominique Leroux, Benoit Bernay, Sylvie Kieffer-Jaquinod, Florence Combes, Elena Ferri, Charles E McKenna, Carlo Petosa, Christophe Bruley, Jérôme Garin, Myriam Ferro, Rémy Gressin, Mary B Callanan, Saadi Khochbin

**Affiliations:** 1 CEAiRTSV, Biologie à Grande EchelleGrenoble, France; 2 INSERM, U1038Grenoble, France; 3 Joseph Fourier UniversityGrenoble, France; 4 INSERM, U823, Institut Albert BonniotGrenoble, France; 5 Pôle recherche, CHU-GrenobleGrenoble, France; 6 Onco-Hematology Genetics Unit, Plateforme hospitalière de génétique moléculaire des tumeurs, Pôle de biologieCHU-Grenoble, Grenoble, France; 7 Department of Chemistry, University of Southern CaliforniaLos Angeles, California, USA; 8 Institut de Biologie Structurale Jean-Pierre Ebel, UMR 5075 CEA-CNRS-Université de GrenobleGrenoble, France; 9 Department of Clinical Hematology, CHU-GrenobleGrenoble, France

**Keywords:** cancer-testis factor, CCDC86, CD40, double-hit B-cell non-Hodgkin's lymphoma, R-CHOP

## Abstract

Immuno-chemotherapy elicit high response rates in B-cell non-Hodgkin lymphoma but heterogeneity in response duration is observed, with some patients achieving cure and others showing refractory disease or relapse. Using a transcriptome-powered targeted proteomics screen, we discovered a gene regulatory circuit involving the nuclear factor CYCLON which characterizes aggressive disease and resistance to the anti-CD20 monoclonal antibody, Rituximab, in high-risk B-cell lymphoma. CYCLON knockdown was found to inhibit the aggressivity of MYC-overexpressing tumours in mice and to modulate gene expression programs of biological relevance to lymphoma. Furthermore, CYCLON knockdown increased the sensitivity of human lymphoma B cells to Rituximab *in vitro* and *in vivo*. Strikingly, this effect could be mimicked by *in vitro* treatment of lymphoma B cells with a small molecule inhibitor for BET bromodomain proteins (JQ1). In summary, this work has identified CYCLON as a new MYC cooperating factor that autonomously drives aggressive tumour growth and Rituximab resistance in lymphoma. This resistance mechanism is amenable to next-generation epigenetic therapy by BET bromodomain inhibition, thereby providing a new combination therapy rationale for high-risk lymphoma.

The nuclear factor CYCLON is a new MYC cooperating factor that drives tumor growth and Rituximab resistance in lymphoma. This resistance mechanism can be targeted by next-generation epigenetic therapy by BET bromodomain inhibition downstream of MYC.

## INTRODUCTION

B-cell non-Hodgkin lymphoma (B-NHL) is a clinically heterogeneous disease that is currently classified into multiple disease subtypes defined by discrete clinical, morphological, immunological and genetic features (Swerdlow et al, 2008). Molecular profiling studies in diffuse large B-cell lymphoma (DLBCL), the most common form of B-NHL, have revealed the existence of three molecular subtypes, the germinal centre B-cell-like (GCB-DLBCL), the activated B-cell-like (ABC-DLBCL) and primary mediastinal large B-cell lymphoma (Alizadeh et al, 2000; Pasqualucci, 2013). ABC-DLBCL consistently shows inferior survival compared to the GCB-DLBCL particularly under the current, standard-of-care, immuno-chemotherapy regimen, R-CHOP [Rituximab, Cyclophosphamide, Doxorubicin Hydrochloride, Vincristine Sulphate (Oncovin), Prednisone] (Lenz et al, 2008). However, treatment failure is also observed in GCB-DLBCL (20% within the first 2 years, based on progression free survival) (Lenz et al, 2008). Although MYC gene alterations are implicated in this setting and are associated with poor outcome (Barrans et al, 2010; Cuccuini et al, 2012; Johnson et al, 2009; Savage et al, 2009; Visco et al, 2013), the specific effectors of MYC dependency in DLBCL are not well characterized (Aukema et al, 2011). Aggressive lymphoma with features intermediate between DLBCL and Burkitt lymphoma (BL) are also described (Swerdlow et al, 2008). In a significant subset of cases, these are characterized by concurrent *MYC* and *BCL2* or *BCL6* gene translocations (Aukema et al, 2011; Lindsley & LaCasce, 2012). Lymphoma presenting this cytogenetic profile are commonly referred to as ‘double hit’ lymphoma. Despite their poor prognosis, evidence-based, risk-adapted treatment strategies and clinical, pathologic and genetic factors that predict response to therapy are lacking in these cases (Aukema et al, 2011; Lindsley & LaCasce, 2012).

We have postulated that a universal consequence of genetic and epigenetic perturbations in cancer cells, including lymphoma, is the illegitimate activation of cell type or differentiation stage-specific genes and proteins (Rousseaux & Khochbin, 2009; Wang et al, 2011), a process that we refer to as ‘off-context’ gene activation. We further propose that this ‘off-context’ gene expression provokes a cancer cell ‘identity crisis’ that permits evasion from normal cellular controls, ultimately leading to disease progression and treatment resistance (Rousseaux & Khochbin, 2009; Wang et al, 2011). This concept is best exemplified by the case of aberrant expression of testis specific/germ cell factors in somatic cancer cells (Rousseaux et al, 2013). Important in this respect is the fact that a significant proportion of testis specific genes encode potent epigenetic writers or readers that control reprogramming of the meiotic and post-meiotic haploid genome from a ‘somatic type’ organization to a gamete specific organization, involving almost genome wide replacement of histones and successive assembly of transition proteins and protamines (Rousseaux & Khochbin, 2009). Aberrant expression of testis specific factors, including epigenetic regulators, is increasingly described in diverse cancer types (Wang et al, 2011). This appears to drive abnormal epigenome reprogramming leading to malignant transformation and the emergence of cancer cell clones. As an example, expression of the male germ cell factor, DNMT3L, a catalytically inactive member of DNA methyl transferases, has been described in a variety of cancers. Through its ability to increase the activity of the *de novo* methyl transferases, it could contribute to CpG island methylation and pathological gene silencing (Gokul et al, 2009). Likewise, expression of NUT (nuclear protein in testis), encoded by a testis specific gene, is abnormally activated through fusion with the ubiquitously expressed BET bromodomain protein-encoding gene, *BRD4*, in an aggressive subset of carcinoma known as NUT-Midline Carcinoma (French, 2012). The resultant BRD4-NUT fusion gene encodes a fusion protein that behaves as a histone superacetylator, resulting in chromatin hyperacetylation that completely alters the epigenetic landscape of the affected cells (Reynoird et al, 2010). Finally, gene expression profiling in malignant brain tumours derived by inactivation of the *l(3)mbt* tumour suppressor in Drosophila, has revealed predominant expression of germline specific transcripts some of which were found to be essential for tumour growth (Janic et al, 2010).

**Figure 1:**
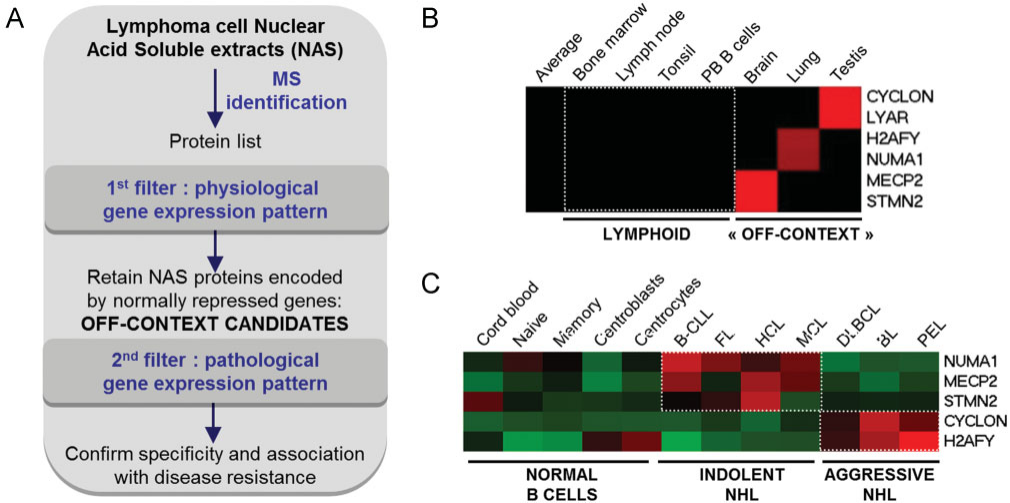
**Proteomics-based discovery strategy for identification of abnormally expressed nuclear factors in lymphoma**Schematic representation of the overall experimental strategy, as indicated. NAS: nuclear acid-soluble, MS: mass spectrometry.Heat-map representation of the gene expression levels for 6 candidate ‘off-context’ genes in normal lymphoid and non-lymphoid tissues; PB: peripheral blood [data from BioGPS (Su et al, 2004)].Heat-map representation of gene expression profiles for 5 ‘off-context’ genes, as indicated; NHL: non-Hodgkin lymphoma, B-CLL: B-cell chronic lymphocytic leukemia, FL: follicular lymphoma, HCL: hairy cell leukemia, MCL: mantle cell lymphoma, BL: Burkitt lymphoma, DLBCL: diffuse large B-cell lymphoma, PEL: primary effusion lymphoma [data from GEO GSE2350 (Basso et al, 2005)].

In this setting, we have devised a novel, transcriptome-driven, proteomics approach to identify candidate, ‘off-context’ factors likely to be involved in tumour progression and immuno-chemotherapy resistance in lymphoma (Fig 1A). Specifically, we geared our screening procedures to identify abnormally expressed, positively-charged, nuclear factors as these were likely to bind nucleic acids and to directly or indirectly engage aberrant and potentially druggable gene regulatory pathways in lymphoma. By using this strategy, we have discovered a novel Rituximab resistance mechanism that depends on MYC-driven overexpression of the nuclear, male germ cell factor, CYCLON. Remarkably, this resistance can be relieved by treatment with small molecule inhibitors of BET bromodomain-dependent chromatin signalling. Furthermore, CYCLON was identified to be an autonomous tumour growth driver that cooperates with MYC to drive aggressive lymphoma growth *in vivo* thus further rationalizing the value of therapeutically targeting this factor in lymphoid malignancies.

## RESULTS

### Proteomics-based identification of nuclear factors that show unscheduled activation in lymphoma

To identify candidate nuclear factors associated with aggressive disease and treatment resistance in lymphoma, we first derived an inventory of positively charged nuclear proteins in the DLBCL line, B593. This lymphoma cell line was chosen as it harbours both *BCL2* and *MYC* gene translocations (Le Baccon et al, 2001) and therefore represents a good model system for the investigation of disease mechanisms in aggressive ‘double hit’ DLBCL. The inventory of positively charged nuclear proteins, in B593 DLBCL cells, was achieved by extracting the acid soluble fraction (Supporting Information Fig S1A) of nuclear proteins (NAS fraction) followed by mass spectrometry (MS) sequencing of the isolated proteins (Fig 1A and Supporting Information Fig S1A, B). Due to enrichment of the acid-soluble proteome in nucleic acid binding factors, we reasoned that we should also find, within this particular proteome, deregulated factors capable of impacting gene/protein expression and contributing to malignant transformation. Indeed, our previous work on acid soluble fractions from male germ cells (Govin et al, 2006) had shown that this fraction is highly enriched in nucleic acid-binding factors, since the majority of identified proteins were known or potential DNA and RNA binding factors. This also showed that charge interaction between positively charged proteins and negatively charged nucleic acids is a prevalent determinant of protein–nucleic acid interactions in nature. In keeping with this, MS sequencing of the NAS protein fraction in our model DLBCL cell line, identified a total of 236 proteins of which a majority represented nuclear DNA/RNA binding proteins (Supporting Information Fig S1B and Table S1). The next step was to identify those factors that showed clear evidence of ‘off-context’ expression in the reference DLBCL lymphoma cell line. For this, normal tissue and cell type specific gene expression data were retrieved from the BioGPS database (which contains expression data for 79 normal tissues/cell types) for each of the 236 NAS proteins (Supporting Information Fig S1C). ‘Off-context’ factors were defined by a level of expression in at least one non-lymphoid normal tissue that was greater than 10-fold the median expression value across all of 79 normal tissues/cell types. By using this stringent cut-off, 6 factors expressed predominantly in brain (*MECP2*, *STMN2*), lung (*H2AFY*, *NUMA1*), and testis (*CYCLON*, *LYAR*), were identified as showing abnormally high expression in the index DLBCL cell line (Table 1 and Fig 1B). It is noteworthy, that for two of these factors (CYCLON and H2AFY) spectral counts in the NAS proteome of the reference DLBCL cell line were close to those observed for core histones (Supporting Information Fig S1D), reflecting their remarkably high abundance in the B593 NAS fraction. Subsequently, interrogation of publically available data sets (Basso et al, 2005) for 5 of these 6 genes (probes were unavailable for *LYAR*) confirmed low or absent expression in normal B-cell subsets and high expression in either indolent (*NUMA1*, *MECP2*, *STMN2*) or aggressive lymphoid malignancies (*H2AFY*, *CYCLON*), including BL, DLBCL and primary effusion lymphoma (PEL), respectively (Fig 1C). Taken together, these findings validate the clinical relevance of our biomarker discovery approach and identify candidate nuclear factors of potential relevance to the pathogenesis of lymphoid malignancies.

**Table:** ‘Off-context’ candidates identified in the nuclear acid soluble fraction from the DLBCL line, B593

Gene name	Protein description	Cellular component	Biological process	Predominant normal tissue expression	Spectral counts	Position in B596 NAS fraction (sorted by spectral counts)
CCDC86	Cytokine-induced protein with coiled-coil domain	Nucleus	Unknown	Testis	106	22/236
H2AFY	Core histone macro-H2A.1	Nucleus	Chromatine Structure	Lung	42	44/236
MECP2	Methyl-CpG-binding protein 2	Nucleus	Chromatine Structure	Brain	16	85/236
NUMA1	Nuclear mitotic apparatus protein 1	Nucleus	Cell cycle	Lung	14	90/236
LYAR	Cell growth-regulating nucleolar protein	Nucleus	Cell cycle	Testis	10	106/236
STMN2	Stathmin-2	Cytoskeleton	Microtubule polymerization	Brain	1	219/236

### *CYCLON* overexpression is linked to adverse clinical outcome in immunochemotherapy-treated DLBCL

**Figure 2:**
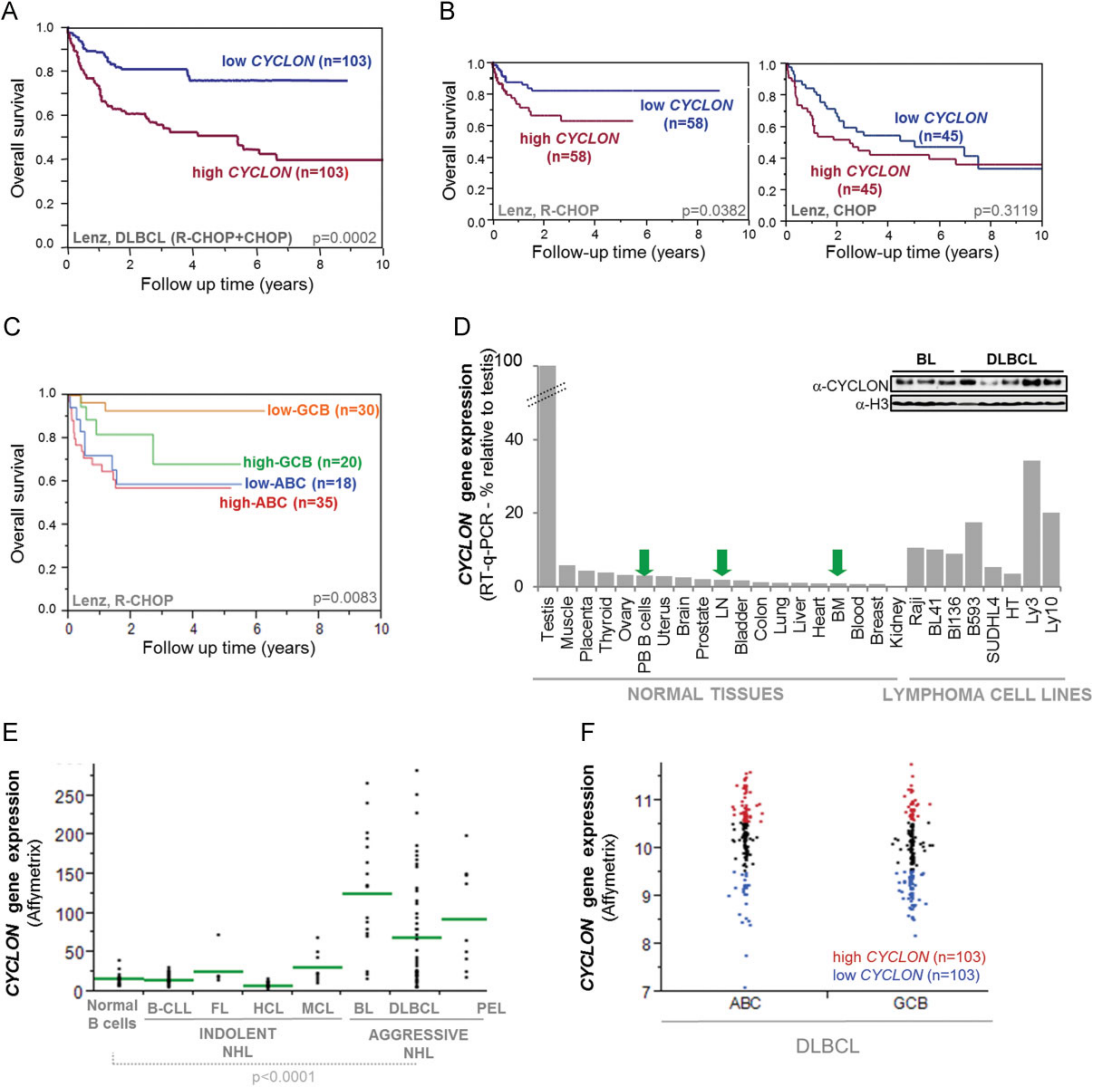
***CYCLON* overexpression is a feature of aggressive NHL and correlates to adverse clinical outcome in DLBCL**Kaplan–Meier cumulative survival curves in DLBCL patients from GEO GSE10846 (Lenz et al, 2008) according to CYCLON expression level (high: above 3rd quartile and low: below 1st quartile, respectively), p values from log-rank test.Survival curves according to CYCLON expression levels in DLBCL patients treated by Rituximab-CHOP (left panel, R-CHOP) or CHOP alone (right panel), *p*-values from log-rank test.Survival curves according to CYCLON expression levels in ABC or GCB DLBCL patients treated by Rituximab-CHOP, *p*-values from log-rank test.RT-qPCR and western blot (upper panel) analysis of *CYCLON* expression in normal lymphoid and non-lymphoid tissues, and in lymphoma lines, as indicated: PB B: peripheral blood B cells, LN: lymph node, BM: bone marrow, *n* = 3.Affymetrix-derived *CYCLON* gene expression values for individual patients from GEO GSE2350 (Basso et al, 2005), *p*-value from Wilcoxon test (E) and GEO GSE10846 (Lenz et al, 2008) (F).

CYCLON is a novel, cytokine-inducible, nuclear phosphoprotein (Hoshino & Fujii, 2007) that bears a coiled coil domain, also shared with many transcription factors, whereas *H2AFY* encodes a histone variant that has been shown to influence prognosis in malignant melanoma and lung cancer (Kapoor et al, 2010; Sporn et al, 2009). Given their potential role in driving aberrant gene expression in cancer, *H2AFY* and *CYCLON* were selected for further analysis of their clinical relevance in lymphoma. Using publically available, clinically annotated transcriptomic data in DLBCL, we tested the association between overexpression of *H2AFY* and *CYCLON* and survival (Lenz et al, 2008). We identified high-level expression of *CYCLON* mRNA, but not of *H2AFY*, to be significantly correlated to poor survival in DLBCL (Fig 2A and Supporting Information Fig S2A). Significantly, high level CYCLON expression was associated with inferior outcome in R-CHOP but not CHOP-treated DLBCL patients (Fig 2B and Supporting Information Fig S2B). Further survival analysis, according to DLBCL molecular subtype, showed a marked tendency towards poor survival in R-CHOP-treated DLBCL of the GCB but not the ABC subtype (Fig 2C). High CYCLON expression was not associated to poor survival in CHOP-treated patients of either molecular subtype (Supporting Information Fig S2C). Taken together this indicated that *CYCLON* could be a novel Rituximab response factor, at least in DLBCL of the GCB subtype. RT-qPCR analysis in a panel of normal tissues and lymphoma lines subsequently revealed high *CYCLON* expression in testis and in DLBCL and BL, as expected from our initial analysis, and low expression elsewhere including normal lymph node and peripheral blood B cells (Fig 2D). This was confirmed by gene expression analysis in additional publically available transcriptomic data sets (Basso et al, 2005) (Fig 2E), which further revealed high *CYCLON* expression in both the GCB and ABC subtypes of DLBCL (Lenz et al, 2008) (Fig 2F). Protein abundance and mRNA levels in B-cell lymphoma lines showed good correlation, indicating that *CYCLON* mRNA expression reflects protein expression (Fig 2D, upper panel).

### CYCLON is a MYC target that constrains MYC-dependent tumour growth *in vivo*

**Figure 3:**
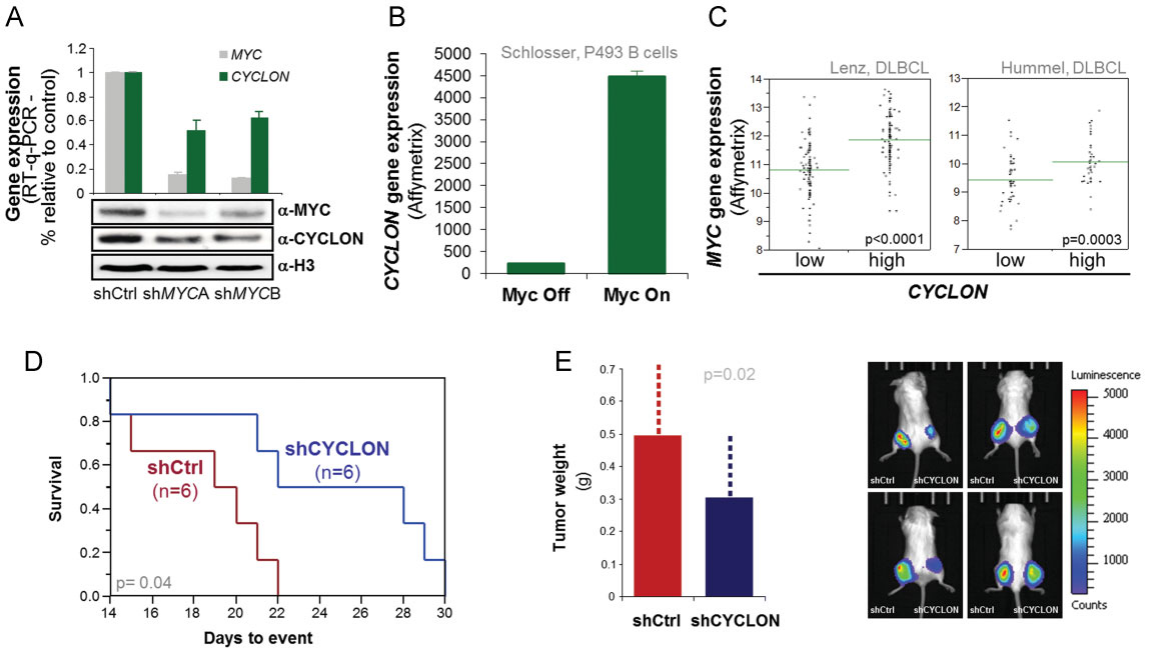
**CYCLON is a MYC target involved in lymphoma progression *in vivo***Analysis by RT-qPCR and western blot (upper and lower panels, respectively) of CYCLON expression in control and MYC-knockdown Raji lymphoma B cells, *n* = 3.*CYCLON* gene expression values in the MYC-inducible cell line, P493 (Schlosser et al, 2005).Affymetrix-derived *MYC* gene expression values for DLBCL in GEO GSE10846 (Lenz et al, 2008) and GEO GSE4475 (Hummel et al, 2006); high *CYCLON* expression: above 3rd quartile; low *CYCLON* expression: below 1st quartile, *p*-values from Student t test.Kaplan–Meier cumulative survival curves for xenotransplanted mice bearing tumours derived from Raji shCtrl and Raji shCYCLON cell lines, *p*-value from log-rank test.Tumour weight (28 days post-injection) for xenotransplanted mice bearing tumours derived from Raji shCtrl and Raji shCYCLON cell lines, *n* = 11 for each group, *p*-value from Wilcoxon test (left) and bioluminescence imaging for 4 representative mice (right).

High CYCLON expression was observed in BL, an MYC-dependent lymphoma (Mertz et al, 2011), and in aggressive DLBCL (of both the GCB and ABC subtypes), but not in other lymphoid malignancies. We thus considered whether CYCLON expression might be dependent on MYC. In keeping with this, MYC knockdown in Raji BL cells resulted in down-regulation of CYCLON at both the mRNA and protein levels (Fig 3A). This was in agreement with results in a gene expression data set from a MYC-inducible B-cell line (Schlosser et al, 2005), where MYC activation led to marked up-regulation of the *CYCLON* gene (Fig 3B). In addition, high *CYCLON* expression was significantly associated with high *MYC* expression in DLBCL of both the GCB and ABC subtypes, suggestive of co-selection of these two events in the pathogenesis of DLBCL (Fig 3C) (Lenz, *p* < 0.0001, Hummel, *p* = 0.0003). Taken together, these data raised the possibility that CYCLON is a key downstream effector of oncogenic MYC signalling in lymphoma. In support of this, SCID mice engrafted with CYCLON-knockdown (transduced by shCYCLON) Raji BL cells (Supporting Information Fig S3A) showed significantly reduced tumour size and increased survival (Fig 3D, E), compared to mice engrafted with control Raji cells (transduced by non-targeting shRNA). CYCLON knockdown did not alter MYC levels in Raji or other lymphoma lines tested (Supporting Information Fig S3). This indicates that CYCLON overexpression is an autonomous tumour growth driver that cooperates with MYC to drive lymphoma progression.

### CYCLON is a novel transcriptional regulator that controls gene expression programs of biological relevance to lymphoma pathogenesis

**Figure 4:**
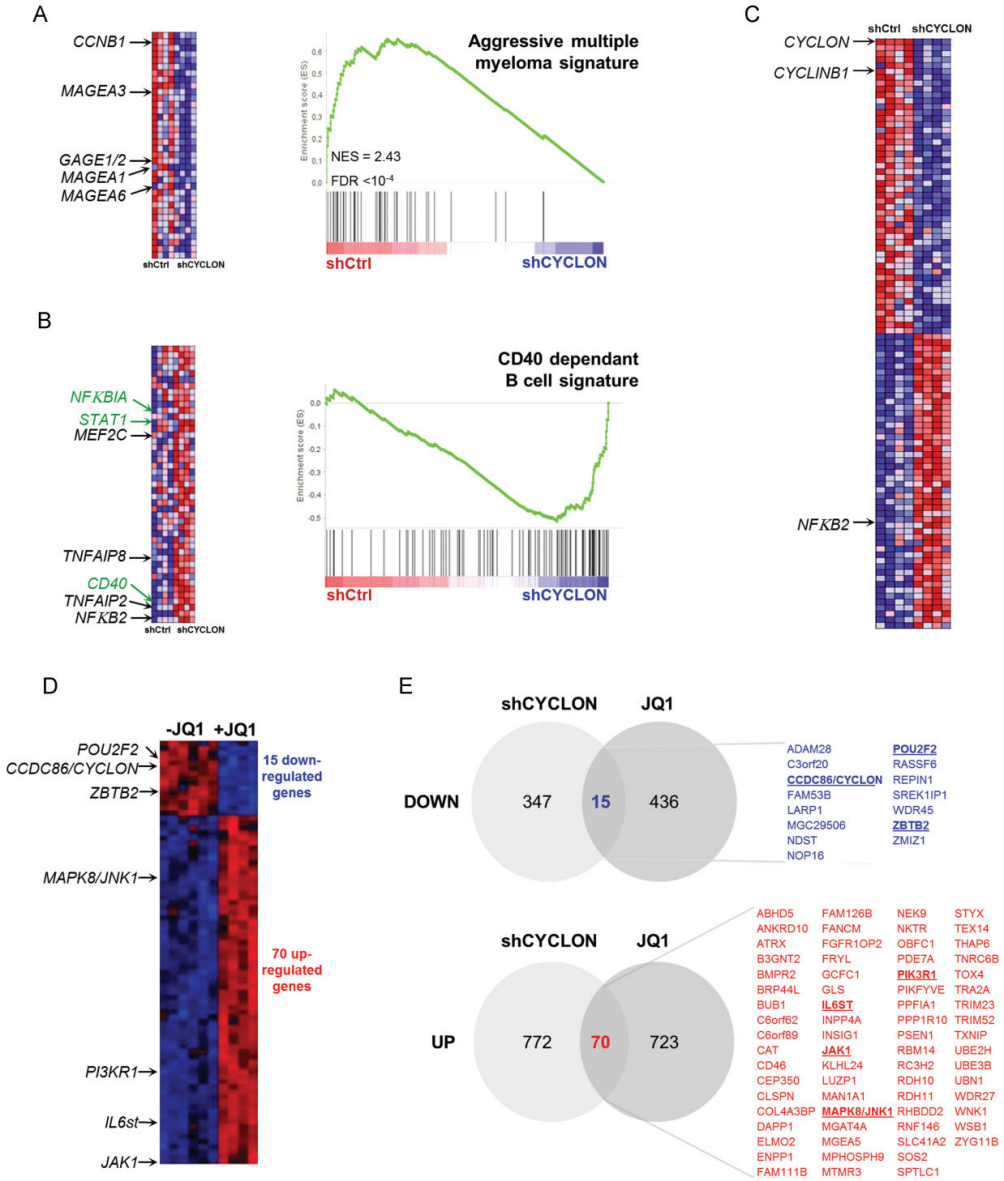
***CYCLON* regulates gene signatures associated with B-cell differentiation and proliferation**Gene Set Enrichment Analysis (GSEA) enrichment plots obtained from gene expression data from CYCLON knockdown Raji cells (shCYCLON, *n* = 4) compared to control Raji cells (shCTRL, *n* = 4) with associated heat-maps (left). GSEA enrichment plots show down-regulation of aggressive multiple myeloma signature (A) and up-regulation of a CD40-dependent B-cell signature (B) in CYCLON knock-down compared to control Raji lymphoma B cells. Genes are ordered on the x axis by their differential expression score (each vertical line representing a gene).Top 50 genes up-regulated (red) or down-regulated (blue) upon CYCLON knockdown (shCYCLON) in Raji lymphoma B cells compared to control (shCtrl). Gene sets are from MSIGDB collection. See the Materials and Methods section and supplementary material for detailed data analysis.Heat-map representation of co-regulated genes upon CYCLON knockdown and JQ1 treatment in Raji cells (data from GSE29449, Mertz et al, 2011).Venn diagram showing the overlap between differentially expressed genes upon CYCLON knockdown and JQ1 treatment in Raji cells (data from GSE29449, Mertz et al, 2011) and list of commonly down- and up-regulated genes. Black and green arrows: genes cited in text. Green arrows refer to genes commonly regulated by CYCLON and JQ1 within the CD40 signature.

To gain insight into the possible function of CYCLON in the pathogenesis and treatment resistance of B-cell malignancies, we performed gene expression profiling in CYCLON-knockdown or control Raji BL cells. This analysis revealed a total of 1204 differentially expressed genes (unpaired *t*-test with Benjamini–Hochberg correction; corrected p value <0.05 and fold change ≥1.2), including 842 up-regulated and 362 down-regulated genes, in the knockdown compared to the control cells (Supporting Information Fig S4 and Table S2), indicating that CYCLON has both activating and repressive effects but predominantly functions in gene repression. We then performed unbiased gene set enrichment analysis (GSEA) to identify gene signatures (*i.e*. pre-defined lists of genes that are associated to a specific biological function) regulated by CYCLON. This analysis revealed significant down-regulation by CYCLON knockdown of a proliferation gene expression signature that includes cancer testis antigens (e.g. *MAGEA1*, *MAGEA3*, *MAGEA6*, *GAGE1*), and that characterizes an aggressive subtype of multiple myeloma (Zhan et al, 2006) (Fig 4A and Supporting Information Table S3). GSEA also revealed up-regulation of a previously-defined, CD40-regulated, mature B-cell-specific gene expression signature (Basso et al, 2004), in the CYCLON-knockdown compared to control lymphoma Raji BL cells (Fig 4B and Supporting Information Table S4). This is of interest since the germinal centre B-cell is the presumed cell of origin for both DLBCL and BL. The CD40-regulated ‘mature B-cell signature’ comprises genes of known importance in DLBCL/BL pathogenesis including *NFKB2*, *MEF2C* (Wilker et al, 2008) and *TNFAIP* genes (Compagno et al, 2009; Kato et al, 2009; Lohr et al, 2012). A number of these signature genes were among the top 50 CYCLON up or down-regulated genes (Fig 4C). Collectively, these data identify CYCLON as a transcriptional regulator whose ‘off-context’ expression drives gene expression programs of biological relevance to lymphoma pathogenesis.

### CYCLON modulates Rituximab sensitivity *in vitro* and *in vivo*

**Figure 5:**
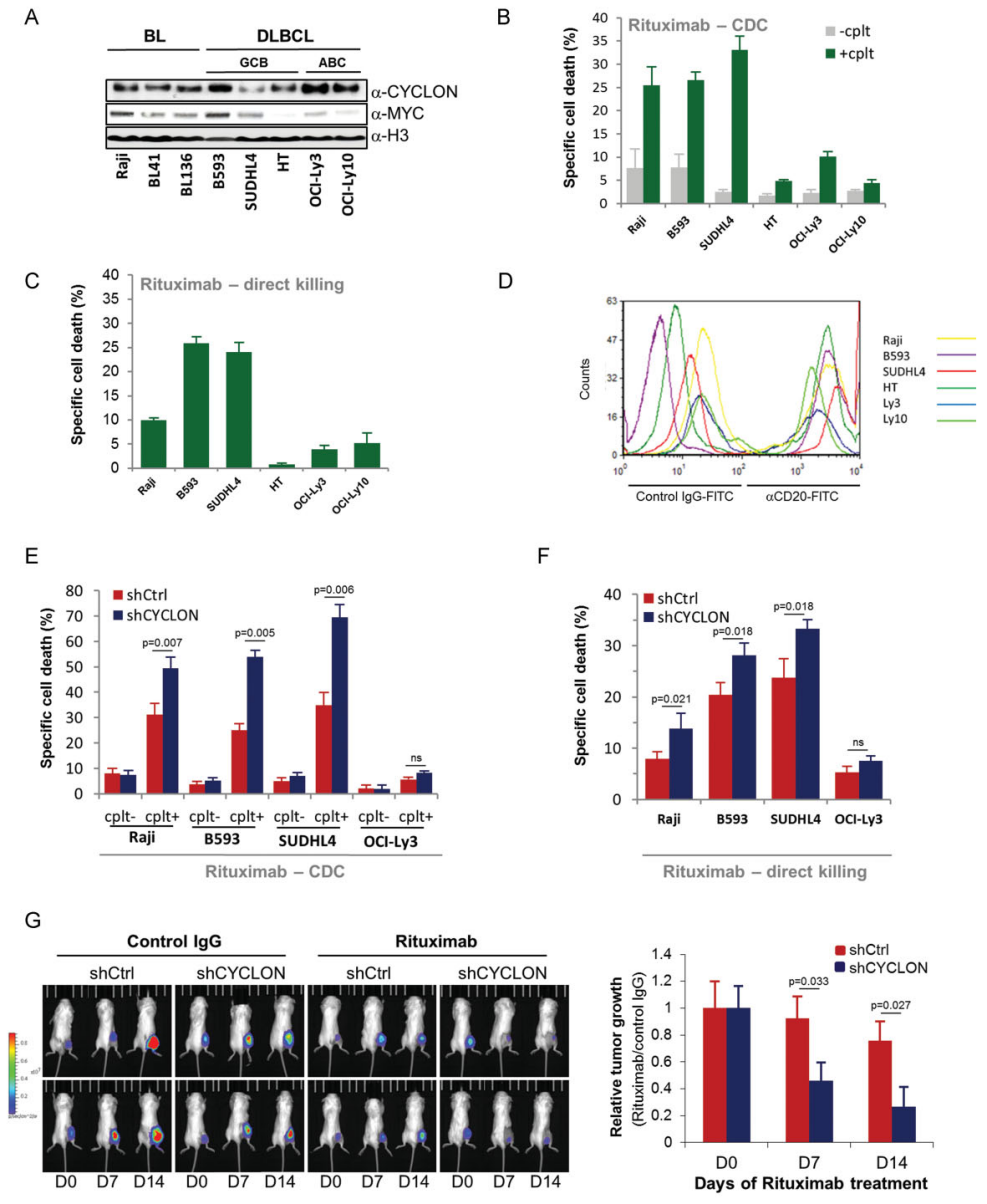
**CYCLON-mediated resistance to Rituximab in BL and MYC-overexpressing DLBCL**Western Blot analysis of CYCLON, MYC and histone H3 (as loading control) expression in levels in a panel of BL and DLBCL cell lines.Evaluation of basal Rituximab sensitivity using CDC (complement dependent cytoxicity) in the presence (+cplt) or absence of human complement (−cplt) (B) and Rituximab-induced direct killing (C) assays as described in the Materials and Methods section, *n* = 6.Flow cytometry analysis of CD20 expression in a panel of BL and DLBCL cell lines, *n* = 3.Rituximab (E: CDC; F: direct killing) sensitivity of CYCLON knockdown (shCYCLON) Raji (BL), B593, SUDHL4 and OCI-Ly3 (DLBCL) lymphoma B cells compared to controls (shCtrl); p values from a Wilcoxon test, *n* = 8 for each cell line, ns: non-significant.Left panel: bioluminescence imaging of Rituximab response (200 µg/day of Rituximab) in SCID mice xenotransplanted with shCtrl or CYCLON knockdown Raji lymphoma as indicated compared to control-treated mice (IgG for 14 days). Right panel: Relative tumour growth at days 0, 7 and 14, as indicated; *p*-value from a Wilcoxon test, *n* = 6 for each group.[Correction added after publication on 4 July 2013: The figure title “CYCLON-mediated increase in Rituximab sensitivity in BL and MYC-dependent DLBCL” was corrected to “CYCLON-mediated resistance to Rituximab in BL and MYC-overexpressing DLBCL”]

High *CYCLON* expression was significantly associated to high MYC expression and was correlated with inferior clinical outcome in R-CHOP but not CHOP-treated DLBCL patients, particularly of the GCB-subtype. This suggested a role for CYCLON as a key MYC effector in modulating treatment response, in particular to Rituximab, at least in BL and DLBCL of the GCB subtype. To investigate this in more detail we first looked at CYCLON and MYC expression in a panel of BL (Raji, BL41, BL136) and DLBCL cell lines of both the GCB (B593, SUDHL4, HT) and ABC subtypes (OCI-LY3, OCI-LY10) (Fig 5A). Coincident CYCLON and MYC deregulation was detected in 5 of 8 lymphoma cell lines tested (BL cell lines Raji, BL41 and BL136 and in the GCB-type DLBCL, B593 and SUDHL4) (Fig 5A). Three of eight lymphoma cell lines (but no BL line) showed moderate (HT) to high CYCLON overexpression in the absence of marked MYC overexpression, thus suggesting the existence of alternate routes to CYCLON overexpression in these cells. We next tested sensitivity to Rituximab-mediated, complement-dependent cytotoxicity (CDC, Fig 5B) and direct killing (Fig 5C) in a selection of these cell lines. In the conditions used for these assays, lymphoma cells that showed coincident CYCLON and MYC deregulation (Raji, B593, SUDHL4 cells, respectively) were sensitive to both Rituximab-induced, complement-mediated cytotoxicity and direct killing while the remaining cell lines were resistant (Fig 5B, C). The observed effects were not related to differences in the cell surface expression of the Rituximab target receptor, CD20, which was broadly similar across all cell lines (Fig 5D). In order to further explore the role of CYCLON in Rituximab response in lymphoma, short hairpin RNA was used to deplete CYCLON in Raji, B593 and SUDHL4 as well as in OCI-Ly3 cells. Consistent with a role for CYCLON in Rituximab response in lymphoma, CYCLON depletion markedly increased the sensitivity of B593, SUDHL4 (DLBCL cell lines) and Raji (BL) lymphoma cells to Rituximab-dependent killing, particularly complement-mediated cytotoxicity (Fig 5E, F and Supporting Information Fig S5A, B). In contrast, CYCLON depletion was not sufficient to relieve Rituximab resistance in the OCI-Ly3 cell line. This suggests a requirement for even lower CYCLON thresholds to restore Rituximab response and/or the existence of alternative resistance routes, in these cells. CYCLON depletion was not associated to increased antibody-dependent, cell-mediated cytotoxicity, at least in Raji cells (ADCC, Supporting Information Fig S5C). CYCLON depletion did not increase the apoptotic responses in any of the cell lines, following treatment by etoposide (Supporting Information Fig S5D) or CD95/FAS receptor engagement (Supporting Information Fig S5E), which CYCLON is known to regulate, at least in T cells (Saint Fleur et al, 2009). This indicated that CYCLON overexpression directly drives tumour cell intrinsic mechanisms that specifically impact on Rituximab response. To test the relevance of this mechanism *in vivo*, we subcutaneously injected SCID mice with Raji cells expressing either CYCLON targeting or non-targeting shRNA. Once tumours were established, mice were treated with daily injections of either Rituximab or control antibody for a period of 14 days. Rituximab monotherapy significantly reduced the tumour burden in mice engrafted with shCYCLON compared to control lymphoma cells (Fig 5G). Together, these data identify CYCLON as a novel Rituximab response modulator of interest for therapeutic targeting in lymphoma.

### BET bromodomain inhibition increases Rituximab sensitivity in CYCLON and MYC overexpressing lymphoma cells

**Figure 6:**
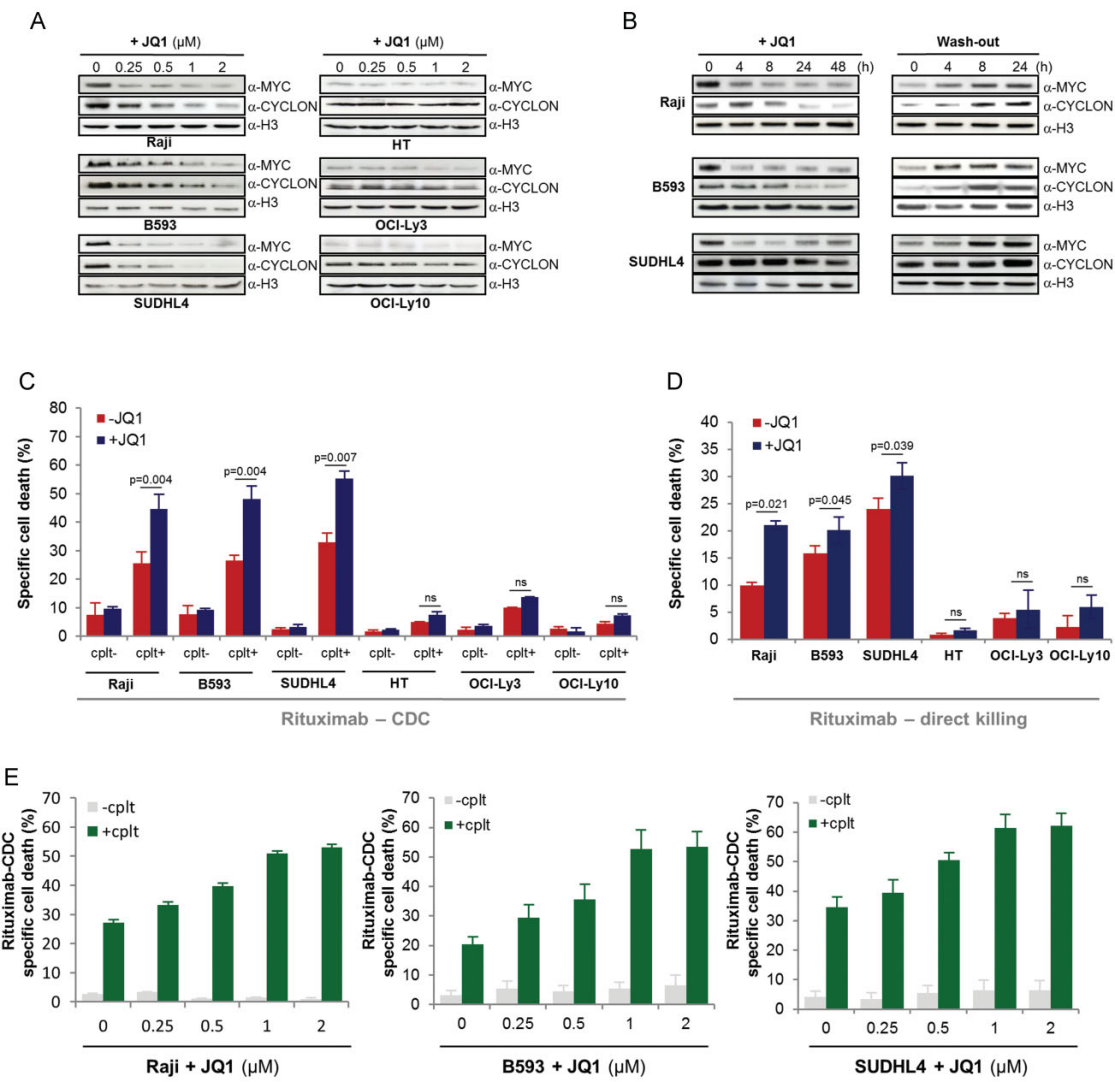
**BET bromodomain inhibition overrides CYCLON-mediated Rituximab resistance**Western Blot analysis of CYCLON, MYC and H3 expression (as loading control) in lymphoma B cells treated for 24 h with control DMSO (0) or increasing doses of JQ1 as mentioned, *n* = 3.Western Blot analysis of CYCLON, MYC and H3 expression (as loading control) in lymphoma B cells treated with control DMSO (0) or 500 nM JQ1 for the indicated times after treatment and wash-out, *n* = 3.Rituximab (CDC, C; direct killing, D) sensitivity assays of lymphoma B cells after 24 h treatment with control DMSO (JQ1-) or 1 µ JQ1 pre-treatment performed as described in Fig 4E, F; *p*-values from a Wilcoxon test, *n* = 4 for each cell line, ns: non-significant.Evaluation of Rituximab sensitivity (CDC) of Raji cells pre-treated for 24 h with control DMSO (0) or increasing doses of JQ1 as mentioned, *n* = 3 for each cell line.

Small molecule inhibitors of BET bromodomain proteins (JQ1, iBET) have recently been shown to attenuate MYC-driven oncogenic signalling in lymphoma and myeloma (Delmore et al, 2011; Mertz et al, 2011; Zuber et al, 2011). We thus tested whether one such inhibitor, JQ1, could relieve CYCLON overexpression and associated suboptimal Rituximab responses in lymphoma. We first performed a dose-response experiment to test the effects of JQ1 on MYC levels in our panel of lymphoma cell lines. In keeping with previous reports (Mertz et al, 2011), JQ1 treatment induced pronounced, dose-dependent MYC suppression in MYC-overexpressing lymphoma cells (Raji, B593, SUDHL4) while suppressive effects were more subtle in the lymphoma cell lines with lower starting levels of MYC (HT, OCI-Ly3 and Ly10) (Fig 6A). We next monitored the effects of JQ1 on CYCLON expression in the same cells. Based on our previous results showing that CYCLON can be regulated by MYC (Fig 3A–C), we predicted that JQ1 would override CYCLON overexpression in lymphoma cells that showed concomitant MYC deregulation. In keeping with this, CYCLON suppression, consistently proceeding MYC down-regulation, was observed in the Raji, B593 and SUDHL4 cells but not in the HT, OCI-Ly3 or OCI-Ly10 cells, which show comparatively low starting levels of MYC (Fig 6A), thus suggesting alternative, BET bromodomain inhibitor-insensitive routes to CYCLON deregulation in these cells. MYC and CYCLON suppression was reversible and occurred in sequence, as shown by JQ1 washout experiments in the sensitive lymphoma cells (Raji, B593 and SUDHL4) (Fig 6B). In these lymphoma lines, it thus seems that JQ1 treatment regulates CYCLON indirectly through MYC rather than through BRD4 – or indeed through BRD2 and 3 which JQ1 can also inhibits (Filippakopoulos et al, 2010). The next step was to explore the effects of JQ1 on Rituximab response *in vitro*. For this, the same 6 lymphoma B-cell lines, were pre-treated for 24 h with 1 µM JQ1 and then challenged with Rituximab. JQ1 pre-treatment alone, under these conditions, did not induce cell death in any of the cell lines tested (Supporting Information Fig S7A) nor did it significantly alter responses to genotoxic agents such as etoposide (Supporting Information Fig S7B). Strikingly, JQ1 pre-treatment followed by Rituximab challenge led to significant and uniform increases in Rituximab-mediated killing of lymphoma cells presenting JQ1-sensitive MYC and CYCLON deregulation (Raji, B593 and SUDHL4), but not in lymphoma cells that showed JQ1-refractory CYCLON overexpression (HT, OCI-Ly3 and Ly10) (Fig 6C, D). Rituximab responses in JQ1-sensitized lymphoma cells were dose-dependent up to 1 µm JQ1 (Fig 5E). Taken together, these data identify a new BET bromodomain inhibitor-sensitive Rituximab response pathway that is controlled by a MYC and CYCLON-dependent gene regulatory circuit in lymphoma.

### Convergent biological pathways regulated by CYCLON and BET bromodomain inhibition in lymphoma B cells

In view of the fact that CYCLON acts as an independent tumour growth driver in MYC-driven lymphoma, and that MYC and CYCLON overexpression controls a JQ1-sensitive Rituximab resistance pathway, we next wished to investigate the possible links between CYCLON activity and BET bromodomain inhibition. For this we compared the gene expression profiles of CYCLON knockdown Raji lymphoma B cells with publically available gene expression data for JQ1-treated, Raji lymphoma B cells (Mertz et al, 2011). This analysis identified a discrete set of 85 genes that are commonly regulated by JQ1 treatment and CYCLON knockdown (15 and 70 genes, respectively, down- and up-regulated following a JQ1 treatment or shCYCLON) (Fig 4D), in addition to numerous genes that are independently regulated under either condition. The core set of commonly down-regulated genes included *ZBTB2*, which encodes a negative regulator of p53 signalling (Jeon et al, 2009) and *POU2F2*, which encodes a homeobox transcription factor involved in the germinal centre reaction, in response to viral infection (Karnowski et al, 2012) and that has recently been shown to be mutated in DLBCL (Zhang et al, 2013) (Fig 4D, E). The core set of commonly up-regulated genes included a number that encode factors involved in pathways of direct relevance to normal and pathological B-cell signalling (IL6st, JAK1 and PIK3R1, for example) (Love et al, 2012; Ngo et al, 2011) as well as JNK1 which has been implicated in the regulation of complement dependent cell death (Gancz et al, 2009). Closer inspection of GSEA signatures across both conditions revealed, as expected, predominant down-regulation of MYC signatures/target genes, including CYCLON itself, in JQ1-treated but not CYCLON knockdown lymphoma B cells. Interestingly, co-regulation by JQ1 and CYCLON of a subset of genes that comprise the previously identified CYCLON-regulated CD40 signature was also seen. This gene list included the CD40 gene itself as well as genes encoding signalling factors such as the NFKB inhibitor, NFKBIA and STAT1 (Fig 4B).

## DISCUSSION

Combination immunochemotherapy using anti-CD20 antibodies and cytotoxic drugs has led to marked improvements in treatment response and overall survival in B-NHL. However, treatment resistance and relapse remain problematic. This is spurring the development of alternative treatment strategies, including novel therapeutic antibodies, and targeted therapy strategies aimed at curtailing oncogene dependency pathways in lymphoid neoplasia (Friedberg, 2011). A major confounding factor in these efforts, however, is the lack of knowledge on the cellular and molecular mechanisms that contribute to treatment resistance. In particular, the nature of signalling pathways that directly or indirectly control biological and clinical responses to anti-CD20 therapy are not well elucidated (Cheson & Leonard, 2008).

Here, by performing a concept-driven targeted proteomics and functional analyses, based on the ectopic activation of tissue-specific genes in cancer, we have identified a Rituximab resistance mechanism that operates in aggressive B-cell lymphoma and that is mediated by MYC-driven overexpression of the nuclear factor, CYCLON. Remarkably, this resistance mechanism could be alleviated by next generation epigenetic therapy, using the BET bromodomain small molecule inhibitor, JQ1. JQ1 functions as a specific inhibitor of BET bromodomain binding of acetylated lysines in chromatin and has demonstrated single agent efficacy in diverse cancer models (Delmore et al, 2011; Filippakopoulos et al, 2010; Mertz et al, 2011; Ott et al, 2012; Zuber et al, 2011) but has not previously been tested in combination treatment with a therapeutic monoclonal antibody.

CYCLON suppression specifically sensitized lymphoma cells to Rituximab-dependent CDC and direct killing, but not to ADCC or indeed other apoptotic stimuli. This suggests the regulation by CYCLON of cell intrinsic mechanisms downstream of direct CD20 engagement and/or Rituximab Fc fragment activity which is required for complement activation but is dispensable for CD20 signalling *per se* (Vega et al, 2009). BET bromodomain inhibition appears to break Rituximab resistance in lymphoma B cells showing coincident MYC and CYCLON overexpression. This could operate by reducing CYCLON levels to critically low thresholds, which might affect key, high-affinity, CYCLON target genes directly or indirectly impacting on Rituximab response. In keeping with this, CYCLON and JQ1 regulate a number of genes that encode factors of relevance to lymphoma B-cell survival, including the inhibitor of NFKB signalling, NFKBIA as well as JNK1, known to be involved in complement-mediated cytotoxicity (Gancz et al, 2009; Jeon et al, 2009). Noteworthy, is that constitutive NFKB signalling has been linked to experimental resistance to Rituximab in lymphoma cell lines (Jazirehi et al, 2007). One could also bear in mind that maintenance therapy with Rituximab alone is being explored in DLBCL (Michallet et al, 2012). Association of Rituximab with BET bromodomain inhibitors in this setting might be an attractive therapeutic option.

We also identified Rituximab-resistant, lymphoma B cells that overexpress CYCLON, without concomitant MYC deregulation, thus pointing to the existence of other as yet un-identified routes to CYCLON overexpression in lymphoma. BET bromodomain inhibition does not relieve Rituximab resistance in these cells suggesting that coincident MYC and CYCLON overexpression could be used as a biomarker to identify patients most likely to respond to BET bromodomain inhibitor and Rituximab combination therapy.

CYCLON has been shown to play a key role in maintaining normal T-cell homeostasis following cellular activation (Saint Fleur et al, 2009). This occurs through up-regulation, by CYCLON, of CD95 expression in activated but not resting T cells. The CD95 pathway is also involved in maintaining B-cell homeostasis downstream of B-cell receptor signalling and in the germinal centre reaction (Hao et al, 2008). It is thus possible that CYCLON also regulates CD95 expression in discrete stages of normal B-cell development. Indeed, we uncovered CYCLON expression, albeit at low levels, in normal GC B-cell subsets. CYCLON might also contribute to the control of mature B-cell development downstream of MYC. In support of this notion, normal germinal center B-cell subsets, defined by either high or low MYC levels have recently been identified (Calado et al, 2012; Dominguez-Sola et al, 2012). Significantly, at least in mice, normal ‘high MYC’ GC B cells are characterized by a specific gene expression signature, that includes numerous B-cell activation response genes (including CD40 signalling components), and up-regulation of Cyclon/Ccdc86 (Dominguez-Sola et al, 2012).

Normal, ‘high MYC’, GC B cells are essential for both the early and late phases of the germinal center reaction and appear to be poised for re-entry to the dark zone for further rounds of affinity maturation (Calado et al, 2012; Dominguez-Sola et al, 2012). We have shown that CYCLON deregulation is predominant, albeit not exclusive to, ‘high MYC’ DLBCL tumours, of both the GCB and ABC subtypes. At least for the former, it is tempting to speculate that CYCLON deregulation occurs as ‘collateral damage’ downstream of MYC deregulation, by chromosomal translocation or other event (MYC gene rearrangement is common DLBCL of the GCB subtype). This would uncouple not only MYC, as previously postulated (Dominguez-Sola et al, 2012), but also CYCLON-dependent gene regulatory circuits, from normal affinity maturation, thus facilitating malignant reprogramming of the affected B cells, ultimately leading to disturbed GC dynamics and lymphomagenesis. In this respect, it is noteworthy that CYCLON regulates gene expression signatures of direct relevance to the pathogenesis of germinal center-derived lymphoma. Of specific interest is CYCLON-mediated suppression of *NFKB2* expression. *NFKB2* is also a MYC repression target and *Nfkb2* loss has been shown to accelerate lymphoma development in Eµ-*Myc* transgenic mice (Keller et al, 2010). This works suggests that BET bromodomain inhibition could override these oncogenic signalling events.

In conclusion, we have devised a proteomics-driven strategy to leverage illegitimate gene activation events for the discovery of new treatment strategies in high risk B-cell lymphoma. A remarkable result was the identification of a MYC-controlled disease progression and Rituximab resistance regulatory circuit that is autonomously controlled by the nuclear factor CYCLON. This CYCLON-MYC regulatory axis can be blocked by BET bromodomain inhibitors thus opening avenues for new anti-lymphoma treatment strategies based on combining anti-CD20 monoclonal antibodies and next generation epigenetic therapy.

## MATERIALS AND METHODS

### Cell culture, reagents and antibodies

B593 DLBCL cell line was derived in house (Callanan et al, 2000). SUDHL4, HT (DLBCL lines) and Raji (BL line) were purchased from DMSZ. OCI-Ly3 and OCI-Ly10 were kindly provided by Professor Georg Lenz. Cells were cultured in RPMI medium supplemented with 2 mM GlutaMAX®, 1 mM sodium pyruvate, 25 mM HEPES, non-essential amino acids, 100 µg/mL Penicillin/Streptomycin solution and 10% foetal calf serum (Invitrogen), except for OCI-LY3 and OCI-Ly10 which were cultured in IMDM 20% human serum (Cambrex) supplemented with 2 mM GlutaMAX®, 1 mM sodium pyruvate, 25 mM HEPES, non-essential amino acids, 100 µg/mL Penicillin/Streptomycin solution (added with 50 µM β-mercaptoethanol for OCI-Ly3 cell line). Unless otherwise specified, all chemicals were purchased from Sigma–Aldrich. Anti-CYCLON antibody was obtained 28 days after rabbit immunization with both C and N-terminus CYCLON peptides and antibody affinity-purification against the C-terminus peptides from immune serum (Speed Rabbit program, Eurogentec) or purchased from Bethyl Laboratories. Anti-MYC (clone 9E10) and anti-H3 were obtained from Santa Cruz and Millipore, respectively. Rituximab (Mabthera®, Roche) and Herceptin (Trastuzumab®, Roche) were obtained at Grenoble University Hospital pharmacy.

### JQ1 synthesis

(±)-JQ1 was synthesized using the procedure published by Filippakopoulos et al (Filippakopoulos et al, 2010). Highly purified (±)-JQ1 was obtained by column chromatography (gradient *n*-hexane/ethyl acetate 8:2 to 100% ethyl acetate) followed by precipitation from ethyl ether by *n*-hexane. The final compound was characterized by ^1^H NMR (Supporting Information Fig S6A), MS (Supporting Information Fig S6B) and HPLC (using an analytical OD-H chiral column) (Supporting Information Fig S6C). Enantiomeric composition of the crystalline product was verified by chiral HPLC to be 55% active enantiomer (+)-JQ1 (OD-H analytical column; *n*-hexane/2-propanol 85:15; (+)-JQ1 r.t.: 10 min, (−)-JQ1 r.t.: 12 min).

### Protein extraction and mass spectrometry analysis

Whole cell lysates were obtained by sonication of cell pellets in Laemmli buffer. Nuclear acid soluble fractions were obtained by direct sonication of cell nuclei in 0.2 N sulphuric acid (see Supporting Information Fig S1A) as described previously (Govin et al, 2006) and separated by SDS-PAGE (20 µg protein per lane) following Coomassie staining. MS analyses were performed on three biological replicates. Trypsin in-gel digestion and LC-MS/MS analysis were performed as described previously (Dos Santos et al, 2010). MS/MS data were acquired and processed automatically using Xcalibur™ 1.4 software (Thermo Fisher Scientific).

### Protein identification by mass spectroscopy

MS data were analysed using the Mascot 2.1 program (Matrix Science) with compilation of SwissProt and Trembl databases (release 50.1/33.1, 3,192,898 entries in total including 17,483 human, June 2006) using *Homo sapiens* as selected species. The variable modifications allowed are listed at the bottom of Table S1. Mass accuracy was set to 10 ppm for precursor ion and 0.8 Da for fragments. Mascot data were then transferred to IRMa validation software (Dupierris et al, 2009), which allowed data filtering and suppression of protein redundancy on the basis of proteins being identified by a same set or a subset of peptides (Target Decoy approach (Choi et al, 2008): FDR = 1.6%). Manually validated hits from each of the three biological replicates were transferred to a relational database. Protein abundance was estimated using the spectral counting method (Gilchrist et al, 2006). Molecular weight, subcellular localization and associated functions were retrieved for each protein from Uniprot (http://www.expasy.uniprot.org/).

### Gene expression analysis by RT-qPCR

Total RNA was extracted from cell lines, tissues or sorted B cells by TRIzol reagent (Invitrogen) and quantified by NanoDrop (Thermo Fisher Scientific) before being reverse transcribed using the SuperScript® III First-Strand Synthesis SuperMix for RT-qPCR (Invitrogen), according to manufacturer's instructions. qPCR was performed using SYBR® Green PCR master mix (Applied Biosystems) and primers shown in Table S5, according to the manufacturer's instructions. The *ABL* gene was used as a control for normalization of gene expression data. qPCR was performed on an MX3000P (Stratagene) machine.

### Microarray gene expression analysis

Affymetrix microarrays were processed in the Microarray Core Facility of the Institute of Research on Biotherapy, CHRU-INSERM-UM1 Montpellier (http://irb.chu-montpellier.fr/). RNA was amplified using the kit GeneChip IVT express and hybridized to HG U133 Plus 2.0 microarrays according to manufacturer's instructions (Affymetrix). Fluorescence intensities were quantified using the GCOS 1.2 software. Gene expression data were normalized with RMA algorithm and analyzed using GeneSpring software package (Agilent). Data have been deposited in the NCBI Gene Expression Omnibus (GEO, series accession GSE46873).

To identify the predominant normal tissue expression patterns of the 236 proteins identified in the index DLBCL cell line NAS proteome (B593 cells), gcRMA-normalized Affymetrix gene expression data from 79 normal human tissues were retrieved from the BioGPS portal (http://biogps.org/) (Su et al, 2004). A given factor was scored as showing ‘off-context’ expression in lymphoma, if its normal tissue expression pattern was predominantly non-lymphoid; *i.e*. if the factor in question showed expression of 10-fold above the median in a given non-lymphoid cell or tissue type and below median expression in normal lymphoid tissues or cells. The median was calculated across all 79 normal cell or tissue types (Su et al, 2004).

Additional published normal lymphoid and lymphoma transcriptomic data were as follows; GSE2350 (Basso et al, 2005), GSE10846 (Lenz et al, 2008), GSE4475 (Hummel et al, 2006) and GSE29449 (Mertz et al, 2011). Processed data were retrieved through the GEO portal (http://www.ncbi.nlm.nih.gov/geo/). Transcriptomic data from the MYC-inducible B-cell line, P493 (Schlosser et al, 2005) were kindly provided by Professor Dirk Eick. Following normalization, clustering and seriation, data were visualized as heat-maps using the PermutMatrix software (http://www.lirmm.fr/∼caraux/Permut Matrix/) (Caraux & Pinloche, 2005). The following data treatment options were used: Euclidian distance of dissimilarity, McQuitty's method of hierarchical clustering and multiple fragment heuristic seriation rule.

### GSEA analysis

GSEA is a computational method that determines whether an a priori defined set of genes shows statistically significant, concordant differences between two biological states (Subramanian et al, 2005). Analysis was performed using GSEA v2.07 software against the c2 gene sets from the Broad Institute Molecular Signature Database (http://www.broadinstitute.org/gsea/msigdb/). A detailed description of GSEA methodology and interpretation is provided online (http://www.broadinstitute.org/gsea/doc/GSEAUserGui-deFrame.html). In brief, the normalized enrichment score (NES) provides ‘the degree to which a gene set is overrepresented at the top or bottom of a ranked list of genes’. The false discovery rate *q*-value (FPREFIXDR > *q*-val) is ‘the estimated probability that a gene set with a given NES represents a false positive finding’.

### Immunoblot

Protein lysates were resolved by SDS-PAGE and transferred onto nitrocellulose membranes for blotting with anti-CYCLON, anti-MYC or anti-H3 antibodies. After treatment with blocking solution (1× PBS, 8% skimmed milk, 0.1% Tween 20), membranes were incubated with primary antibody (CYCLON, 0.2 µg/mL, MYC, 0.5 µg/mL, H3 0.1 µg/mL in PBS 3% skimmed milk 0.1% Tween 20), washed and incubated with anti-rabbit or mouse IgG-HRP (Thermo Fisher Scientific) before incubation in ECL SuperSignal West Pico Chemiluminescent Substrate (Thermo Fisher Scientific). Images were captured using a ChemiDoc XRS+ imager system (Biorad) or autoradiography films.

### CD20 surface expression analysis

Membrane CD20 expression was determined by direct fluorescence. Briefly, 5 × 10^5^ cells/mL were incubated for 20 min at 4 °C with FITC-conjugated, anti-CD20 monoclonal antibody (clone 2H7, Ozyme). Immunostained cells were analyzed by flow cytometry (LSRII, BD). Non-specific staining was determined using an FITC-conjugated control IgG (IgG2bκ, clone MG2b-57, Ozyme). Percentages of positive cells and mean fluorescence intensities (MFI) were recorded and analyzed with FCS express software.

### Gene silencing

Non-targeting or CYCLON targeting short hairpin (sh) RNA sequences were designed using the DSIR algorithm (http://biodev.cea.fr/DSIR/DSIR.html) (Vert et al, 2006). Sequences provided in Table S5, were cloned into the pLKO-1 lentiviral vector (Addgene) and packaged, as described (Levy et al, 2010). pLKO-1 expressing MYC targeting shRNA sequences A and B were a gift from Dr Alessio Zippo (Zippo et al, 2007). Cells were transduced with lentiviral particles at an MOI (multiplicity of infection) of 10. Stable cell lines were established under puromycin selection (1 µg/mL). Knockdown efficiency was assessed by western blotting and RT-qPCR.

### Evaluation of Rituximab sensitivity

A total of 5 × 10^5^ cells were treated with Rituximab (Roche) or control IgG (Herceptin, Roche): 24 h with a dose of 10 µg/mL for direct killing assays and 1 h with a dose of 1 µg/mL in presence of 20% human serum as a source of complement, for CDC assays. Cell viability was evaluated by Annexin V/propidium iodide (PI) double staining (Beckman Coulter) or PI staining, followed by flow cytometry analysis (LSRII, BD). Specific cell death was calculated as (% of cell death with Rituximab − % cell death with Herceptin)/(1 − % cell death with Herceptin).

### Xenotransplantation assay

All animal experiments were conducted in agreement with the Principles of Laboratory Animal Care (National Institutes of Health publication no. 86-23, revised 1985) and approved by the regional ethics committee. Raji cells (5.10^6^) were injected subcutaneously into the flanks of 6–8 week-old SCID mice (Charles River), as described (Chao et al, 2010). For follow-up of tumour engraftment and growth, 12 mice each were injected into the right and left flanks, respectively, with either shCtrl or shCYCLON-transduced Raji cells expressing luciferase. For Rituximab treatment and survival analysis, 24 mice were injected into the right flank, with either shCtrl or shCYCLON-transduced Raji cells expressing luciferase. When tumours were palpable (∼0.5 mm^3^), mice were given daily intraperitoneal injections of 200 µg Rituximab or control IgG (Non Swiss Albino Mouse IgG, Innovative research). Tumour volume was measured every 2 days and calculated using the formula *l*^2^ × *L* × 0.52 (*n* = 6 for each group). Bioluminescence imaging was performed weekly, under inhaled anaesthesia (3% isoflurane), administered through a nose cone. Mice then received an intraperitoneal injection of D-luciferin potassium salt dissolved in sterile phosphate-buffered serum (150 mg/kg) 10 min before imaging (ORCAII-BT-512G, Hamamatsu Photonics), as described previously (Jin et al, 2006). Semi-quantitative data were obtained from the bioluminescence images by drawing regions of interest on the area to be quantified. Results were expressed as the number of relative light units (RLU) per pixel per second.

### Cell death assays

Cell death assays were undertaken using JQ1 (dose and time as indicated) and for 24 h with 20 nM etoposide or 1 µg/mL 7C11 antibody to engage FAS receptor death signalling, as described (Lajmanovich et al, 2009). Cell viability was evaluated by AnnexinV/IP (Beckman Coulter) or IP staining and flow cytometry analysis (LSIIR, BD). Specific cell death was calculated as (% of drug-induced cell death − % control cell death)/(1 − % control cell death) as described (Lajmanovich et al, 2009).

### Statistics

Excel® 2010 (Microsoft) and J.M.P.® 10.0.0 (SAS Institute) software were used for graphical representations and to perform statistical analysis across all experiments and for survival analysis. For the latter, CYCLON values at diagnosis were defined as high (above 3rd quartile) or low (below 1st quartile) and Kaplan–Meier survival curves and the log-rank test used to estimate overall survival. Survival analysis in lymphoma xenograft experiments was performed by the Kaplan–Meier method and the *p*-value calculated by log-rank test.

The Wilcoxon non-parametric method or the two-sided Student t test were used for statistical analysis in gene expression and cell death analyses and in *in vivo* tumour growth and Rituximab response assays to assess statistical significance. Histograms represent the average for each group and errors bars represent standard error of the mean.

B-cell non-Hodgkin lymphoma (B-NHL) comprises a complex spectrum of lymphoid cancers that manifest heterogeneous clinical outcomes to standardized therapies tailored to well-defined subtypes. A significant advance in treatment of aggressive B-NHL has been achieved with anti-CD20 monoclonal antibodies (Rituximab) both in combination with chemotherapy or alone as a maintenance treatment. However, a proportion of patients fail to respond to, or more commonly relapse, after receiving Rituximab-containing therapy. The cellular and molecular mechanisms underlying these events are not well characterized and treatment strategies to override treatment resistance events are lacking.

RESULTS:

To discover new factors involved in tumour progression and treatment resistance in aggressive lymphoma, we developed a transcriptome-driven proteomics approach focused on the identification of illegitimately activated factors, termed here ‘off-context’, that are involved in oncogenic signalling and treatment resistance in high risk B-cell lymphoma. Using this strategy, we identified the nuclear factor CYCLON, which has not been previously connected to cancer, as a novel transcriptional regulator of the mature B-cell development program that autonomously drives tumour growth and resistance to monoclonal antibody therapy in B-cell NHL. Strikingly, CYCLON activity can be targeted through BET bromodomain inhibition downstream of MYC.

IMPACT:

This work provides new biological insights into disease progression and treatment resistance in aggressive lymphoid cancers and provides proof of principle that systematic searches for ‘off-context’ gene expression events is a powerful approach for discovering new oncogenic and treatment resistance mechanisms in cancer.

## Author contributions

AE designed study, performed experiments, analyzed data and co-wrote the manuscript. SR designed study, analyzed transcriptomic and public clinical data and co-wrote the manuscript. JBC designed and performed Rituximab sensitivity assays and flow cytometry analyses. CR designed and performed animal studies. SD designed and performed shRNA cloning. SH, PB performed lentiviral transductions and flow cytometry analyses. AD, FC analyzed transcriptomic and public clinical data. DL analyzed data. BB analyzed proteomics data. SKJ performed mass spectrometry analyses. EF performed JQ1 synthesis. CEM designed JQ1 synthesis. CP designed study. CB, JG, MF designed proteomic analyses. RG analyzed clinical data. MBC and SK conceived and designed the study, analyzed data and wrote the manuscript.

## Supporting Information

As a service to our authors and readers, this journal provides supporting information supplied by the authors. Such materials are peer reviewed and may be re-organized for online delivery, but are not copy-edited or typeset. Technical support issues arising from supporting information (other than missing files) should be addressed to the authors.

**Figure S1** Proteomic analysis of a DLBCL nuclear acid soluble (NAS) fraction and identification of nuclear factors which show evidence of abnormal de-repression in lymphoma. **Figure S2.** Association of *H2AFY and CYCLON* levels with clinical outcome in DLBCL. **Figure S3.** Establishment of CYCLON knock-down lymphoma cell lines. **Figure S4.** Differential expression of genes in CYCLON knock-down Raji lymphoma B cells. **Figure S5.** Evaluation of drug sensitivity in CYCLON knock-down B lymphoma cells. **Figure S6.** Characterization of JQ1 final compound. **Figure S7.** Cell death assays upon JQ1 and JQ1/etoposide treatment. **Table S1.** Protein list from LC-MS/MS analysis of B593 NAS fraction. **Table S2.** Genes significantly modulated between Raji shCtrl and Raji shCYCLON cell lines. **Table S3.** GSEA ‘Aggressive multiple myeloma’ signature. **Table S4.** GSEA ‘CD40 dependent B cell’ signature. **Table S5.** Oligonucleotides used for this study.
