# Supplementary material for: Identification of a novel BET bromodomain inhibitor-sensitive, gene regulatory circuit that controls Rituximab response and tumour growth in aggressive lymphoid cancers
Source: EMBO Mol Med. 2013 Jul 4;5(8):1180–95. doi: 10.1002/emmm.201202034 (PMC3944460; doi:10.1002/emmm.201202034)
Supplement: Supplementary file 1 [file emmm0005-1180-SD1.pdf]

SUPPORTING INFORMATION

Table of contents

Supplemental Methods **2**

Supplemental Figures **3**

Figure S1: Proteomic analysis of a DLBCL nuclear acid soluble (NAS) fraction and identification of nuclear factors which show evidence of abnormal de-repression in lymphoma. *3*

Figure S2: Association of *H2AFY and CYCLON* levels with clinical outcome in DLBCL. *4*

Figure S3: Establishment of CYCLON knock-down lymphoma cell lines. *5*

Figure S4: Differential expression of genes in CYCLON knock-down Raji lymphoma B cells. *6*

Figure S5: Evaluation of drug sensitivity in CYCLON knock-down B lymphoma cells. *7*

Figure S6: Characterization of JQ1 final compound. *8*

Figure S7: Cell death assays upon JQ1 and JQ1/etoposide treatment. *9*

Supplemental Tables **10**

Table S1: Protein list from LC-MS/MS analysis of B593 NAS fraction. *10*

Table S2: Genes significantly modulated between Raji shCtrl and Raji shCYCLON cell lines. *12*

Table S3: GSEA "Aggressive multiple myeloma" signature. *32*

Table S4: GSEA "CD40 dependent B cell" signature. *33*

Table S5: Oligonucleotides used for this study. *34*

SUPPLEMENTAL METHODS

Antibody-Dependent Cell-Mediated Cytotoxicity assay

For Antibody-Dependent Cell-Mediated Cytotoxicity assay (ADCC), 10^6 target cells were labelled with 125 µCi ^{51}Cr by incubating for 1 h at 37°C. After washing, labeled cells were resuspended in regular culture medium. PBMC prepared by centrifugation on a Ficoll density gradient from healthy donors' veinous blood were used as effector cells. Briefly, 10^5 target cells were mixed with PBMC at effector:target ratios of 50:1, 25:1 or 12.5:1, with 10 µg/ml of Rituximab or Herceptin (as control isotype). After 4-h incubation inhumidified atmosphere of 95% air and carbon dioxide, the ^{51}Cr-release was determined in the formula 100x(E-S)/(T-S), where E is the cpm released in the presence of effector cells, T is the cpm released after target cells incubation with 1% saponin, and S is the cpm spontaneously released by target cells incubated with medium alone.

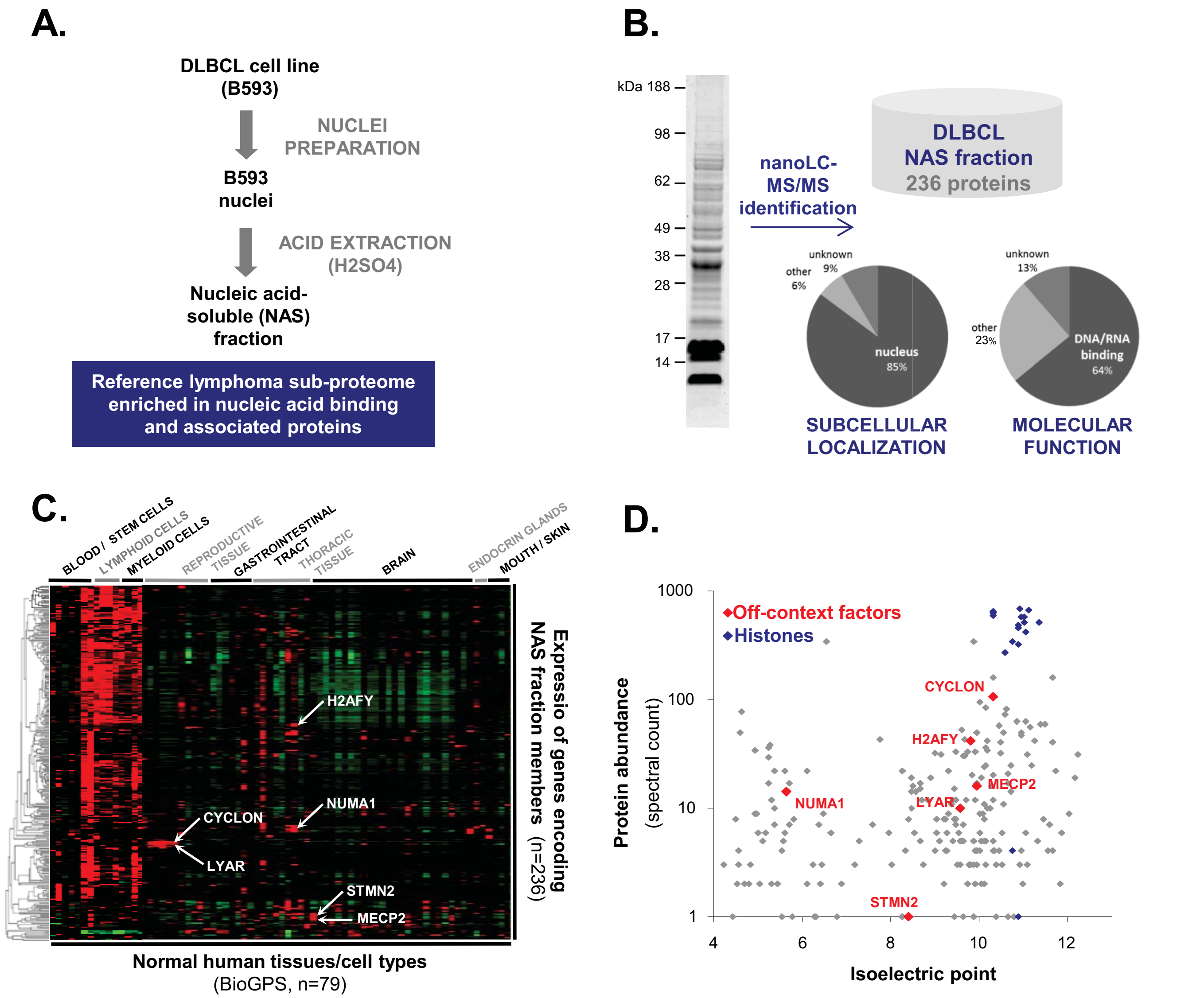

Figure S1 - Proteomic analysis of a DLBCL nuclear acid soluble (NAS) fraction and identification of nuclear factors which show evidence of abnormal de-repression in lymphoma.
A. Experimental strategy for B593 DLBCL cell line NAS fraction extraction, **B.** SDS-PAGE separation of NAS fraction proteins from the DLBCL cell line (left) and number, subcellular localization and molecular function of proteins identified by mass spectrometry from this fraction (right). **C.** Heat-map of physiological gene expression profiles (79 tissues comprising both lymphoid and non-lymphoid) for genes encoding the 236 factors identified by mass-spectrometry in the NAS fraction of B593 DLBCL cells (retrieved from BioGPS portal). “Off-context” factors are indicated (white arrows). **D.** Relative abundance (retrieved from spectral counts) and isoelectric point of all NAS fraction proteins including “off-context” factors (red) and core histones (blue).

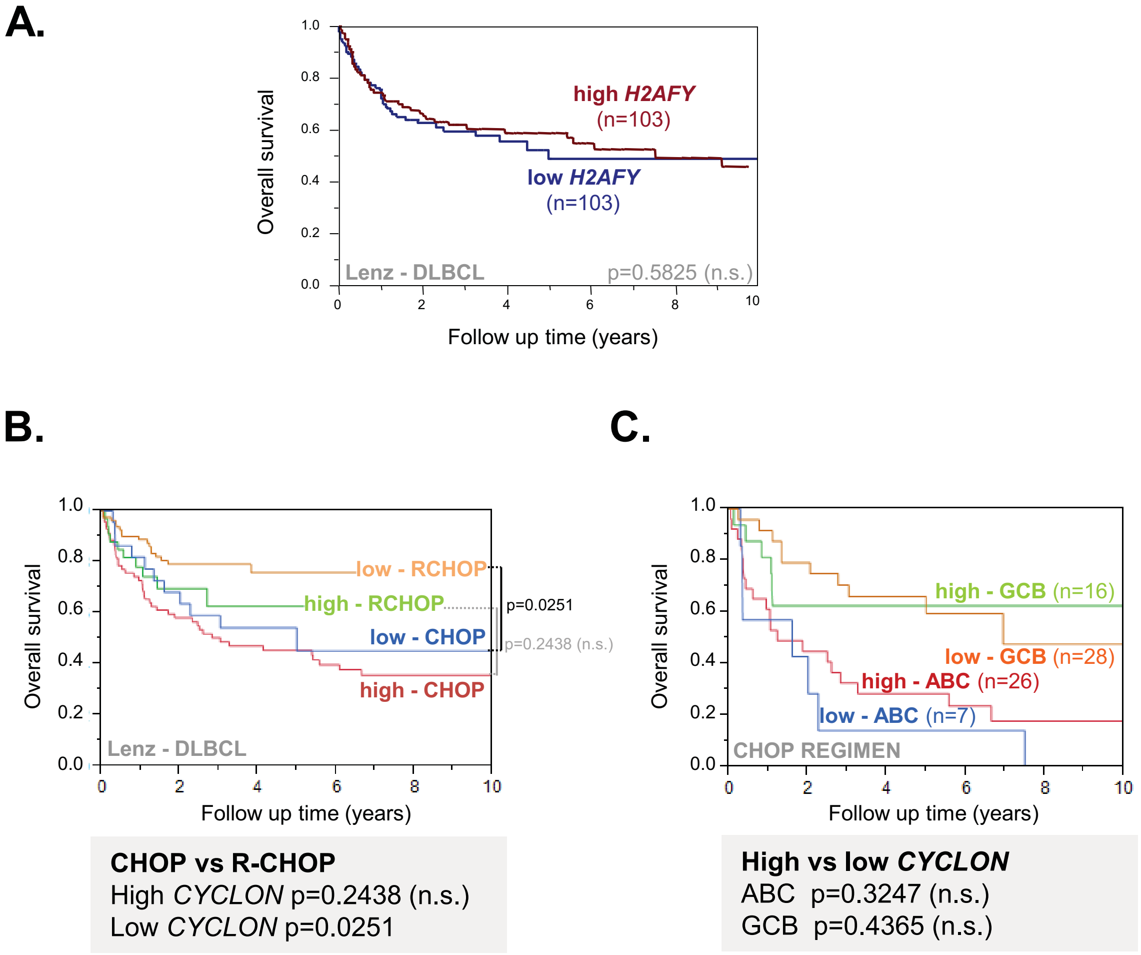

Figure S2 - Association of *H2AFY and CYCLON* levels with clinical outcome in DLBCL.
Kaplan-Meier cumulative survival curves showing predicted survival in DLBCL patients from the Lenz (GEO GSE10846) study, as indicated. **A.** R-CHOP and CHOP-treated patients with high (above 3rd quartile) or low (below 1st quartile) *H2AFY* expression levels. **B.** R-CHOP or CHOP-treated patients with high (above 3rd quartile) or low (below 1st quartile) *CYCLON* expression levels. **C.** CHOP-treated patients with high (above 3rd quartile) or low (below 1st quartile) *CYCLON* expression levels according to DLBCL molecular subtype (ABC or GCB), p value from log-rank test ; n.s. : non significant.

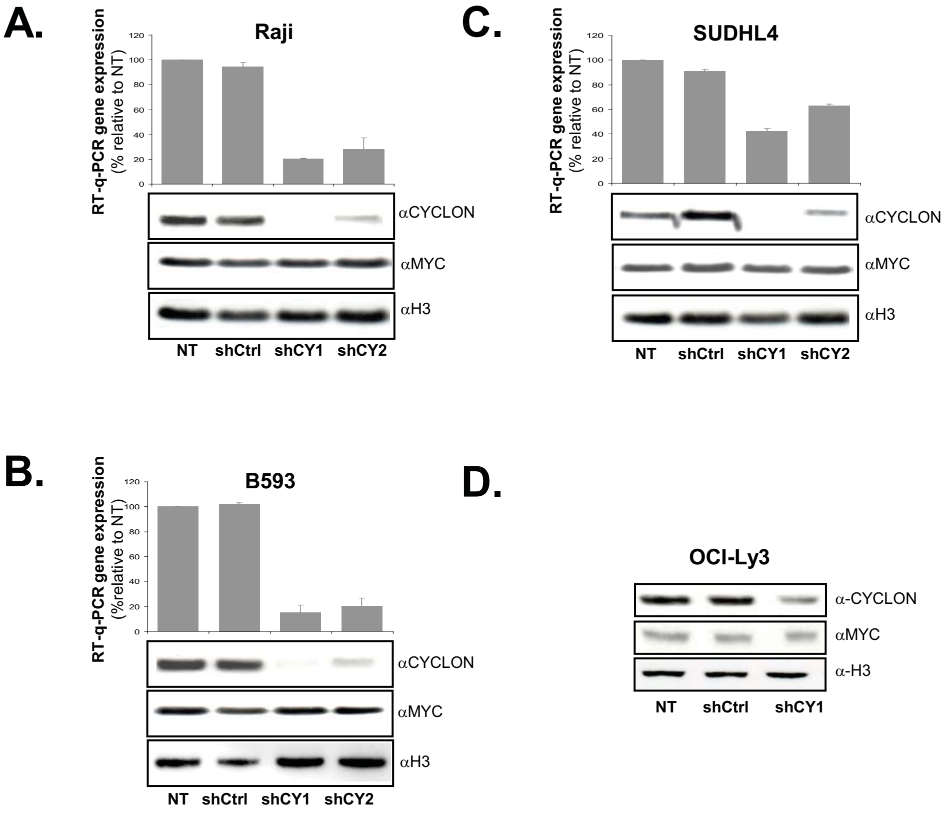

Figure S3 - Establishment of CYCLON knock-down lymphoma cell lines.
Expression analysis by RT-qPCR (*CYCLON*) and western blotting (CYCLON, MYC and histone H3, lower panels), as indicated, in Raji (**A**), B593 (**B**), SUDHL4 (**C**) and OCI-Ly3 (**D**) cell lines non transduced (NT), transduced with a non-targeting shRNA (shCtrl), or transduced with CYCLON-targeting shRNA (shCY1 = shCYCLON and CY2) ; n=3

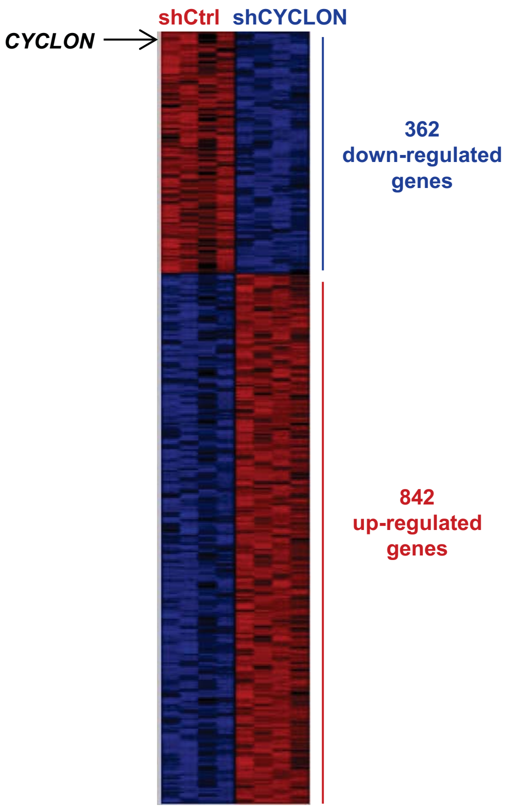

Figure S4 - Differential expression of genes in CYCLON knock-down Raji lymphoma B cells.
Heatmap showing differential expression of genes in shCtrl (n=4 biological replicates) or shCYCLON-treated (n=4 biological replicates) Raji B lymphoma cells (unpaired t-test with Benjamini Hochberg correction, corrected p value <0.05, fold change ≥1.2) ; see also Table S2.

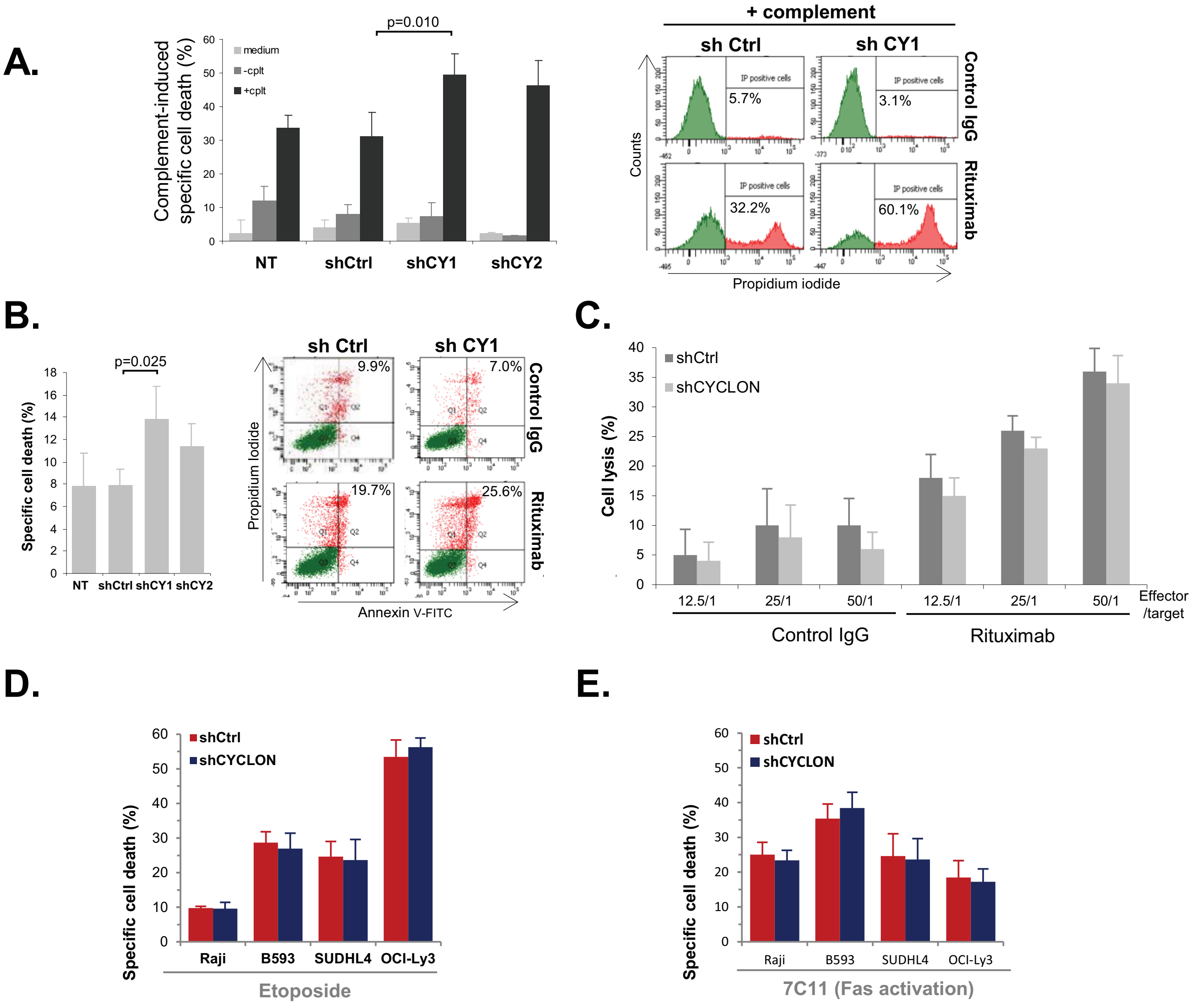

Figure S5 - Evaluation of drug sensitivity in CYCLON knock-down B lymphoma cells.
A-C. Evaluation of Raji sensitivity to Rituximab for non transduced cells (NT), cells transduced with a non-targeting shRNA (shCtrl), or cells transduced with two different CYCLON targeting shRNA (shCY1 and CY2), respectively. **A.** CDC assays after 1 µg/ml Rituximab or control IgG (Herceptin) treatment for 1h, in the presence or absence of human complement. Typical flow cytometry results using propidium iodide staining for shCtrl and shCY1 in Raji cells. **B.** Rituximab-induced apoptosis assays with 10 µg/ml Rituximab or control IgG (Herceptin) for 24h. Typical flow cytometry results using Annexin V / propidium iodide staining for shCtrl and shCY1 in Raji cells, p values are derived from a Wilcoxon test, n=8 for each cell line. **C.** ADCC ^{51}Cr release assay in Raji cells: non transduced (NT), transduced with a non-targeting shRNA (shCtrl), or CYCLON-targeting shRNA (shCYCLON); treated with 10 µg/ml of Rituximab or Herceptin (as a control isotype); and with peripheral blood mononuclear cells (PBMC) at effector:target ratios of 50:1, 25:1 or 12.5:1 for 4h as described in Supplemental Methods section. **D-E.** Evaluation of etoposide (**D**) and Fas-dependant apoptosis (**E**) of Raji, B593, SUDHL4 and OCI-Ly3 cells transduced with a non-targeting shRNA (shCtrl) or with a CYCLON-targeting shRNA (shCYCLON), after a 24h treatment with 20 nM etoposide or DMSO and 1 µg/ml anti-FAS (7C11) or control IgG antibodies ; p values are derived from a Wilcoxon test, n=8 for each cell line.

A.

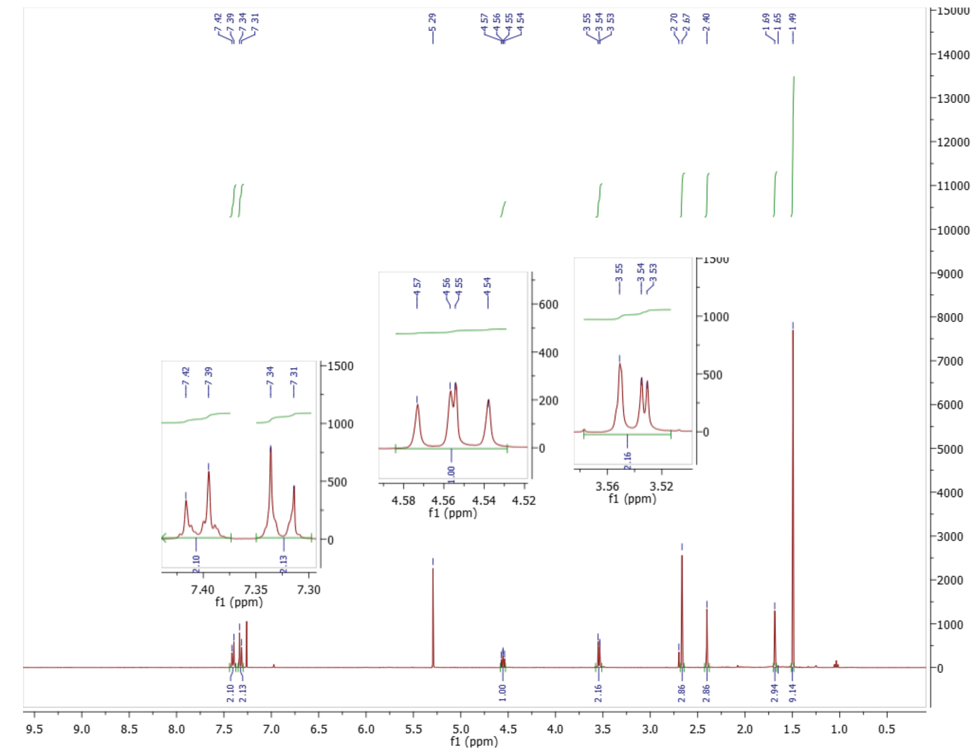

B.

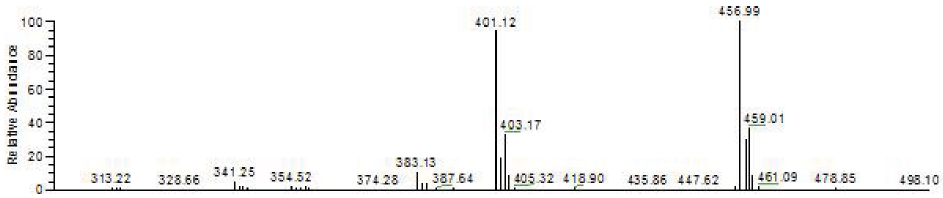

C.

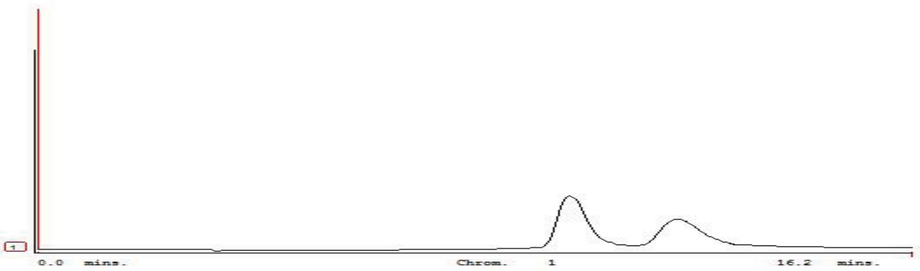

Figure S6 - Characterization of JQ1 final compound

A. ^{1}H NMR spectra (CDCl3, 400MHz, 25°C): δ 7.39 (d, J = 8.5 Hz, 2H), 7.31 (d, J = 8.4 Hz, 2H), 4.55 (dd, J^1 = 6.5 Hz, J^2 = 8 Hz, 1H), 3.54 (dd, J^1 = 6.5 Hz, J^2 = 8 Hz, 2H), 2.67 (s, 3H), 2.40 (s, 3H), 1.69 (s,3H), 1.49 (s, 9H). Consistent with literature values: 1H NMR (600 MHz, CDCl3, 25 °C) δ 7.39 (d, J = 8.4 Hz, 2H), 7.31 (d, J = 8.4 Hz, 2H), 4.54 (t, J = 6.6 MHz, 1H), 3.54-3.52 (m, 2H), 2.66 (s, 3H), 2.39 (s, 3H), 1.67 (s, 3H), 1.48 (s, 9H). **B.** Low Resolution Mass Spectrometry (m/z) of final sample (+ESI). 456.99 [M+H], 401.12 fragmentation peak [M – tBu]. **C.** HPLC trace – analytical chiral column OD-H; *n*-hexane:2-propanol 85:15; (+)-JQ1 r.t.: 10 min, 55%, (-)-JQ1 r.t.: 12 min, 45%.

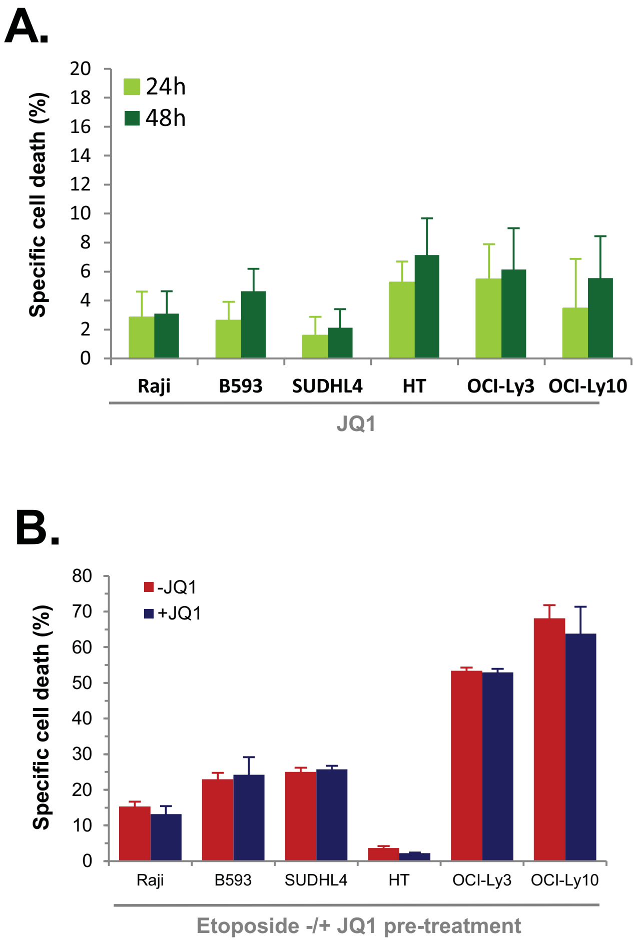

Figure S7 – Cell death assays upon JQ1 and JQ1/etoposide treatment.
A. Evaluation of specific cell death after a 24h or 48h treatment with 1 μM JQ1 or vehicle (DMSO), Specific cell death **=** [JQ1-DMSO)/(100-DMSO)]x100, n=4 for each cell line. **B**. Evaluation of specific cell death after a 24h pre-treatment with 1 μM JQ1 or vehicle (DMSO) followed by a 24h treatment with 20 μM etoposide or vehicle (DMSO), Specific cell death **=** [etoposide-DMSO)/(100-DMSO)]x100 ; n=4 for each cell line.

Table S1: Protein list from LC-MS/MS analysis of B593 NAS fraction

	Description	Uniprot accession	Uniprot primary accession	Gene name	Mascot score	Mass (kDa)	Protein coverage (%)	Peptides number (distinct)	Peptide number (redundant) = spectral count	pI	Molecular fonction[1]	Biological process[1]	Subcellular localisation[1]	Alternative localisation[1]
1	(P16401) Histone H1.5 (Histone H1a)	H15_HUMAN	P16401	HIST1H1B,H1F5	1720.472416	22435	67.49	33	692	10.91	DNA binding	chromatin organization	nucleus	
2	(P68431) Histone H3.1 (H3/a) (H3/b) (H3/c) (H3/d) (	H31_HUMAN	P68431	HIST1H3A,H3FA,HIST1H3B,H3F	1089.92417	15263	63.77	26	668	11.13	DNA binding	chromatin organization	nucleus	
3	(P62807) Histone H2B.a/g/h/k/l (H2B.1 A) (H2B/a) (I	H2B1C_HUMAN	P62807	HIST1H2BC,H2BFL,HIST1H2BE	769.7848129	13767	80.00	19	653	10.32	DNA binding	chromatin organization	nucleus	
4	(O60814) Histone H2B K (HIRA-interacting protein 1	H2B1K_HUMAN	O60814	HIST1H2BK,H2BFT, HIRIP1	808.6248129	13751	81.60	19	650	10.32	DNA binding	chromatin organization	nucleus	
5	(P33778) Histone H2B.f (H2B/f) (H2B.1)	H2B1B_HUMAN	P46778	HIST1H2BB,H2BFF	766.607904	13811	73.60	16	628	10.32	DNA binding	chromatin organization	nucleus	
6	(P06899) Histone H2B.r (H2B/r) (H2B.1)	H2B1J_HUMAN	P06899	HIST1H2BJ,H2BFR	812.9548129	13765	80.00	18	628	10.32	DNA binding	chromatin organization	nucleus	
7	(Q99880) Histone H2B.c (H2B/c)	H2B1L_HUMAN	Q99880	HIST1H2BL,H2BFC	813.2348129	13813	81.60	19	592	10.32	DNA binding	chromatin organization	nucleus	
8	(P16403) Histone H1.2 (Histone H1d)	H12_HUMAN	P16403	HIST1H1C,H1F2	1301.288741	21221	64.06	29	572	10.94	DNA binding	chromatin organization	nucleus	
9	(P10412) Histone H1.4 (Histone H1b)	H14_HUMAN	P10412	HIST1H1E,H1F4	1422.480694	21721	60.91	31	572	11.03	DNA binding	chromatin organization	nucleus	
10	(P16402) Histone H1.3 (Histone H1c)	H13_HUMAN	P16402	HIST1H1D,H1F3	1267.520599	22205	60.19	30	517	11.02	DNA binding	chromatin organization	nucleus	
11	(P62805) Histone H4	H4_HUMAN	P62805	HIST1H4A,H4/A, H4FA,HIST1H4	1292.155271	11229	76.47	23	514	11.36	DNA binding	chromatin organization	nucleus	
12	(P0C0S8) Histone H2A type 1 (H2A.1)	H2A1_HUMAN	P0C0S8	HIST1H2AG,H2AFP,HIST1H2AI,	487.1748129	13952	52.38	11	485	10.9	DNA binding	chromatin organization	nucleus	
13	(P20671) Histone H2A type 1-D (H2A.3)	H2A1D_HUMAN	P07910	HIST1H2AD,H2AFG,	510.8348129	13968	52.38	12	453	10.9	DNA binding	chromatin organization	nucleus	
14	(P28001) Histone H2A type 1-E (H2A.2) (H2A/m)	H2A1E_HUMAN	P04908	HIST1H2AE,H2AFA,	515.1648129	13996	51.97	11	416	11.05	DNA binding	chromatin organization	nucleus	
15	(Q3MIH3) Ubiquitin and ribosomal protein L40, (Ubic	Q3MIH3_HUMAN	P0CG47	UBA52,hCG_37048,	264.6	14719	47.37	14	340	9.87	stuctural component of ribosome	protein biosynthesis	nucleus	cytoplam
16	(P16104) Histone H2A.x (H2a/x)	H2AX_HUMAN	P16104	H2AFX,H2AX,	288.3338177	15004	22.54	10	336	10.74	DNA binding	chromatin organization	nucleus	
17	(P62988) Ubiquitin	UBIQ_HUMAN	P62979	RPS27A,UBA80, UBCEP1,UBA5	722.8069089	8560	87.01	13	336	6.56	other, protein binding	protein targeting and degradation; signal transductio	cytoplasm	nucleus
18	(Q8IUE6) Histone H2A type 2-B	H2A2B_HUMAN	Q8IUE6	HIST2H2AB	290.147904	13856	35.43	9	320	10.88	DNA binding	chromatin organization	nucleus	
19	(Q71UI9) Histone H2AV (H2A.F/Z)	H2AV_HUMAN	Q71UI9	H2AFV,H2AV,	283.6307266	13369	37.19	5	272	10.58	DNA binding	chromatin organization	nucleus	
20	(P17096) High mobility group protein HMG-I/HMG-Y	HMGA1_HUMAN	P17096	HMGA1,HMGIY,	924.8134436	11538	73.58	18	159	10.31	DNA binding	chromatin organization	nucleus	
21	(P26373) 60S ribosomal protein L13 (Breast basic (	RL13_HUMAN	P26373	BBC1,OK/SW-cl.46,	1086.065808	24115	58.90	24	158	11.65	stuctural component of ribosome	protein biosynthesis	cytoplasm	nucleus
22	(Q9H6F5) Hypothetical protein FLJ22321 (Hypothet	CCD86_HUMAN	Q9H6F5	CCDC86,CYCLON,	1405.406729	40211	62.74	28	106	10.33	unknown	unknown	nucleus	
23	(P05204) Nonhistone chromosomal protein HMG-17	HMGN2_HUMAN	P05204	HMGN2,HMG17,	1148.86	9256	85.39	21	94	10	DNA binding	chromatin organization ; transcription (+)	nucleus	
24	(O76021) Ribosomal L1 domain-containing protein 1	RL1D1_HUMAN	O76021	CATX11, CSIG, PBK1,L12,	1509.316197	54939	44.89	31	83	10.13	stuctural component of ribosome	protein biosynthesis	cytoplasm	nucleus
25	(P62424) 60S ribosomal protein L7a (Surfeit locus p	RL7A_HUMAN	P62424	RPL7A,SURF-3, SURF3,	913.1176355	29846	46.86	23	81	10.61	stuctural component of ribosome	protein biosynthesis	cytoplasm	nucleus
26	(P06748) Nucleophosmin (NPM) (Nucleolar phosphc	NPM_HUMAN	P06748	NPM1,NPM,	732.2565347	32555	36.61	19	78	4.64	other, protein binding	chromatin organization ; apoptosis	nucleus	
27	(Q02878) 60S ribosomal protein L6 (TAX-responsiv	RL6_HUMAN	Q02878	RPL6,TXREB1,	1189.423943	32577	56.08	29	72	10.59	stuctural component of ribosome	protein biosynthesis	cytoplasm	nucleus
28	(P62917) 60S ribosomal protein L8	RL8_HUMAN	P62917	RPL8	827.82	27876	36.33	17	63	11.04	stuctural component of ribosome	protein biosynthesis	cytoplasm	nucleus
29	(P62753) 40S ribosomal protein S6 (Phosphoprotei	RS6_HUMAN	P62753	RPS6,OK/SW-cl.2,	989.9676355	28663	43.08	24	63	10.85	stuctural component of ribosome	protein biosynthesis ; cell cycle	cytoplasm	nucleus
30	(P0CG48) Polyubiquitin-C	Q53G20_HUMAN	P0CG48	UBC	582.1067877	23630	37.38	14	60	11.4	stuctural component of ribosome	protein biosynthesis	cytoplasm	nucleus
31	(P84098) 60S ribosomal protein L19	RL19_HUMAN	P62750	RPL19	466.6138177	23451	30.05	12	59	11.48	stuctural component of ribosome	protein biosynthesis	cytoplasm	nucleus
32	(P62750) 60S ribosomal protein L23a	RL23A_HUMAN	P09429	RPL23A	629.4738177	17684	40.63	12	58	10.44	stuctural component of ribosome	protein biosynthesis	cytoplasm	nucleus
33	(Q86V81) THO complex subunit 4 (Tho4) (Ally of AM	THOC4_HUMAN	Q86V81	THOC4,ALY, BEF,	470.987904	26741	25.40	10	56	11.15	DNA binding	transcription	nucleus	
34	(P05114) Nonhistone chromosomal protein HMG-14	HMGN1_HUMAN	P05114	HMGN1,HMG14,	1108.924439	10522	82.83	18	52	9.61	DNA binding	chromatin organization	nucleus	
35	(Q8IXL5) Hypothetical protein HSPC111 (HBV pre-S	Q8IXL5_HUMAN	P50914	CGI-117, HSPC111	679.3107266	21175	52.08	13	50	9.94	unknown	unknown	nucleus	
36	(P50914) 60S ribosomal protein L14 (CAG-ISL 7)	RL14_HUMAN	P83731	RPL14	176.0169089	23144	20.48	13	50	10.94	stuctural component of ribosome	protein biosynthesis	cytoplasm	nucleus
37	(P19338) Nucleolin (Protein C23)	NUCL_HUMAN	Q15233	NCL	592.8361082	76437	16.57	15	50	4.6	DNA/RNA/protein binding	chromatin organization	nucleus	
38	(P61254) 60S ribosomal protein L26	RL26_HUMAN	P61254	RPL26	720.040463	17248	43.59	16	49	10.55	stuctural component of ribosome	protein biosynthesis	cytoplasm	nucleus
39	(Q6PK04) MGC16597 protein	Q6PK04_HUMAN	Q6PK04	CCDC137,MGC16597	850.3399783	33211	51.83	21	47	10.94	unknown	unknown	unknown	
40	(Q5SSJ5) HP1-BP74	Q5SSJ5_HUMAN	Q5SSJ5	HP1BP3,RP5-930J4.3-001, hC(	876.415276	61169	27.34	20	43	9.69	DNA binding	chromatin organization	nucleus	
41	(P26583) High mobility group protein B2 (High mobil	HMGB2_HUMAN	P26583	HMGB2,HMG2,	997.0805731	23888	63.59	23	43	7.77	DNA binding	chromatin organization ; DNA repair	nucleus	
42	(P83731) 60S ribosomal protein L24 (Ribosomal prc	RL24_HUMAN	P18124	RPL24	542.3086307	17768	45.34	13	42	11.26	stuctural component of ribosome	protein biosynthesis	cytoplasm	nucleus
43	(P22087) Fibrillarin (34 kDa nucleolar scleroderma a	FBRL_HUMAN	P60709	FBL, FIB1, FLRN	856.3209952	33763	49.37	15	42	10.18	RNA binding	ribosome biogenesis	nucleus	
44	(O75367) Core histone macro-H2A.1 (Histone macr	H2AY_HUMAN	P22087	H2AFY,MACROH2A1,	1068.89089	39461	36.03	15	42	9.8	DNA binding	chromatin organization	nucleus	
45	(P47914) 60S ribosomal protein L29 (Cell surface h	RL29_HUMAN	P47914	RPL29	438.7	17610	38.13	10	41	11.66	stuctural component of ribosome	protein biosynthesis	cytoplasm	nucleus
46	(P46778) 60S ribosomal protein L21	RL21_HUMAN	Q9BXS6	RPL21	682.2807266	18422	53.89	15	41	10.49	stuctural component of ribosome	protein biosynthesis	cytoplasm	nucleus
47	(P60709) Actin, cytoplasmic 1 (Beta-actin)	ACTB_HUMAN	Q9Y3A2	ACTB	900.0676355	41710	36.68	15	38	5.29	other, structural protein	cell structure	cytoplasm	nucleus
48	(P62701) 40S ribosomal protein S4, X isoform (Sing	RS4X_HUMAN	P62987	RPS4X,CCG2, RPS4, SCAR,	581.9807266	29448	36.70	13	36	10.16	stuctural component of ribosome	protein biosynthesis ; cell cycle	cytoplasm	nucleus
49	(P10809) 60 kDa heat shock protein, mitochondrial	CH60_HUMAN	P10809	HSPD1,HSP60,	972.9414532	61016	27.44	20	36	5.24	other, protein binding	protein folding	cytoplasm, mitochondria	nucleus
50	(P18621) 60S ribosomal protein L17 (L23)	RL17_HUMAN	P20671	RPL17	454.88	21252	36.79	11	34	10.18	stuctural component of ribosome	protein biosynthesis	cytoplasm	nucleus
51	(P07910) Heterogeneous nuclear ribonucleoproteins	HNRPC_HUMAN	P11142	HNRNPC,HNRPC,	637.3707266	33650	40.66	12	34	4.95	RNA binding	RNA processing	nucleus	
52	(Q9BXS6) Liver nuclear protein	NUSAP_HUMAN	P19338	NUSAP1,ANKT,BM-037, PRO03	744.8186307	49422	36.97	15	33	9.92	DNA binding	cell proliferation, cell cycle (+)	nucleus	
53	(Q5VV70) Nucleolar and coiled-body phosphoprotei	Q5VV70_HUMAN	P33778	NOLC1,RP11-302K17.5-001, h(	692.8032856	73560	19.16	16	33	9.46	RNA binding	RNA processing	nucleus	
54	(P61247) 40S ribosomal protein S3a	RS3A_HUMAN	P61247	RPS3A,MFTL,	674.6062711	29795	49.63	16	33	9.75	stuctural component of ribosome	protein biosynthesis	cytoplasm	nucleus
55	(O00479) High-mobility group nucleosome-binding d	HMGN4_HUMAN	O00479	HMGN4,HMG17L3, NHC,	168.46	9402	30.34	6	32	10.48	DNA binding	chromatin organization ; transcription (+)	nucleus	
56	(Q15233) Non-POU domain-containing octamer-binc	NONO_HUMAN	O75367	NONO, NRB54	122.8669089	54197	6.71	15	32	9.01	DNA/RNA/protein binding	transcription ; recombinaison ; RNA splicing	nucleus	
57	(Q9Y3Y2) Protein C1orf77	CA077_HUMAN	Q9Y3Y2	C1orf77,HT031, PP7704,	390.8369089	26380	29.29	8	31	12.24	unknown	unknown	unknown	
58	(O75475) PC4 and SFRS1-interacting protein (Lens	PSIP1_HUMAN	Q9Y3C1	PSIP1,DFS70, LEDGF, PSIP2,	468.380463	60067	20.33	13	31	9.15	DNA binding	transcription	nucleus	
59	(P36578) 60S ribosomal protein L4 (L1)	RL4_HUMAN	P36578	RPL4,RPL1,	642.3988992	47536	25.46	17	31	11.07	stuctural component of ribosome	protein biosynthesis	cytoplasm	nucleus
60	(Q00059) Transcription factor A, mitochondrial prec	TFAM_HUMAN	Q00059	TFAM,TCF6L2,	311.8138177	29078	20.83	7	30	9.65	DNA binding	transcription	mitochondria	nucleus
61	(P23396) 40S ribosomal protein S3	RS3_HUMAN	P23396	RPS3,OK/SW-cl.26,	846.4969089	26671	61.32	16	30	9.68	stuctural component of ribosome	protein biosynthesis	cytoplasm	nucleus
62	(P62736) Actin, aortic smooth muscle (Alpha-actin-2	ACTA_HUMAN	P62736	ACTSA, ACTVS,GIG46,	236.73	41982	10.50	11	29	5.24	other, structural protein	cell structure	cytoplasm	nucleus
63	(P14678) Small nuclear ribonucleoprotein-associate	RSMB_HUMAN	P14678	SNRPB,COD, SNRPB1,	329.11	24594	34.56	7	25	11.2	RNA binding	RNA processing	nucleus	
64	(Q02543) 60S ribosomal protein L18a	RL18A_HUMAN	Q02543	RPL18A	274.54	20749	23.94	9	25	10.72	stuctural component of ribosome	protein biosynthesis	cytoplasm	nucleus
65	(Q9Y3A2) Probable U3 small nucleolar RNA-associa	UTP11_HUMAN	Q3MIH3	UTP11L,CGI-94, HDCMB12P,	369.8729855	30428	26.81	14	25	10.16	RNA binding	RNA processing	nucleus	cytoplasm
66	(Q6IB29) EBNA1BP2 protein (EBNA1 binding protei	Q6IB29_HUMAN	Q6IB29	EBNA1BP2,RP5-1034F7.2-001,	411.967904	34830	25.95	10	24	10.1	RNA binding	ribosome biogenesis	nucleus	
67	(Q06830) Peroxiredoxin-1 (EC 1.11.1.15) (Thioredo	PRDX1_HUMAN	Q06830	PRDX1,PAGA, PAGB, TDPX2,	174.28	22096	22.50	6	22	8.27	other, metabolic enzyme, deshydrogenase	cell metabolism	cytoplasm	nucleus
68	(Q07020) 60S ribosomal protein L18	RL18_HUMAN	Q07020	RPL18	344.8709952	21490	30.26	8	22	11.73	stuctural component of ribosome	protein biosynthesis	cytoplasm	nucleus
69	(Q9ULW0) Targeting protein for Xklp2 (Restricted e	TPX2_HUMAN	Q9ULW0	TPX2,C20orf1, C20orf2, DIL2, H	287.6691993	85600	10.80	8	22	9.29	other, protein binding	cell structure, cell cycle	nucleus	
70	(P82979) Nuclear protein Hcc-1 (Proliferation-assoc	HCC1_HUMAN	P82979	HCC1,HSPC316,	580.7229855	23525	47.42	11	22	6.12	DNA binding	transcription, cell cycle (+)	nucleus	
71	(P11142) Heat shock cognate 71 kDa protein (Heat	HSP7C_HUMAN	P84098	HSPA8,HSC70, HSP73, HSPA1(	652.1348129	70854	19.10	12	22	5.37	other, protein binding	protein folding	cytoplasm	nucleus
72	(P09429) High mobility group protein B1 (High mobil	HMGB1_HUMAN	O75475	HMGB1,HMG1,	561.2042808	24747	36.16	13	22	5.62	DNA binding	chromatin organization ; apoptosis (-)	nucleus	
73	(P18124) 60S ribosomal protein L7	RL7_HUMAN	P62701	RPL7	419.0807266	29207	36.23	13	21	10.66	stuctural component of ribosome	protein biosynthesis	cytoplasm	nucleus
74	(P62241) 40S ribosomal protein S8	RS8_HUMAN	P62241	RPS8,OK/SW-cl.83,	313.7909952	24059	27.06	7	20	10.32	stuctural component of ribosome	protein biosynthesis	cytoplasm	nucleus
75	(P09012) U1 small nuclear ribonucleoprotein A (U1 s	SNRPA_HUMAN	P09012	SNRPA	213.3753816	31128	22.70	10	20	9.83	RNA binding	RNA processing	nucleus	
76	(P62861) 40S ribosomal protein S30	RS30_HUMAN	P62861	FAU	149.4048129	6644	33.33	4	19	12.15	stuctural component of ribosome	protein biosynthesis ; apoptosis (-)	cytoplasm	nucleus
77	(P38159) Heterogeneous nuclear ribonucleoprotein	HNRPG_HUMAN	P38159	RBMX,HNRPG, RBMXP1,	199.2119903	42306	13.80	5	19	10.06	RNA binding	RNA processing	nucleus	
78	(P37802) Transgelin-2 (SM22-alpha homolog)	TAGL2_HUMAN	P37802	TAGLN2,KIAA0120,CDABP0035	327.9469089	22246	33.66	7	19	8.45	unknown	cell structure	cytoplasm	nucleus, plasma membrane
79	(P61604) 10 kDa heat shock protein, mitochondrial	CH10_HUMAN	P61604	HSPE1	464.9209952	10794	61.22	9	19	8.91	other, protein binding	protein folding	cytoplasm	mitochondria
80	(Q13185) Chromobox protein homolog 3 (Heterochr	CBX3_HUMAN	Q13185	CBX3	296.3869089	20810	20.11	7	17	5.23	other, protein binding	chromatin organization ; transcription (-)	nucleus	cytoplasm
81	(P46781) 40S ribosomal protein S9	RS9_HUMAN	P46781	RPS9	254.9469089	22447	29.90	7	17	10.66	stuctural component of ribosome	protein biosynthesis	cytoplasm	nucleus
82	(P07195) L-lactate dehydrogenase B chain (EC 1.1	LDHB_HUMAN	P07195	LDHB	481.44	36484	26.59	8	17	5.72	other, metabolic enzyme, deshydrogenase	cell metabolism	cytoplasm	
83	(Q8NC51) Plasminogen activator inhibitor 1 RNA-bir	PAIRB_HUMAN	Q8NC51	PAIRBP1,CGI-55,	443.0238177	44938	21.81	10	17	8.66	RNA/protein binding	translation	nucleus	
84	(Q6QHH9) Methyl CpG binding protein 2 isoform B	Q6QHH9_HUMAN	P30050	CCDC137	494.5975299	53291	24.17	11	17	9.95	DNA binding	transcription (-)	nucleus	
85	(P51608) Methyl-CpG-binding protein 2 (MeCP-2 pr	MECP2_HUMAN	P51608	MECP2	365.7617218	52409	15.13	10	16	9.95	DNA binding	transcription (-)	nucleus	
86	(O15347) High mobility group protein B3 (High mobil	HMGB3_HUMAN	O15347	HMGB3,HMG2A, HMG4,	328.267904	22834	27.54	8	16	8.5	DNA binding	chromatin organization ; B cell differentiation (-)	nucleus	
87	(O95478) TGF-beta-inducible nuclear protein 1 (Hai	TIP1_HUMAN	O95478	TINP1,HUSSY-29,	304.7988992	30047	24.18	9	16	10.28	unknown	unknown	nucleus	
88	(O15446) RNA polymerase I-associated factor PAF	PAF49_HUMAN	O15446	CD3EAP,ASE1, CAST, PAF49,	254.3439807	54951	17.03	7	15	8.66	other, protein binding	transcription ; ribosme biogenesis	nucleus	
89	(P23246) Splicing factor, proline- and glutamine-rich	SFPQ_HUMAN	P23246	SFPQ,PSF,	259.7109952	76102	11.11	8	15	9.45	RNA binding	RNA processing, transcription (-), DNA repair	nucleus	
90	(Q14980) Nuclear mitotic apparatus protein 1 (NuMA	NUMA1_HUMAN	Q14980	NUMA1,NUMA,	450.1716162	238130	5.27	10	14	5.63	other, protein binding	cell cycle ; cell structure	nucleus	
91	(P62857) 40S ribosomal protein S28	RS28_HUMAN	P62857	RPS28	233.4009952	7836	57.75	5	14	10.7	stuctural component of ribosome	protein biosynthesis	cytoplasm	nucleus
92	(P04406) Glyceraldehyde-3-phosphate dehydrogen:	G3P_HUMAN	P04406	GAPD,CDABP0047, OK/SW-cl.1	297.67	35899	26.99	7	14	8.58	other, metabolic enzyme, deshydrogenase	cell metabolism	cytoplasm	nucleus
93	(P00338) L-lactate dehydrogenase A chain (EC 1.1	LDHA_HUMAN	P00338	LDHA,PIG19,	327.75	36534	18.37	7	14	8.46	other, metabolic enzyme, deshydrogenase	cell metabolism	cytoplasm	
94	(P61956) Small ubiquitin-related modifier 2 precursc	SUMO2_HUMAN	P61956	SUMO2,SMT3B, SMT3H2,	138.2609952	10864	28.57	3	12	5.32	other, protein binding	protein targeting and degradation; signal transductio	nucleus	
95	(P08579) U2 small nuclear ribonucleoprotein B''	RU2B_HUMAN	P08579	SNRPB2	159.68	25470	14.72	5	12	9.72	RNA binding	RNA processing	nucleus	
96	(P35659) Protein DEK	DEK_HUMAN	P35659	DEK	301.1722904	42648	14.99	7	12	8.69	DNA/histone binding	chromatin organization ; transcription (-)	nucleus	
97	(P56182) NNP-1 protein (Novel nuclear protein 1) (N	NNP1_HUMAN	P56182	RRP1, D21S2056E, NNP1, NOF	346.097904	52807	16.04	7	12	9.39	RNA binding	ribosome biogenesis	nucleus	
98	(Q14978) Nucleolar phosphoprotein p130 (Nucleola	NOLC1_HUMAN	Q14978	NOLC1,KIAA0035,	522.5022904	73677	16.74	7	12	9.46	RNA binding	ribosome biogenesis	nucleus	
99	(Q6P6B4) Hypothetical protein C1orf131	Q6P6B4_HUMAN	Q8NDD1	C1orf131,DKFZp547B1713	333.5569089	32576	18.92	7	12	9.92	unknown	unknown	unknown	
100	(Q13442) 28 kDa heat- and acid-stable phosphopro	HAP28_HUMAN	Q13442	PDAP1,HASPP28,	489.6313574	20618	47.59	9	12	8.84	other, protein binding	signal transduction, cell cycle	cytoplam	nucleus
101	(P62280) 40S ribosomal protein S11	RS11_HUMAN	P62280	RPS11	397.2917218	18419	38.32	9	12	10.31	stuctural component of ribosome	protein biosynthesis	cytoplasm	nucleus
102	(P30050) 60S ribosomal protein L12	RL12_HUMAN	P18621	RPL12	529.8609952	17808	56.52	11	12	9.48	stuctural component of ribosome	protein biosynthesis	cytoplasm	nucleus
103	(P62899) 60S ribosomal protein L31	RL31_HUMAN	P62899	RPL31	143.47	14454	21.37	3	11	10.54	stuctural component of ribosome	protein biosynthesis	cytoplasm	nucleus
104	(P62851) 40S ribosomal protein S25	RS25_HUMAN	P62851	RPS25	128.5	13734	20.97	3	11	10.12	stuctural component of ribosome	protein biosynthesis	cytoplasm	nucleus
105	(O00422) Histone deacetylase complex subunit SAF	SAP18_HUMAN	O00422	SAP18,GIG38,	397.89	17550	37.11	8	11	9.38	other, protein binding	chromatin organization	nucleus	
106	(Q9NX58) Cell growth-regulating nucleolar protein	LYAR_HUMAN	Q9NX58	LYAR,PNAS-5,	101.3509952	43588	7.32	4	10	9.57	other, protein binding	cell cycle	nucleus	plasma membrane
107	(Q9NX24) H/ACA ribonucleoprotein complex subunit	NOLA2_HUMAN	Q9NX24	NHP2,HSPC286,	181.96	17190	16.67	3	10	8.48	RNA binding	ribosome biogenesis	nucleus	
108	(Q96EU6) Hypothetical protein C6orf153 (OTTHUMI	Q96EU6_HUMAN	Q96EU6	C6orf153,HSPC253,	249.6007266	29805	20.00	6	10	10.17	unknown	unknown	unknown	
109	(P15880) 40S ribosomal protein S2 (S4) (LLRep3 p	RS2_HUMAN	P15880	RPS2,RPS4,	225.8369089	31305	14.44	6	10	10.25	stuctural component of ribosome	protein biosynthesis	cytoplasm	nucleus
110	(P61978) Heterogeneous nuclear ribonucleoprotein	HNRPK_HUMAN	P61978	HNRPK,HNRNPK,	296.11	50944	14.90	7	10	5.39	RNA binding	RNA processing ; signal transduction	nucleus	cytoplasm
111	(P62081) 40S ribosomal protein S7	RS7_HUMAN	P62081	RPS7	69.75099517	22113	11.44	4	9	10.09	stuctural component of ribosome	protein biosynthesis	cytoplasm	nucleus
112	(Q9NR30) Nucleolar RNA helicase 2 (EC 3.6.1.-) (N	DDX21_HUMAN	Q9NR30	DDX21	178.652717	87290	7.19	5	9	9.32	RNA binding ; helicase	translation	nucleus	
113	(P01871) Ig mu chain C region	MUC_HUMAN	P01871	IGHM	212.42	49526	11.11	5	9	6.47	other, protein binding	cell metabolism	secreted	
114	(Q9Y2S6) Coiled-coil domain-containing protein 72	CCD72_HUMAN	Q9Y2S6	CCDC72,HSPC016, HSPC330,	209.58	7062	50.00	6	9	10	unknown	unknown	unknown	
115	(P08238) Heat shock protein HSP 90-beta (HSP 84	HS90B_HUMAN	P08238	HSP90AB1,HSP90B, HSPC2, H	258.9015638	83081	8.21	6	9	4.97	other, protein binding	protein folding	cytoplasm	mitochondria
116	(P07900) Heat shock protein HSP 90-alpha (HSP 8(	HS90A_HUMAN	P07900	HSP90AA1,HSP90A, HSPC1, H	275.0715638	84476	7.69	7	9	4.94	other, protein binding	protein folding	cytoplasm	nucleus
117	(P00558) Phosphoglycerate kinase 1 (EC 2.7.2.3) (	PGK1_HUMAN	P00558	PGKA,MIG10, OK/SW-cl.110,	199.63	44455	10.82	7	9	8.3	other, metabolic enzyme, deshydrogenase	cell metabolism	cytoplasm	
118	(P46779) 60S ribosomal protein L28	RL28_HUMAN	P46779	RPL28	379.4007266	15607	45.39	9	9	12.02	stuctural component of ribosome	protein biosynthesis	cytoplasm	nucleus
119	(P63173) 60S ribosomal protein L38	RL38_HUMAN	P63173	RPL38	145.64	8082	16.44	3	8	10.1	stuctural component of ribosome	protein biosynthesis	cytoplasm	nucleus
120	(Q15717) ELAV-like protein 1 (Hu-antigen R) (HuR)	ELAV1_HUMAN	Q15717	ELAVL1,HUR,	203.11	36069	11.62	4	8	9.23	RNA binding	translation	nucleus	cyoplasm
121	(P17066) Heat shock 70 kDa protein 6 (Heat shock	HSP76_HUMAN	P17066	HSPA6,HSP70B',	191.4009952	70984	6.51	4	8	5.81	other, protein binding	protein folding	cytoplasm	nucleus
122	(Q9BRP8) Within bgcn homolog	Q9BRP8_HUMAN	Q9BRP8	WIBG,PYM,	278.6109952	22642	23.41	4	8	9.45	unknown	unknown	nucleus	
123	(O60832) H/ACA ribonucleoprotein complex subunit	DKC1_HUMAN	O60832	DKC1, NOLA4	137.0909952	57507	9.00	5	8	9.46	RNA binding	ribosome biogenesis ; telomerase ;	nucleus	
124	(O75937) DnaJ homolog subfamily C member 8 (Sp	DNJC8_HUMAN	O75937	SPF31,HSPC315, HSPC331,	155.0819903	29823	12.92	5	8	9.04	other, protein binding	protein folding	nucleus	
125	(O43516) Wiskott-Aldrich syndrome protein-interact	WASIP_HUMAN	O43516	WIPF1,WASPIP, WIP,	175.29	51243	12.04	5	8	11.47	other, protein binding	cell structure	cytoplasm	nucleus
126	(P04075) Fructose-bisphosphate aldolase A (EC 4.1	ALDOA_HUMAN	P04075	ALDOA,ALDA,	172.0669089	39264	12.64	6	8	8.39	other, transporter	cell metabolism	cytoplasm	plasma membrane
127	(Q9UKM9) RNA-binding protein Raly (hnRNP associ	RALY_HUMAN	Q9UKM9	RALY,HNRPCL2, P542,	157.9869089	32444	15.65	6	8	9.2	RNA binding	RNA processing	nucleus	
128	(Q9BQE6) MGC2477 protein	Q9BQE6_HUMAN	Q9BQE6	C11orf48	454.27	18384	38.92	8	8	4.52	unknown	unknown	unknown	
129	(Q14103) Heterogeneous nuclear ribonucleoprotein	HNRPD_HUMAN	Q14103	HNRPD,AUF1,	181.0938177	38410	9.74	3	7	7.61	DNA/RNA binding	transcription, RNA splicing	nucleus	cytoplasm
130	(Q9Y3U8) 60S ribosomal protein L36	RL36_HUMAN	Q9Y3U8	RPL36	110.23	12115	18.18	3	7	11.59	stuctural component of ribosome	protein biosynthesis	cytoplasm	nucleus
131	(P45973) Chromobox protein homolog 5 (Heterochr	CBX5_HUMAN	P45973	CBX5,HP1A,	140.6669089	22211	9.95	4	7	5.71	other, protein binding	chromatin organization ; transcription (-)	nucleus	cytoplasm
132	(Q9Y3B9) CGI-115 protein	Q9Y3B9_HUMAN	Q9Y3B9	CGI-115, KIAA0507,	229.8009952	28955	18.25	4	7	5.39	other, protein binding	translation	nucleus	
133	(P62888) 60S ribosomal protein L30	RL30_HUMAN	P62888	RPL30	151.4369089	12645	32.46	4	7	9.65	stuctural component of ribosome	protein biosynthesis	cytoplasm	nucleus
134	(P46013) Antigen KI-67	KI67_HUMAN	P46013	MKI67	128.2458081	358526	1.10	5	7	9.49	other, protein binding	cell cycle (+)	nucleus	
135	(P31948) Stress-induced-phosphoprotein 1 (STI1) (	STIP1_HUMAN	P31948	STIP1	173.6838177	62599	8.08	5	7	6.4	other, protein binding	protein folding	cytoplasm	nucleus
136	(P84090) Enhancer of rudimentary homolog	ERH_HUMAN	P84090	ERH	101.9719903	12251	14.41	2	6	5.62	unknown	unknown	unknown	
137	(Q12905) Interleukin enhancer-binding factor 2 (Nuc	ILF2_HUMAN	Q12905	NF45,PRO3063,	161.03	43035	7.93	3	6	5.19	DNA/RNA binding	transcription	nucleus	
138	(P42766) 60S ribosomal protein L35	RL35_HUMAN	P42766	RPL35	93.46	14412	14.50	3	6	11.04	stuctural component of ribosome	protein biosynthesis	cytoplasm	nucleus
139	(Q9H7B2) Brix domain-containing protein 1	BXDC1_HUMAN	Q9H7B2	BXDC1	90.88	35560	7.43	4	6	10	RNA binding	ribosome biogenesis	nucleus	
140	(P11021) 78 kDa glucose-regulated protein precurs	GRP78_HUMAN	P11021	HSPA5,GRP78,	179.67	72288	6.09	4	6	5.01	other, protein binding	protein folding ; apoptosis	endoplasmic reticulum	nucleus
141	(P14618) Pyruvate kinase isozymes M1/M2 (EC 2.7	KPYM_HUMAN	P14618	PKM2,PK2, PK3, PKM,	86.7	57769	4.95	4	6	7.95	other, metabolic enzyme, deshydrogenase	cell metabolism	cytoplasm	
142	(P40926) Malate dehydrogenase, mitochondrial pre	MDHM_HUMAN	P40926	MDH2	135.92	35509	8.95	4	6	8.54	other, metabolic enzyme, deshydrogenase	cell metabolism	cytoplasm	mitochondria
143	(Q9Y2X3) Nucleolar protein NOP5 (Nucleolar protei	NOP5_HUMAN	Q9Y2X3	NOP5,HSPC120,	129.95	59541	5.55	4	6	9.03	RNA binding	ribosome biogenesis	nucleus	
144	(Q96CT7) Hypothetical protein LOC115098	Q96CT7_HUMAN	Q96CT7	CCDC124	160.7269089	25820	13.68	4	6	9.54	unknown	unknown	unknown	
145	(Q01130) Splicing factor, arginine/serine-rich 2 (Spl	SFRS2_HUMAN	Q01130	SFRS2	190.8909952	25330	17.83	4	6	11.86	RNA binding	RNA processing, transcription (-)	nucleus	
146	(Q15365) Poly(rC)-binding protein 1 (Alpha-CP1) (hr	PCBP1_HUMAN	Q15365	PCBP1	215.72	37474	11.76	5	6	6.66	RNA binding	RNA processing	nucleus	
147	(P09651) Heterogeneous nuclear ribonucleoprotein	ROA1_HUMAN	P09651	HNRNPA1,HNRPA1,	178.77	38691	11.65	5	6	9.26	RNA binding	RNA processing	nucleus	cytoplasm
148	(P62277) 40S ribosomal protein S13	RS13_HUMAN	P62277	RPS13	190.1609952	17081	28.39	5	6	10.53	stuctural component of ribosome	protein biosynthesis	cytoplasm	nucleus
149	(P68104) Elongation factor 1-alpha 1 (EF-1-alpha-1	EF1A1_HUMAN	P68104	EEF1A1,EEF1A, EF1A, LENG7,	99.46	50109	4.40	2	5	9.1	other, protein binding	translation	cytoplasm	nucleus
150	(P62318) Small nuclear ribonucleoprotein Sm D3 (sı	SMD3_HUMAN	P62318	SNRPD3	110.7	13907	15.08	2	5	10.33	RNA binding	RNA processing	nucleus	
151	(P17026) Zinc finger protein 22 (Zinc finger protein	ZNF22_HUMAN	P17026	ZNF22,KOX15, KROX26,	74.44	25899	7.23	2	5	10.06	DNA binding	transcription	nucleus	
152	(Q8NEJ9) Protein C14orf120	CN120_HUMAN	Q8NEJ9	NGDN,C14orf120,	123.05	35872	9.82	3	5	9.57	other, protein binding	translation	nucleus	cytoplasm
153	(P20700) Lamin-B1	LMNB1_HUMAN	P20700	LMNB1,LMN2, LMNB,	111.3669089	66237	5.15	3	5	5.11	other, structural protein	cell structure	nucleus	
154	(Q53F64) Heterogeneous nuclear ribonucleoprotein	Q53F64_HUMAN	Q53F64		250.9407266	35973	13.46	3	5	7.28	RNA binding	RNA processing	nucleus	cytoplam
155	(P42696) RNA-binding protein 34 (RNA-binding moti	RBM34_HUMAN	P42696	RBM34,KIAA0117,	137.8869089	48535	7.03	3	5	10.11	RNA binding	unknown	nucleus	
156	(P63220) 40S ribosomal protein S21	RS21_HUMAN	P63220	RPS21	171.32	9106	25.30	3	5	8.68	stuctural component of ribosome	protein biosynthesis	cytoplasm	nucleus
157	(Q8WXX5) DnaJ homolog subfamily C member 9 (D	DNJC9_HUMAN	Q8WXX5	DNAJC9	115.6884727	29891	11.81	4	5	5.58	other, protein binding	protein folding	unknown	
158	(Q96BK5) Pin2-interacting protein X1 (TRF1-interac	PINX1_HUMAN	Q96BK5	PINX1,LPTL, LPTS,	157.3707266	37012	12.20	4	5	9.61	DNA/RNA/protein binding	cell proliferation, cell cycle (+)	nucleus	
159	(Q9NPA8) E(Y)2 homolog (DC6 protein) (Enhancer	Q9NPA8_HUMAN	Q9NPA8	ENY2,DC6,	100.4669089	11521	25.00	4	5	9.39	DNA/protein binding	transcription	unknown	
160	(P62913) 60S ribosomal protein L11 (CLL-associat	RL11_HUMAN	P62913	RPL11	168.4394679	20109	20.88	4	5	9.64	stuctural component of ribosome	protein biosynthesis	cytoplasm	nucleus
161	(Q15428) Splicing factor 3A subunit 2 (Spliceosome	SF3A2_HUMAN	Q15428	SF3A2,SAP62,	149.1309952	49224	8.50	4	5	9.65	RNA binding	RNA processing	nucleus	
162	(P37108) Signal recognition particle 14 kDa protein	SRP14_HUMAN	P37108	SRP14	195.5709952	14535	17.42	4	5	10.05	RNA binding	translation (-)	cytoplasm	nucleus
163	(Q9Y2W1) Thyroid hormone receptor-associated pr	TR150_HUMAN	Q9Y2W1	THRAP3,TRAP150,	80.07690887	108601	2.23	4	5	10.16	DNA binding	transcription (+)	nucleus	
164	(Q9H2V5) BR22 TTF-1 associated protein (Hypothe	Q9H2V5_HUMAN	Q9P031	BR22, TAP26,HSPC128,	245.2369089	28652	20.77	5	5	9.89	unknown	unknown	unknown	
165	(P62910) 60S ribosomal protein L32	RL32_HUMAN	P62910	RPL32,PP9932,	189.17	15719	30.99	5	5	11.32	stuctural component of ribosome	protein biosynthesis	cytoplasm	nucleus
166	(P56537) Eukaryotic translation initiation factor 6 (e	IF6_HUMAN	P56537	EIF3A, ITGB4BP,OK/SW-cl.27,	128.77	26582	9.96	2	4	4.56	other, protein binding	translation	cytoplasm	nucleus
167	(Q9UNZ5) Leydig cell tumor 10 kDa protein homoloc	L10K_HUMAN	Q9UNZ5	C19orf53,HSPC023,	53.62690887	10570	8.33	2	4	11.55	unknown	unknown	unknown	
168	(P62906) 60S ribosomal protein L10a (CSA-19)	RL10A_HUMAN	P62906	RPL10A,NEDD6,	67.2	24684	7.14	2	4	9.94	stuctural component of ribosome	protein biosynthesis	cytoplasm	nucleus
169	(P40429) 60S ribosomal protein L13a (23 kDa highl	RL13A_HUMAN	P40429	RPL13A	92.57690887	23431	10.33	2	4	10.94	stuctural component of ribosome	protein biosynthesis	cytoplasm	nucleus
170	(P32969) 60S ribosomal protein L9	RL9_HUMAN	P32969	RPL9,OK/SW-cl.103,RPL9P7, F	93.26690887	21850	10.61	2	4	9.96	stuctural component of ribosome	protein biosynthesis	cytoplasm	nucleus
171	(P39019) 40S ribosomal protein S19	RS19_HUMAN	P39019	RPS19	66.18690887	15919	13.19	2	4	10.31	stuctural component of ribosome	protein biosynthesis	cytoplasm	nucleus
172	(Q8TDN6) Brix domain-containing protein 2 (Riboso	BXDC2_HUMAN	Q8TDN6	BXDC2, BRIX	145.5169089	41375	7.18	3	4	9.92	RNA binding	ribosome biogenesis	nucleus	
173	(Q92522) Histone H1x	H1X_HUMAN	Q92522	H1FX	102.4384727	22474	13.24	3	4	10.76	DNA binding	chromatin organization	nucleus	
174	(Q9UKD2) mRNA turnover protein 4 homolog	MRT4_HUMAN	Q9UKD2	MRTO4, C1orf33, MRT4	71.26099517	27543	9.20	3	4	8.34	RNA binding	ribosome biogenesis	nucleus	cytoplam
175	(Q9NPE3) H/ACA ribonucleoprotein complex subunit	NOLA3_HUMAN	Q9NPE3	NOLA3,NOP10	345.2338177	7701	54.29	3	4	10.01	RNA binding	ribosome biogenesis and telomere maintenance	nucleus	
176	(P09874) Poly [ADP-ribose] polymerase 1 (EC 2.4.2	PARP1_HUMAN	P09874	PARP1,ADPRT, PPOL,	116.4269089	112881	3.70	3	4	8.99	DNA binding ; transferase	transcription ; DNA repair	nucleus	
177	(Q9NWB6) CDNA FLJ10154 fis, clone HEMBA1003	Q9NWB6_HUMAN	Q9NWB6	ARGLU1	64.44690887	33197	4.98	3	4	10.35	unknown	unknown	unknown	unknown
178	(Q9UI29) Adrenal gland protein AD-002 (HSPC148 p	Q9UI29_HUMAN	Q9P013	C11orf5,AD-002, HSPC148,	55.99790405	26608	9.96	3	4	5.55	unknown	unknown	nucleus	
179	(P84101) Small EDRK-rich factor 2 (4F5rel) (h4F5re	SERF2_HUMAN	P84101	SERF2,FAM2C,	66.34	6896	30.65	3	4	10.44	unknown	unknown	unknown	
180	(P84103) Splicing factor, arginine/serine-rich 3 (Pre	SFRS3_HUMAN	P84103	SFRS3,SRP20,	86.29	19318	11.43	3	4	11.64	RNA binding	RNA processing ; cell cycle	nucleus	

181	(P23284) Peptidyl-prolyl cis-trans isomerase B prec	PPIB_HUMAN	P23284	PPIB,CYPB,	180.37	22728	17.48	4	4	9.25	other, metabolic enzyme, deshydrogenase	protein folding	ER, cytoplasm	nucleus
182	(Q9NW13) RNA-binding protein 28 (RNA-binding mo	RBM28_HUMAN	Q9NW13	RBM28	75.29690887	85554	2.83	4	4	9.27	RNA binding	ribosome biogenesis	nucleus	
183	(P83916) Chromobox protein homolog 1 (Heterochr	CBX1_HUMAN	P83916	CBX1,CBX,	124.0769089	21405	8.25	1	3	4.85	other, protein binding	chromatin organization ; transcription (-)	nucleus	cytoplasm
184	(Q6DKI1) Ribosomal protein L7-like 1	RL7L_HUMAN	Q6DKI1	RPL7L1	61.96099517	28643	3.85	1	3	10.51	unknown	unknown	cytoplasm	nucleus
185	(P48047) ATP synthase O subunit, mitochondrial pr	ATPO_HUMAN	P48047	ATP5O,ATPO,	62.47	23263	9.48	2	3	9.81	other, transporter	cell metabolism	mitochondria	cytoplasm
186	(Q9NSI2) Protein C21orf70	CU070_HUMAN	Q9NSI2	C21orf70,PRED56,	103.27	25441	10.39	2	3	11.07	unknown	unknown	unknown	
187	(P16989) DNA-binding protein A (Cold shock domair	DBPA_HUMAN	P16989	CSDA,DBPA,	115.19	40066	10.22	2	3	9.77	DNA binding	transcription (-)	nucleus	cytoplasm
188	(Q9BYG3) MKI67 FHA domain-interacting nucleolar	MK67I_HUMAN	Q9BYG3	MKI67IP, NIFK, NOPP34	124.6409952	34201	7.74	2	3	9.88	RNA binding	ribosome biogenesis	nucleus	
189	(P55769) NHP2-like protein 1 (High mobility group-li	NHPX_HUMAN	P55769	NHP2L1	110.1	14034	18.11	2	3	8.72	RNA binding	ribosome biogenesis ; RNA splicing	nucleus	
190	(Q9NY12) H/ACA ribonucleoprotein complex subunit	NOLA1_HUMAN	Q9NY12	NOLA1,GAR1	105.05	22334	13.30	2	3	10.91	RNA binding	ribosome biogenesis ; telomere maintenance	nucleus	
191	(O43328) Testis-specific gene 118 protein	O43328_HUMAN	Q1ED39	TSG118	94.24381774	21529	11.79	2	3	9.58	unknown	unknown	nucleus	
192	(O00233) 26S proteasome non-ATPase regulatory s	PSD9_HUMAN	O00233	PSMD9	125.44	24638	9.87	2	3	6.46	other, protein binding	protein targeting and degradation	cytoplasm	nucleus
193	(P62826) GTP-binding nuclear protein Ran (GTPase	RAN_HUMAN	P62826	ARA24,OK/SW-cl.81,	68.07099517	24277	10.00	2	3	7.2	DNA/RNA/protein binding	chromatin organization	nucleus	cytoplam
194	(P60866) 40S ribosomal protein S20	RS20_HUMAN	P60866	RPS20	76.73	13364	19.01	2	3	9.95	stuctural component of ribosome	protein biosynthesis	cytoplasm	nucleus
195	(Q01105) Protein SET (Phosphatase 2A inhibitor I2	SET_HUMAN	Q01105	SET	117.2	33469	7.57	2	3	4.23	other, protein binding	chromatin organization	nucleus	, cytoplasm, ER
196	(Q07955) Splicing factor, arginine/serine-rich 1 (pre	SFRS1_HUMAN	Q07955	ASF, SF2, SF2P33,OK/SW-cl.3,	69.49690887	27597	7.60	2	3	10.37	RNA binding	RNA processing	nucleus	cytoplasm
197	(P40227) T-complex protein 1 subunit zeta (TCP-1-z	TCPZ_HUMAN	P40227	CCT6A,CCT6, CCTZ,	68.76690887	57857	3.62	2	3	6.25	other, protein binding	protein folding	cytoplasm	nucleus
198	(Q9NVP1) ATP-dependent RNA helicase DDX18 (E	DDX18_HUMAN	Q9NVP1	DDX18	83.29099517	75359	4.82	3	3	9.52	RNA binding ; helicase	translation	nucleus	
199	(Q92945) Far upstream element-binding protein 2 (F	FUBP2_HUMAN	Q92945	KHSRP,FUBP2,	133.14	72664	5.00	3	3	8.02	DNA/RNA binding	transcription ; RNA processing	nucleus	cyoplasm
200	(Q13601) HIV-1 Rev-binding protein 2 (Rev-interact	HRB2_HUMAN	Q13601	KRR1,HRB2,	108.9458081	43507	5.82	3	3	9.78	RNA binding	RNA processing	nucleus	
201	(P12004) Proliferating cell nuclear antigen (PCNA) (	PCNA_HUMAN	P12004	PCNA	218.18	28750	13.03	3	3	4.57	DNA binding	cell proliferation, cell cycle, DNA repair, replication	nucleus	
202	(P46783) 40S ribosomal protein S10	RS10_HUMAN	P46783	RPS10	142.4769089	18886	19.30	3	3	10.15	stuctural component of ribosome	protein biosynthesis	cytoplasm	nucleus
203	(P07108) Acyl-CoA-binding protein (ACBP) (Diazepa	ACBP_HUMAN	P07108	DBI	92.94099517	9907	14.44	1	2	6.11	other, metabolic enzyme, deshydrogenase	cell metabolism	cytoplasm	nucleus, mitochondria
204	(Q8NDC0) Protein C14orf32	CN032_HUMAN	Q8NDC0	C14orf32	75.13099517	24122	7.76	1	2	5.3	unknown	unknown	nucleus	
205	(O75607) Nucleoplasmin-3	NPM3_HUMAN	O75607	NPM3	88.73690887	19200	9.20	1	2	4.55	other, protein binding	chromatin organization	nucleus	
206	(Q86U42) Polyadenylate-binding protein 2 (Poly(A)-l	PABP2_HUMAN	Q86U42	PABPN1,PAB2, PABP2,	78.35690887	32598	5.41	1	2	5.04	RNA binding	RNA processing	nucleus	cytoplasm
207	(Q6NX36) Hypothetical protein C1orf174	Q6NX36_HUMAN	Q8IYL3	C1orf174	96.96	26016	5.93	1	2	6.9	unknown	unknown	unknown	
208	(P46776) 60S ribosomal protein L27a	RL27A_HUMAN	P46776	RPL27A	119.94	16420	8.72	1	2	11	stuctural component of ribosome	protein biosynthesis	cytoplasm	nucleus
209	(P62306) Small nuclear ribonucleoprotein F (snRNP	RUXF_HUMAN	P62306	SNRPF,PBSCF,	92.91	9719	14.77	1	2	4.7	RNA binding	RNA processing	nucleus	
210	(P62316) Small nuclear ribonucleoprotein Sm D2 (sı	SMD2_HUMAN	P62316	SNRPD2,SNRPD1,	74.56099517	13518	13.11	1	2	9.92	RNA binding	RNA processing	nucleus	
211	(P61981) 14-3-3 protein gamma (Protein kinase C i	1433G_HUMAN	P61981	YWHAG	65.57099517	28154	6.67	2	2	4.8	other, protein binding	signal transduction ; apoptosis (-), cell cycle	cytoplasm	nucleus
212	(P13639) Elongation factor 2 (EF-2)	EF2_HUMAN	P13639	EEF2,EF2,	52.08	95146	2.31	2	2	6.42	other, protein binding	translation	cytoplasm	
213	(P38646) Stress-70 protein, mitochondrial precurso	GRP75_HUMAN	P38646	HSPA9,GRP75, HSPA9B,	69.4	73635	1.79	2	2	5.44	other, protein binding	protein folding ; apoptosis (-)	cytoplasm, mitochondria	nucleus
214	(O15525) Transcription factor MafG (V-maf musculo	MAFG_HUMAN	O15525	MAFG	121.84	17839	13.58	2	2	10.04	DNA/protein binding	transcription; cell cycle	nucleus	
215	(O60675) Transcription factor MafK (Erythroid trans	MAFK_HUMAN	O60675	MAFK	79.07	17512	12.58	2	2	10.1	DNA/protein binding	transcription	nucleus	
216	(P26599) Polypyrimidine tract-binding protein 1 (PTI	PTBP1_HUMAN	P26599	PTBP1,PTB,	67.78099517	57186	4.24	2	2	9.22	RNA binding	RNA processing	nucleus	
217	(P46782) 40S ribosomal protein S5	RS5_HUMAN	P46782	RPS5	63.41	22731	8.25	2	2	9.73	stuctural component of ribosome	protein biosynthesis	cytoplasm	nucleus
218	(Q16629) Splicing factor, arginine/serine-rich 7 (Spl	SFRS7_HUMAN	Q16629	SFRS7	83.51	27350	8.06	2	2	11.83	RNA binding	RNA processing	nucleus	
219	(Q93045) Stathmin-2 (SCG10 protein) (Superior cer	STMN2_HUMAN	Q93045	STMN2,SCG10, SCGN10,	109.3709952	20815	10.58	1	1	8.4	other, protein binding	cell structure	cytoplasm	
220	(Q96FF9) Sororin (Cell division cycle-associated pr	CDCA5_HUMAN	Q96FF9	CDCA5	66.99	27583	5.60	1	1	9.65	other, protein binding	chromatin organization ; cell cyle	nucleus	cytoplam (mitosis)
221	(P23528) Cofilin-1 (Cofilin, non-muscle isoform) (18	COF1_HUMAN	P23528	CFL1,CFL,	75.86099517	18360	7.23	1	1	8.26	other, protein binding	cell structure	cytoplasm	nucleus
222	(P14854) Cytochrome c oxidase subunit VIb isofor	CX6B1_HUMAN	P14854	COX6B1,COX6B,	175.3609952	10055	26.37	1	1	6.79	other, transporter	cell metabolism	mitochondria	nucleus, cytoplam
223	(P38919) Probable ATP-dependent RNA helicase D	DDX48_HUMAN	P38919	EIF4A3,DDX48, KIAA0111,	95.78481292	46710	5.90	1	1	6.33	RNA binding ; helicase	translation	nucleus	
224	(Q7Z6I8) Hypothetical protein C5orf24	Q7Z6I8_HUMAN	Q7Z6I8	C5orf24	80.91	20119	7.69	1	1	9.87	unknown	unknown	unknown	
225	(Q92646) H2A.k	Q92646_HUMAN	Q92646	HIST1H2APS4,H2A/k,	74.13	12646	8.77	1	1	10.9	unknown	unknown	unknown	
226	(Q96C57) Hypothetical protein C12orf43	Q96C57_HUMAN	Q96C57	C12orf43	71.84	28240	6.25	1	1	9.44	unknown	unknown	unknown	
227	(Q9BRT6) Hypothetical protein C12orf31 (CDNA FL	Q9BRT6_HUMAN	Q9BRT6	C12orf31	128.33	15215	15.94	1	1	10.38	unknown	unknown	unknown	
228	(P05387) 60S acidic ribosomal protein P2 (NY-REN	RLA2_HUMAN	P05387	RPLP2,D11S2243E, RPP2,	74.07	11658	11.43	1	1	4.42	stuctural component of ribosome	protein biosynthesis	cytoplasm	nucleus
229	(Q9Y2S0) DNA-directed RNA polymerase I subunit I	RPO1D_HUMAN	Q9Y2S0	POLR1D	65.38099517	15227	7.25	1	1	5.55	DNA binding	transcription	nucleus	
230	(P62847) 40S ribosomal protein S24	RS24_HUMAN	P62847	RPS24	60.78690887	15413	5.71	1	1	10.79	stuctural component of ribosome	protein biosynthesis	cytoplasm	nucleus
231	(P49458) Signal recognition particle 9 kDa protein (:	SRP09_HUMAN	P49458	SRP9	100.37	9974	17.78	1	1	8.28	RNA binding	translation (-)	cytoplasm	nucleus
232	(P16949) Stathmin (Phosphoprotein p19) (pp19) (O	STMN1_HUMAN	P16949	STMN1,LAP18, OP18,	108.3238177	17161	12.82	1	1	5.77	other, protein binding	cell structure	cytoplasm	
233	(O75683) Surfeit locus protein 6	SURF6_HUMAN	O75683	SURF6,SURF-6	132.88	41426	8.24	1	1	10.65	RNA binding	ribosome biogenesis	nucleus	
234	(Q12931) Heat shock protein 75 kDa, mitochondrial	TRAP1_HUMAN	Q12931	TRAP1,HSP75,	84.27	80060	1.93	1	1	8.28	other, protein binding	protein folding	mitochondria	cytoplasm
235	(P47985) Ubiquinol-cytochrome c reductase iron-su	UCRI_HUMAN	P47985	UQCRFS1	135.5469089	29633	10.78	1	1	6.3	other, metabolic enzyme, deshydrogenase	cell metabolism	mitochondria	
236	(P67809) Nuclease sensitive element-binding protei	YBOX1_HUMAN	P67809	YBX1,NSEP1, YB1,	105.2	35772	5.85	1	1	9.87	DNA/RNA binding	transcription, RNA processing, cell cycle	nucleus	cytoplasm

[1]Retrieved from Uniprot

Variable modifications allowed for Mascot searches :
acetyl-lysine
dimethyl-lysine
methyl-lysine
serine and threonine phosphorylation
protein N-acetylation
peptide N-terminal acetylation
methionine oxidation
methionine sulphone
cysteic acid

Table S2: Genes significantly modulated between Raji shCtrl and Raji shCYCLON cell lines

Notes : Created from Advanced Analysis operation: significance Analysis.
#Entitylist : 3465r_ShcyclonvsShCtrl_FC>= 1.2
#Interpretation : Group_ShcyclonvsShCtrl
#corrected p-value cut-off:0.05
#Selected Test : T Test unpaired
#p-value computation: Asymptotic
#Multiple Testing Correction: Benjamini-Hochberg
Technology : Affymetrix.GeneChip.HG-U133_Plus_2

Probe Set ID	Regulation	Gene Symbol	FC
1554747_a_at	up	SEPT2	1.3452525
210348_at	up	SEPT4	1.2318484
201308_s_at	up	SEPT11	1.5969949
202852_s_at	up	AAGAB	1.3515162
202170_s_at	up	AASDHPPT	1.2595851
1555039_a_at	up	ABCC4	1.5179213
213805_at	up	ABHD5	1.2745662
221679_s_at	up	ABHD6	1.2033099
210461_s_at	up	ABLIM1	1.3266736
242081_at	down	ACAP1	-1.2228346
234312_s_at	up	ACSS2	1.210379
200727_s_at	up	ACTR2	1.6853753
208223_s_at	up	ACVR1B	1.2258034
207422_at	down	ADAM20	-1.2314298
244463_at	down	ADAM23	-1.2529302
241446_at	down	ADAM28	-1.2574718
1555326_a_at	up	ADAM9	1.2373784
229004_at	down	ADAMTS15	-1.215619
228911_at	down	ADAMTS7	-1.2000039
222117_s_at	up	ADCK2	1.2364966
239530_at	down	ADD2	-1.2626154
211491_at	up	ADRA1A	1.2238052
210957_s_at	up	AFF2	1.2449454
205734_s_at	up	AFF3	1.2646971
1565034_s_at	up	AFF3 /// MLL	1.2641628
213926_s_at	up	AGFG1	1.8158666
207293_s_at	up	AGTR2	1.2052976
243793_at	down	AHDC1	-1.2500672
208325_s_at	up	AKAP13	1.2306771
241978_at	up	AKR1A1	1.347034
203809_s_at	down	AKT2	-1.209167
203609_s_at	up	ALDH5A1	1.3379878
204290_s_at	up	ALDH6A1	1.2529459
211618_s_at	down	ALPI	-1.227935
1553219_a_at	up	AMMECR1	1.5873324
206121_at	down	AMPD1	-1.2286934
239651_at	up	ANAPC5	1.4114015
205389_s_at	down	ANK1	-1.2072151
207950_s_at	up	ANK3	1.2679877
219868_s_at	up	ANKFY1	1.2791767
233292_s_at	up	ANKHD1 /// ANKHD1-EIF4EBP3	1.4842196
226663_at	up	ANKRD10	1.4403979
216563_at	down	ANKRD12	-1.2450268
231423_s_at	up	ANKRD16	1.2168254
236421_at	up	ANKRD45	1.2065878
219496_at	down	ANKRD57	-1.2382185
229128_s_at	down	ANP32E	-1.2317584
208816_x_at	up	ANXA2P2	1.2402707
204894_s_at	down	AOC3	-1.2256814
230413_s_at	up	AP1S2	1.4901165

1555733_s_at	up	AP1S3	1.4179875
223237_x_at	down	AP2A1	-1.2146109
219994_at	up	APBB1IP	1.2727188
202631_s_at	up	APPBP2	1.2402741
214483_s_at	up	ARFIP1	1.2043763
228477_at	up	ARGLU1	1.7832876
202547_s_at	up	ARHGEF7	1.2464342
210649_s_at	down	ARID1A	-1.2068951
225184_at	up	ARID1B	1.2046044
231016_s_at	up	ARNT	1.4634826
208736_at	up	ARPC3	1.6490312
207284_s_at	down	ASPH	-1.2005804
219918_s_at	down	ASPM	-1.2408969
218659_at	down	ASXL2	-1.230501
1558807_at	up	ATAD2B	1.2740762
208442_s_at	up	ATM	1.4249728
209186_at	down	ATP2A2	-1.2545114
207521_s_at	up	ATP2A3	1.2174581
217603_at	up	ATP6V0A2	1.250147
214149_s_at	up	ATP6V0E1	1.5006995
212384_at	up	ATP6V1G2 /// BAT1	1.3440068
1569773_at	up	ATP8A1	1.4654691
214594_x_at	down	ATP8B1	-1.257413
211022_s_at	up	ATRX	1.6874208
212113_at	up	ATXN7L3B	1.5715481
227905_s_at	down	AZI2	-1.2240304
219326_s_at	up	B3GNT2	1.3589712
210535_at	down	B9D1	-1.2588447
222780_s_at	down	BAALC	-1.2152404
210125_s_at	down	BANF1	-1.2180156
214201_x_at	down	BAT2	-1.2025415
230618_s_at	up	BAT2L2	1.5455139
227896_at	down	BCCIP	-1.2400014
1557258_a_at	up	BCL10	1.2613658
203684_s_at	up	BCL2	1.3407803
224227_s_at	up	BDP1	1.3714335
220451_s_at	down	BIRC7	-1.2500215
226853_at	down	BMP2K	-1.2142243
210523_at	down	BMPR1B	-1.3424524
210214_s_at	up	BMPR2	1.344528
204930_s_at	up	BNIP1	1.2533311
235009_at	up	BOD1L	1.7748537
232698_at	up	BPIL1	1.237938
41512_at	down	BRAP	-1.2698414
222737_s_at	down	BRD7	-1.2065537
206787_at	down	BRDT	-1.21183
218954_s_at	down	BRF2	-1.2064899
240362_at	up	BRP44L	1.3525517
1553252_a_at	up	BRWD3	1.3571057
201235_s_at	up	BTG2	1.3849775
215509_s_at	up	BUB1	1.3521194
209182_s_at	up	C10orf10	1.2326845
229399_at	up	C10orf118	1.6101971
227136_s_at	down	C10orf46	-1.2587973
239998_at	up	C10orf53	1.2944402
227099_s_at	down	C11orf96	-1.2407529
227152_at	up	C12orf35	1.4074843
229778_at	up	C12orf39	1.2305417
226349_at	down	C12orf45	-1.2465434
227928_at	down	C12orf48	-1.2019851
236853_at	down	C13orf16	-1.2512114

231859_at	up	C14orf132	1.2126943
237640_at	up	C14orf138	1.3045775
224798_s_at	up	C15orf17	1.2529098
221300_at	down	C15orf2	-1.2586917
231338_at	up	C15orf55	1.2323191
225088_at	up	C16orf63	1.4222207
213815_x_at	down	C19orf29	-1.221676
232730_at	down	C19orf44	-1.2715486
212574_x_at	up	C19orf6	1.6493751
219439_at	down	C1GALT1	-1.2167912
238365_s_at	up	C1orf228	1.2860056
223459_s_at	down	C1orf56	-1.2008423
222301_at	up	C1orf61	1.2541444
202953_at	up	C1QB	1.2004935
205575_at	up	C1QL1	1.2549008
223499_at	up	C1QTNF5 /// MFRP	1.2410158
232619_at	down	C20orf134	-1.2012147
234002_at	down	C20orf135	-1.2864715
231991_at	up	C20orf160	1.220118
1559252_a_at	down	C20orf29	-1.2932229
227867_at	up	C2orf89	1.2594761
225279_s_at	up	C3orf17	1.310732
224273_at	down	C3orf20	-1.2145138
1569374_at	up	C3orf62	1.4442676
1562832_at	down	C3orf65	-1.3066753
1554192_s_at	up	C4orf23	1.2053691
1552660_a_at	up	C5orf22	1.7116319
229443_at	up	C6orf125	1.2189027
213875_x_at	up	C6orf62	1.6402713
224988_at	up	C6orf89	1.2278509
1564658_at	down	C7orf52	-1.2618679
219455_at	up	C7orf63	1.2178006
241781_at	up	C9orf41	1.4025706
224311_s_at	up	CAB39	1.6028811
210691_s_at	down	CACYBP	-1.2209071
215259_s_at	up	CADM4	1.2313446
243084_at	down	CALD1	-1.220924
214845_s_at	up	CALU	1.3490969
1552768_at	up	CAMKK1	1.2035276
208853_s_at	up	CANX	1.2782067
202966_at	down	CAPN6	-1.228815
200722_s_at	up	CAPRIN1	1.261952
212512_s_at	down	CARM1	-1.2410667
240983_s_at	up	CARS	1.2952435
207620_s_at	up	CASK	1.2381201
211140_s_at	up	CASP2	1.2327163
207686_s_at	up	CASP8	1.274467
211922_s_at	up	CAT	1.2101026
226085_at	down	CBX5	-1.3132952
226539_s_at	up	CCDC42B	1.513871
223815_at	up	CCDC45	1.2056692
231657_s_at	up	CCDC74B	1.2446398
203119_at	down	CCDC86	-1.5212156
219387_at	up	CCDC88A	1.4102992
205392_s_at	up	CCL14 /// CCL14-CCL15	1.2726742
213226_at	down	CCNA2	-1.2112991
214710_s_at	down	CCNB1	-1.2857515
211814_s_at	up	CCNE2	1.4793905
206337_at	up	CCR7	1.2161129
220351_at	down	CCRL1	-1.3955326
220671_at	up	CCRN4L	1.2673248

210436_at	up	CCT8	1.3461922
208653_s_at	up	CD164	1.274331
209582_s_at	up	CD200	1.3509473
205264_at	down	CD3EAP	-1.2416427
205153_s_at	up	CD40	1.2441132
207549_x_at	up	CD46	1.5851563
1555746_at	down	CD79B	-1.3073603
204440_at	up	CD83	1.2129368
211192_s_at	up	CD84	1.6923444
202870_s_at	down	CDC20	-1.2372586
205167_s_at	down	CDC25C	-1.2311934
235787_at	down	CDC37L1	-1.235508
203377_s_at	up	CDC40	1.2268724
209287_s_at	up	CDC42EP3	1.2792972
218063_s_at	up	CDC42EP4	1.2054125
223674_s_at	up	CDC42SE1	1.4024023
209055_s_at	up	CDC5L	1.2400482
218399_s_at	down	CDCA4	-1.2088729
201130_s_at	up	CDH1	1.2587872
207172_s_at	down	CDH11	-1.204747
231534_at	up	CDK1	1.3227781
207319_s_at	up	CDK13	1.516393
206474_at	up	CDK17	1.2785498
211804_s_at	up	CDK2	1.3398486
207188_at	up	CDK3	1.2244807
207143_at	up	CDK6	1.2442181
1553112_s_at	up	CDK8	1.2156193
236023_at	up	CDK9	1.2284026
209714_s_at	down	CDKN3	-1.2462797
213548_s_at	up	CDV3	1.6756972
204113_at	up	CELF1	1.3241057
210821_x_at	down	CENPA	-1.2390207
205046_at	down	CENPE	-1.2900797
220885_s_at	up	CENPJ	1.3188705
228559_at	down	CENPN	-1.2548182
1554606_at	up	CEP120	1.3448977
221683_s_at	up	CEP290	1.2106994
213957_s_at	up	CEP350	1.2438337
238154_at	down	CEP70	-1.2000693
207645_s_at	up	CHD1L	1.3562883
1555463_a_at	up	CHD6	1.2927212
220630_s_at	up	CHIA	1.2619979
211358_s_at	down	CIZ1	-1.2203966
229610_at	up	CKAP2L	1.2619487
206164_at	down	CLCA2	-1.3078173
205149_s_at	up	CLCN4	1.3501853
232128_s_at	down	CLCN5	-1.2090104
221328_at	up	CLDN17	1.3624657
201559_s_at	up	CLIC4	1.2409272
201768_s_at	up	CLINT1	1.2812784
1558924_s_at	up	CLIP1	1.6365954
211136_s_at	up	CLPTM1	1.2145925
229777_at	down	CLRN3	-1.2053759
1553120_at	up	CLSPN	1.2195165
205518_s_at	down	CMAH	-1.2105225
224991_at	down	CMIP	-1.2962687
224998_at	down	CMTM4	-1.2570964
203239_s_at	down	CNOT3	-1.2588922
223465_at	up	COL4A3BP	1.4276825
229600_s_at	down	CPD	-1.2248484
224828_at	up	CPEB4	1.6389138

241706_at	down	CPNE8	-1.2941418
206184_at	up	CRKL	1.2209756
1554464_a_at	up	CRTAP	1.2234758
207531_at	down	CRYGC	-1.27568
1568620_at	up	CSAD	1.2058828
1570169_at	up	CSMD2	1.2031901
240228_at	up	CSMD3	1.2381549
208867_s_at	up	CSNK1A1	1.2152762
222015_at	up	CSNK1E	1.2865859
212075_s_at	up	CSNK2A1	1.2449902
1555106_a_at	up	CTDSPL2	1.2179205
202213_s_at	up	CUL4B	1.2547195
220724_at	down	CWH43	-1.2131425
212977_at	up	CXCR7	1.3204342
235442_at	up	CXorf56	1.4556006
1431_at	up	CYP2E1	1.2867599
207786_at	up	CYP2R1	1.2100302
216607_s_at	up	CYP51A1	1.3477283
202879_s_at	up	CYTH1	1.2249681
1554335_at	up	CYTH4	1.2090207
219837_s_at	up	CYTL1	1.2000073
203890_s_at	up	DAPK3	1.7849464
236707_at	up	DAPP1	1.2138002
205369_x_at	up	DBT	1.2331996
216389_s_at	up	DCAF11	1.3267969
221851_at	down	DCAF15	-1.2154462
239187_at	up	DCAF16	1.2104834
231921_at	down	DCAF17	-1.2542593
1554558_at	down	DCAF5	-1.2519573
221224_s_at	up	DCAKD	1.2103885
235258_at	down	DCP2	-1.2283409
205338_s_at	down	DCT	-1.2673953
208675_s_at	up	DDOST	1.3880887
210749_x_at	up	DDR1	1.2399544
213998_s_at	up	DDX17	1.4270669
212514_x_at	up	DDX3X	1.4152759
1559954_s_at	up	DDX42	1.5512836
234347_s_at	up	DENR	1.260583
226980_at	down	DEPDC1B	-1.2741364
203695_s_at	up	DFNA5	1.239448
202099_s_at	up	DGCR2	1.2059903
1566038_at	up	DGCR7	1.2371081
234728_s_at	up	DHX35	1.4181397
216870_x_at	down	DLEU2	-1.2148956
215629_s_at	down	DLEU2 /// DLEU2L	-1.2359343
1561617_at	down	DNAH6	-1.2059814
203810_at	down	DNAJB4	-1.2782338
1555360_a_at	up	DNAJC11	1.2229487
1554451_s_at	up	DNAJC14	1.203102
223505_s_at	up	DNAJC27	1.2841597
232240_at	up	DNHD1	1.2583019
234942_s_at	down	DNTTIP1	-1.2394392
222004_s_at	up	DOCK6	1.2252464
239027_at	up	DOCK8	1.3660889
219452_at	up	DPEP2	1.2691046
226721_at	down	DPY19L4	-1.2199434
1554536_at	up	DPYD	1.3013089
205493_s_at	up	DPYSL4	1.2052408
203258_at	down	DRAP1	-1.2713146
204455_at	down	DST	-1.2563896
230402_at	down	DUSP15	-1.2101262

204794_at	up	DUSP2	1.2044349
226440_at	up	DUSP22	1.3387105
208892_s_at	down	DUSP6	-1.2646854
229115_at	up	DYNC1H1	1.308681
203692_s_at	up	E2F3	1.2034937
233850_s_at	down	EBF4	-1.2579534
219424_at	up	EBI3	1.2312487
230659_at	down	EDEM1	-1.2852004
219833_s_at	down	EFHC1	-1.226724
227283_at	down	EFR3B	-1.2550792
1565484_x_at	down	EGFR	-1.2807788
222314_x_at	down	EGOT	-1.2756013
205249_at	up	EGR2	1.2305626
209037_s_at	up	EHD1	1.2235167
222576_s_at	up	EIF2C1	1.6433426
205321_at	up	EIF2S3	1.2300643
1555996_s_at	up	EIF4A2	1.5830824
211937_at	up	EIF4B	1.254732
1554310_a_at	up	EIF4G3	1.2433524
220363_s_at	up	ELMO2	1.235109
204398_s_at	up	EML2	1.5175923
220386_s_at	up	EML4	1.2657421
237691_x_at	up	ENO3	1.2202686
204142_at	down	ENOSF1	-1.202513
205066_s_at	up	ENPP1	1.2224588
220153_at	up	ENTPD7	1.2953317
217254_s_at	down	EPO	-1.2142091
209962_at	down	EPOR	-1.3258492
200841_s_at	up	EPRS	1.5490665
210385_s_at	up	ERAP1	1.301499
217611_at	up	ERICH1	1.2212138
202444_s_at	up	ERLIN1	1.6385611
1555830_s_at	up	ESYT2	1.265215
226432_at	up	ETNK1	1.2364532
211825_s_at	up	EWSR1 /// FLI1	1.3454281
218748_s_at	up	EXOC5	1.5498394
1553947_at	up	EXOSC6	1.3065883
214734_at	down	EXPH5	-1.2002375
214608_s_at	up	EYA1	1.2200563
211310_at	up	EZH1	1.5040091
208621_s_at	up	EZR	1.7633995
207810_at	down	F13B	-1.2223849
222511_x_at	up	FAF1	1.8919816
212106_at	up	FAF2	1.2984308
1557128_at	up	FAM111B	1.2446389
1555947_at	down	FAM120A	-1.4169422
1552882_a_at	down	FAM123B	-1.2533257
230519_at	up	FAM124A	1.2670133
231874_at	up	FAM126B	1.3884314
227828_s_at	up	FAM176A	1.2805197
1554132_a_at	up	FAM190B	1.4590812
232217_at	down	FAM26E	-1.2108623
1554165_at	down	FAM53B	-1.2080717
235030_at	up	FAM55C	1.3563026
1559950_at	down	FAM66C /// FAM66D	-1.4033489
216897_s_at	up	FAM76A	1.3062598
1553749_at	down	FAM76B	-1.2745601
241481_at	down	FAM81A	-1.2045404
238741_at	down	FAM83A	-1.2098011
1557217_a_at	up	FANCB	1.2661111
1554277_s_at	up	FANCM	1.3058311

215068_s_at	down	FBXL18	-1.33131
1553500_at	down	FBXL21	-1.2053086
226970_at	down	FBXO33	-1.3342328
1559094_at	up	FBXO9	1.2685993
209456_s_at	up	FBXW11	1.4762064
218831_s_at	up	FCGRT	1.2031544
1553196_a_at	up	FCRL3	1.3161232
229989_at	up	FDXACB1	1.2486943
222245_s_at	up	FER1L4	1.2021043
210973_s_at	up	FGFR1	1.2479326
223263_s_at	up	FGFR1OP2	1.2579206
219718_at	up	FGGY	1.2104558
226769_at	up	FIBIN	1.2131882
76897_s_at	up	FKBP15	1.5776329
204560_at	up	FKBP5	1.4996216
224288_x_at	up	FKSG49	1.3765793
227302_s_at	up	FLII /// LLGL1	1.201744
205511_at	up	FLJ10038	1.4245838
224073_at	up	FLJ20464	1.2149084
1553145_at	up	FLJ39653	1.4630386
1557372_at	up	FLJ41757	1.2652222
211299_s_at	up	FLOT2	1.2812767
219316_s_at	up	FLVCR2	1.2433511
232249_at	up	FMNL3	1.3272188
235101_at	up	FNBP4	1.2754292
242029_at	up	FNDC3B	1.3083805
1555247_a_at	up	FNIP1 /// RAPGEF6	1.2547759
228463_at	down	FOXA3	-1.2123741
208006_at	up	FOXI1	1.208199
217310_s_at	up	FOXJ3	1.3377043
218031_s_at	up	FOXN3	1.2644747
210655_s_at	up	FOXO3 /// FOXO3B	1.3594829
1563687_a_at	up	FRYL	1.2577755
223985_at	up	FSD1L	1.2842823
223120_at	up	FUCA2	1.2420087
204452_s_at	up	FZD1	1.2272542
210220_at	up	FZD2	1.2654998
223254_s_at	up	G2E3	1.2814575
242422_at	up	G3BP1	1.6025373
214987_at	up	GAB1	1.2537209
203146_s_at	up	GABBR1	1.2307272
211679_x_at	up	GABBR2	1.2047298
232708_at	up	GALT	1.2353748
217445_s_at	up	GART	1.4258344
205848_at	up	GAS2	1.2561656
207704_s_at	up	GAS7	1.212802
214711_at	up	GATC	1.2177708
1555125_at	up	GCFC1	1.4320351
221279_at	up	GDAP1	1.450111
229868_s_at	down	GDF15	-1.287701
221576_at	up	GDF15	1.2748805
1555606_a_at	down	GDPD1	-1.200385
231918_s_at	up	GFM2	1.2053763
1554670_at	up	GGA1	1.3941542
214190_x_at	up	GGA2	1.3312801
236583_at	down	GIMAP1	-1.2174828
1553058_at	up	GIPC3	1.241221
207387_s_at	up	GK	1.2935873
215966_x_at	up	GK3P	1.2335669
219933_at	down	GLRX2	-1.2033429
223079_s_at	up	GLS	1.2266082

215794_x_at	up	GLUD2	1.309881
1554712_a_at	down	GLYATL2	-1.2565421
222115_x_at	up	GLYR1	1.2689847
200744_s_at	up	GNB1	1.2560008
46947_at	up	GNL3L	1.2525293
211522_s_at	down	GNRHR	-1.2125251
208797_s_at	up	GOLGA8A	1.4147563
204324_s_at	up	GOLIM4	1.3684632
239159_at	up	GOSR2	1.2846826
212487_at	up	GPATCH8	1.2186728
202756_s_at	up	GPC1	1.2246386
232195_at	down	GPR158	-1.2056496
244617_at	down	GPR26	-1.3236516
228313_at	down	GPRC5B	-1.2442926
206620_at	up	GRAP	1.2719648
233171_at	down	GRIN3A	-1.2056001
215766_at	up	GSTA1	1.3533078
238585_at	up	GTDC1	1.2703116
210620_s_at	up	GTF3C2	1.2934701
204318_s_at	down	GTSE1	-1.318172
1555568_at	up	GUSBL1	1.4084787
221892_at	up	H6PD	1.230185
209819_at	down	HABP4	-1.2562569
218602_s_at	down	HAUS6	-1.2056735
240602_at	up	HBS1L	1.2289792
1554478_a_at	up	HEATR3	1.204536
1557100_s_at	up	HECTD1	1.4062873
219319_at	down	HIF3A	-1.206478
226537_at	up	HINT3	1.2206274
205425_at	up	HIP1	1.3270811
217427_s_at	up	HIRA	1.4724717
207982_at	up	HIST1H1T	1.2245693
215071_s_at	up	HIST1H2AC	1.2059689
214516_at	down	HIST1H4B	-1.2549939
1560485_at	up	HIVEP1	1.2035419
221978_at	up	HLA-F	1.2324376
205822_s_at	up	HMGCS1	1.7307211
201055_s_at	up	HNRNPA0	1.2105442
213470_s_at	up	HNRNPH1	1.3795176
209675_s_at	down	HNRNPUL1	-1.2262769
219976_at	down	HOOK1	-1.2269777
213150_at	up	HOXA10	1.2043763
231121_at	down	HPS3	-1.239754
231705_at	up	HRSP12	1.3997586
211220_s_at	up	HSF2	1.2357876
1557910_at	up	HSP90AB1	1.560843
216450_x_at	up	HSP90B1	1.2380035
211016_x_at	up	HSPA4	1.5372337
215485_s_at	up	ICAM1	1.2222471
1552837_at	down	ICK	-1.2683991
209293_x_at	up	ID4	1.2500161
208965_s_at	up	IFI16	1.2847668
1555464_at	up	IFIH1	1.555285
216494_at	up	IGF2BP3	1.2441297
213674_x_at	up	IGHD	1.209107
216901_s_at	up	IKZF1	1.6100539
221092_at	up	IKZF3	1.9275341
207433_at	up	IL10	1.2236744
204773_at	up	IL11RA	1.2118208
228685_at	down	IL17RA	-1.2246972
224283_x_at	down	IL18BP	-1.2907579

208200_at	down	IL1A	-1.3095239
204863_s_at	up	IL6ST	1.2395563
208930_s_at	up	ILF3	1.3399829
219769_at	up	INCENP	1.479723
213544_at	down	ING2	-1.3011824
242293_at	up	ING3	1.2958503
243070_at	up	INO80D	1.3911563
208364_at	up	INPP4A	1.2509352
1554757_a_at	up	INPP5A	1.2216833
213643_s_at	up	INPP5B	1.2094048
201625_s_at	up	INSIG1	1.2569327
229633_at	down	INTS10	-1.2230217
228946_at	down	INTU	-1.2113076
215130_s_at	up	IQCK	1.2329689
238725_at	up	IRF1	1.2011575
224571_at	up	IRF2BP2	1.2547354
235182_at	down	ISM1	-1.2516865
209743_s_at	up	ITCH	1.2091734
1553530_a_at	up	ITGB1	1.2460012
1555349_a_at	up	ITGB2	1.3044101
204627_s_at	up	ITGB3	1.2276034
210888_s_at	up	ITIH1	1.2024969
213076_at	down	ITPKC	-1.2585015
206245_s_at	up	IVNS1ABP	1.2640836
224775_at	up	IWS1	1.2025434
215734_at	up	IZUMO4	1.6827525
1552610_a_at	up	JAK1	1.4068354
215138_s_at	up	KAZ	1.2018071
223765_s_at	up	KBTBD4	1.4288325
203204_s_at	up	KDM4A	1.2872592
235789_at	up	KDM4B	1.4039657
238220_at	up	KDM6A	1.2444586
220513_at	down	KHDC1L	-1.2078569
227555_s_at	up	KHSRP	1.3050393
201728_s_at	up	KIAA0100	1.4800811
210593_at	down	KIAA0913 /// SAT1	-1.3425449
228476_at	up	KIAA1407	1.2262238
223799_at	up	KIAA1826	1.5668621
232837_at	down	KIF13A	-1.2013
206364_at	down	KIF14	-1.3524646
218755_at	down	KIF20A	-1.2435501
201992_s_at	up	KIF5B	1.2963724
230701_x_at	down	KIF9	-1.2416157
211389_x_at	up	KIR3DS1	1.2067277
220825_s_at	down	KIRREL	-1.2244738
225596_at	up	KLC4	1.2337744
208960_s_at	up	KLF6	1.2428515
221986_s_at	up	KLHL24	1.6163739
1553027_a_at	up	KLHL4	1.2109339
1560397_s_at	up	KLHL6	1.5756903
216821_at	down	KRT8	-1.2562897
233681_at	down	KRTAP3-3	-1.2516819
224459_at	down	L2HGDH	-1.2553123
210644_s_at	up	LAIR1	1.4919143
219407_s_at	up	LAMC3	1.2146852
201551_s_at	up	LAMP1	1.416961
212193_s_at	down	LARP1	-1.2601091
233764_s_at	up	LARP1B	1.2555962
1555384_a_at	up	LARP4	1.2864332
1555643_s_at	up	LILRA5	1.2753243
201847_at	up	LIPA	1.2180349

203293_s_at	up	LMAN1	1.4436345
239057_at	down	LMOD2	-1.2352587
1560950_at	up	LOC100127950	1.2152765
224362_at	down	LOC100128922	-1.2007619
227318_at	up	LOC100131199	1.2275884
230799_at	up	LOC100134259	1.21235
231989_s_at	up	LOC100271836 /// LOC641298	1.4766943
215434_x_at	up	LOC100288142 /// NBPF1 /// NBPF10	1.4783069
226125_at	up	LOC100288152	1.2525893
223745_at	down	LOC100288525	-1.2110035
234344_at	down	LOC100288675	-1.366026
228207_at	down	LOC100499489	-1.3135792
217690_at	up	LOC100505523	1.2417207
231924_at	down	LOC100506305	-1.4148275
240917_at	up	LOC100506430	1.2669165
221109_at	up	LOC100506571	1.3075271
244600_at	up	LOC100506589	1.3543168
231559_at	down	LOC100506941	-1.2216151
243699_at	up	LOC100507006	1.279482
214316_x_at	down	LOC100507328 /// LOC100508591	-1.2219332
1555852_at	down	LOC100507463	-1.2310925
211973_at	up	LOC100507620	1.5277437
241521_at	up	LOC100507652	1.2427148
231501_at	down	LOC100508410	-1.2346087
213650_at	up	LOC100509749	1.3383579
1564287_at	down	LOC144776	-1.2229095
228779_at	down	LOC146880	-1.235323
1561059_a_at	down	LOC152024	-1.2213635
1564651_at	down	LOC221710	-1.2276598
216608_at	down	LOC26102	-1.2349784
230245_s_at	up	LOC283663	1.2801871
1556709_a_at	up	LOC283887	1.2242954
236854_at	up	LOC284749	1.2567853
1557267_s_at	up	LOC284952	1.411843
1562388_at	up	LOC285819	1.2171081
232663_s_at	up	LOC390595	1.260086
1569213_at	down	LOC400891	-1.2745079
227969_at	up	LOC400960	1.2160639
1569783_at	down	LOC401387	-1.2520658
240151_at	up	LOC404266	1.3112242
232001_at	down	LOC439949	-1.220347
215090_x_at	up	LOC440434	1.2332795
242222_at	up	LOC440894	1.2549862
1555860_x_at	up	LOC440944	1.3138562
235416_at	up	LOC643201	1.2415385
1558028_x_at	up	LOC647979	1.3630985
240436_at	down	LOC650794	-1.2401552
1570140_at	up	LOC653110	1.2342651
231987_at	up	LOC728264	1.2277015
1562257_x_at	down	LOC728392 /// NLRP1	-1.225269
229678_at	up	LOC728431	1.220079
228016_s_at	down	LOC729580	-1.2933202
1555058_a_at	up	LPGAT1	1.4418092
212272_at	up	LPIN1	1.2224839
1559580_at	down	LRRC39	-1.2333808
242112_at	up	LSM11	1.3246778
219653_at	up	LSM14B	1.7204089
209449_at	down	LSM2	-1.2162198
211019_s_at	up	LSS	1.4111693
1558173_a_at	up	LUZP1	1.2427858
210754_s_at	up	LYN	1.2250478

215222_x_at	up	MACF1	1.2028706
1552913_at	down	MAGEB18	-1.2360934
1555262_a_at	down	MAGI1	-1.2012762
224568_x_at	up	MALAT1	2.579687
208116_s_at	up	MAN1A1	1.4797127
215391_at	down	MAP1A	-1.2572525
213490_s_at	up	MAP2K2	1.2135351
214786_at	up	MAP3K1	1.2863754
205447_s_at	up	MAP3K12	1.2999891
221695_s_at	up	MAP3K2	1.487269
200835_s_at	up	MAP4	1.557063
204936_at	down	MAP4K2	-1.2586355
208351_s_at	up	MAPK1	1.2162853
237413_at	down	MAPK10	-1.2712121
210477_x_at	up	MAPK8	1.2603906
200712_s_at	up	MAPRE1	1.2076565
203841_x_at	up	MAPRE3	1.2011666
235385_at	up	MARCH1	1.267593
201737_s_at	down	MARCH6	-1.2144692
1558093_s_at	up	MATR3	1.5457156
1555611_s_at	up	MBD1	1.3159605
230298_at	down	MBLAC2	-1.2104106
1555594_a_at	up	MBNL1	1.2800454
210136_at	up	MBP	1.2946824
233231_at	up	MCCC2	1.2708365
1559309_at	up	MCFD2	1.4515995
223855_s_at	down	MCHR1	-1.2607422
215581_s_at	up	MCM3AP	1.3901755
232740_at	up	MCM3AP-AS	1.2018572
1555465_at	up	MCOLN2	1.2332513
217373_x_at	up	MDM2	1.2619742
242911_at	up	MED13L	1.4040172
219730_at	up	MED18	2.6807134
222438_at	down	MED4	-1.2093773
207968_s_at	up	MEF2C	1.7839372
238931_at	up	METT10D	1.2467695
1553688_at	up	METTL6	1.2007518
226346_at	down	MEX3A	-1.3013119
222567_s_at	up	MEX3C	1.6622223
231283_at	up	MGAT4A	1.2105018
1554305_at	up	MGC20647	1.2635732
221286_s_at	down	MGC29506	-1.2068632
200898_s_at	up	MGEA5	1.2410163
1569057_s_at	up	MIA3	1.2557929
205905_s_at	up	MICA /// MICB	1.2105322
209733_at	down	MID2	-1.2316021
212079_s_at	up	MLL	1.603879
217069_at	down	MLL4	-1.2281549
242082_at	up	MMAB	1.2325639
207118_s_at	up	MMP23A /// MMP23B	1.3434197
236749_at	up	MNT	1.2030722
201299_s_at	up	MOBKL1B	1.381707
218212_s_at	down	MOCS2	-1.2205924
206141_at	up	MOCS3	1.2339277
243683_at	up	MORF4L2	1.5049694
237158_s_at	up	MPHOSPH9	1.2062144
224173_s_at	up	MRPL30	1.6255686
213840_s_at	up	MRPS12	1.2399265
243821_at	up	MRPS31	1.375165
217418_x_at	up	MS4A1	1.2066582
224355_s_at	up	MS4A8B	1.2265275

224407_s_at	up	MST4	1.3950046
206800_at	up	MTHFR	1.2002832
226956_at	up	MTMR3	1.253336
244718_at	up	MTMR9L	1.3540175
218687_s_at	down	MUC13	-1.2597097
213432_at	up	MUC5B	1.2105894
1555448_at	up	MUDENG	1.4037958
215649_s_at	up	MVK	1.4291915
213906_at	down	MYBL1	-1.2098194
216188_at	down	MYCNOS	-1.2263637
229718_at	down	N4BP2L1	-1.2112918
220311_at	down	N6AMT1	-1.2459083
227651_at	down	NACC1	-1.2641237
244828_x_at	down	NAF1	-1.2337818
1555167_s_at	up	NAMPT	1.2202979
236188_s_at	down	NAP1L4	-1.2277783
235189_at	up	NARG2	1.2769282
214693_x_at	up	NBPF10	1.6959252
211685_s_at	up	NCALD	1.2398589
218662_s_at	down	NCAPG	-1.2104121
209734_at	up	NCKAP1L	1.3184203
221075_s_at	up	NCR2	1.3003516
232885_at	up	NCRNA00081	1.2070838
232093_at	up	NCRNA00085	1.2390313
222625_s_at	up	NDE1	1.2007399
217800_s_at	down	NDFIP1	-1.2383313
221082_s_at	up	NDRG3	1.3101386
1554010_at	down	NDST1	-1.2283449
235321_at	up	NDUFS1	1.5063697
214657_s_at	up	NEAT1	2.154953
221801_x_at	down	NEFL	-1.2041172
219396_s_at	up	NEIL1	1.4536289
213328_at	up	NEK1	1.2443074
214738_s_at	up	NEK9	1.3404329
213673_x_at	up	NENF	1.2067016
211094_s_at	up	NF1	1.3574911
238618_at	up	NF2	1.2066199
212808_at	up	NFATC2IP	1.2263535
207535_s_at	up	NFKB2	1.3780929
241889_at	up	NFKBID	1.2587924
207492_at	up	NGLY1	1.2905523
239314_at	up	NHLRC3	1.2315524
242352_at	up	NIPBL	1.5535007
230771_at	up	NKAIN4	1.2107782
215339_at	up	NKTR	1.3084674
1552553_a_at	down	NLRC4	-1.2205169
218036_x_at	up	NMD3	1.2811785
221566_s_at	up	NOL3	1.3251705
1554084_a_at	up	NOL9	1.2240145
203023_at	down	NOP16	-1.2076781
214101_s_at	down	NPEPPS	-1.2739216
215123_at	up	NPIPL3	2.2132967
207203_s_at	up	NR1I2	1.2121333
210841_s_at	up	NRP2	1.2761599
229649_at	up	NRXN3	1.9128551
224582_s_at	up	NUCKS1	1.3591244
209230_s_at	down	NUPR1	-1.3713002
209629_s_at	up	NXT2	2.0730102
228781_at	up	OBFC1	1.2584674
218903_s_at	down	OBFC2B	-1.2516003
1553051_s_at	down	ODF3	-1.2712837

1564805_a_at	down	OFCC1	-1.2007698
221090_s_at	down	OGFOD1	-1.3172287
226810_at	down	OGFRL1	-1.2090895
236121_at	down	OR51E2	-1.2606933
216536_at	down	OR7E19P	-1.2348139
228801_at	up	ORMDL1	1.2909124
235136_at	up	ORMDL3	1.4246253
1554414_a_at	up	OSGIN2	1.2542667
230170_at	down	OSM	-1.3815665
222825_at	down	OTUD6B	-1.239291
205432_at	up	OVGP1	1.2332786
214794_at	down	PA2G4	-1.2547312
1554691_a_at	up	PACSIN2	1.2960644
205564_at	up	PAGE4	1.2408985
200907_s_at	up	PALLD	1.213033
212720_at	up	PAPOLA	1.3968303
219236_at	up	PAQR6	1.3212606
214834_at	up	PAR5	1.5537438
235234_at	up	PATL1	1.3634104
210581_x_at	up	PATZ1	1.2862538
224152_s_at	up	PBRM1	1.2719182
213263_s_at	down	PCBP2	-1.2134027
203860_at	down	PCCA	-1.2217592
215277_at	down	PCDH1	-1.2461945
232382_s_at	up	PCMTD1	1.9381138
204870_s_at	up	PCSK2	1.2140542
202730_s_at	up	PDCD4	1.2305881
211171_s_at	up	PDE10A	1.3790535
224223_s_at	down	PDE11A	-1.2103171
222317_at	down	PDE3B	-1.2016855
224046_s_at	up	PDE7A	1.5684615
1552931_a_at	up	PDE8A	1.2675264
222860_s_at	up	PDGFD	1.4581184
221957_at	up	PDK3	1.340436
216804_s_at	up	PDLIM5	1.3626243
232861_at	up	PDP2	1.3593
221244_s_at	up	PDPK1	1.3142414
204742_s_at	down	PDS5B	-1.2251458
215481_s_at	up	PEX5	1.301508
226733_at	up	PFKFB2	1.3373339
231967_at	down	PHF20L1	-1.2343544
1554153_a_at	up	PHF21A	1.225892
217998_at	up	PHLDA1	1.2528713
228874_at	up	PHLDB3	1.2486728
215236_s_at	up	PICALM	1.4521581
1554508_at	up	PIK3AP1	1.3864944
1553694_a_at	up	PIK3C2A	1.4225479
206369_s_at	up	PIK3CG	1.3686428
212249_at	up	PIK3R1	1.3672674
1557719_at	up	PIKFYVE	1.309797
211205_x_at	up	PIP5K1A	1.2568467
232887_at	up	PIRT	1.2111629
1568949_at	up	PITPNC1	1.2801144
201927_s_at	up	PKP4	1.2325436
235110_at	up	PLA2G16	1.2503577
222795_s_at	up	PLCXD1	1.2912709
219566_at	up	PLEKHF1	1.2997558
236255_at	down	PLEKHG4B	-1.2072021
204939_s_at	down	PLN	-1.2218258
228104_at	down	PLXNA4	-1.224986
222406_s_at	up	PNRC2	1.2998168

1555383_a_at	up	POF1B	1.2651567
1553587_a_at	down	POLE4	-1.2411529
206654_s_at	up	POLR3G	1.4770805
228343_at	down	POU2F2	-1.2121457
1553639_a_at	up	PPARGC1B	1.203405
214146_s_at	down	PPBP	-1.2790129
202065_s_at	up	PPFIA1	1.3694404
231370_at	up	PPM1A	1.408786
244011_at	up	PPM1K	1.3044407
228222_at	down	PPP1CB	-1.3042175
201702_s_at	up	PPP1R10	1.3581536
1566303_s_at	down	PPP1R11	-1.2328879
211169_s_at	down	PPP1R3A	-1.2009858
225124_at	down	PPP1R9B	-1.2019509
202186_x_at	down	PPP2R5A	-1.2188685
211159_s_at	up	PPP2R5D	1.2175193
227630_at	down	PPP2R5E	-1.2342108
203529_at	down	PPP6C	-1.221561
235744_at	up	PPTC7	1.2888694
210670_at	up	PPY	1.451745
203456_at	up	PRAF2	1.2432952
207752_x_at	up	PRB1	1.2460561
214474_at	up	PRKAB2	1.4692214
227824_at	up	PRKCB	1.5953768
206270_at	down	PRKCG	-1.216325
241669_x_at	down	PRKD2	-1.3429691
206358_at	up	PRM1	1.2137188
220872_at	up	PRO2964	1.3229202
214203_s_at	up	PRODH	1.2663602
207401_at	up	PROX1	1.2211896
208879_x_at	up	PRPF6	1.2705022
230352_at	up	PRPS2	2.011175
205869_at	up	PRSS1	1.2168664
220051_at	up	PRSS21	1.2055304
200866_s_at	up	PSAP	1.2240603
215923_s_at	up	PSD4	1.4714817
207782_s_at	up	PSEN1	1.2608564
240091_at	down	PSMA8	-1.248967
212220_at	up	PSME4	1.3273816
212016_s_at	up	PTBP1	1.209326
1554614_a_at	up	PTBP2	1.4332104
211909_x_at	down	PTGER3	-1.3087537
204896_s_at	up	PTGER4	1.3020587
224950_at	up	PTGFRN	1.2019918
241453_at	up	PTK2	1.2458259
209465_x_at	down	PTN	-1.254488
200730_s_at	up	PTP4A1	1.6740797
209895_at	up	PTPN11	1.4620327
229362_at	up	PUS10	1.2219067
1558290_a_at	down	PVT1	-1.3563555
238046_x_at	down	PWWP2B	-1.2656078
219802_at	down	PYROXD1	-1.2127897
221270_s_at	up	QTRT1	1.3911604
208730_x_at	down	RAB2A	-1.2052106
1552846_s_at	down	RAB42	-1.2129914
206113_s_at	up	RAB5A	1.2406734
201048_x_at	up	RAB6A	1.3182312
222846_at	up	RAB8B	1.3052921
200607_s_at	up	RAD21	1.28602
201222_s_at	up	RAD23B	1.517674
236880_at	down	RAD52	-1.2401462

202482_x_at	down	RANBP1	-1.3199717
1558773_s_at	up	RANBP10	1.2014457
201711_x_at	up	RANBP2	1.4546378
210120_s_at	down	RANBP3	-1.2046494
229905_at	up	RAP1GDS1	1.2158082
214487_s_at	up	RAP2A /// RAP2B	1.3407
203096_s_at	up	RAPGEF2	1.2796888
204680_s_at	up	RAPGEF5	1.3172569
208530_s_at	up	RARB	1.2765503
208534_s_at	up	RASA4 /// RASA4P	1.2383194
1554999_at	up	RASGEF1B	1.4152662
244526_at	up	RASGRP3	1.3843083
49306_at	up	RASSF4	1.2583784
1554834_a_at	up	RASSF5	1.7692351
229147_at	down	RASSF6	-1.2008153
220680_at	down	RAVER2	-1.2555538
244872_at	up	RBBP4	1.2970577
205296_at	down	RBL1	-1.2286828
212332_at	up	RBL2	1.3096447
1555639_a_at	up	RBM14	1.2185898
212030_at	up	RBM25	1.2497025
227355_at	up	RBM26	1.2677643
243295_at	up	RBM27	1.2368121
229540_at	up	RBPJ	1.3988622
238421_at	up	RC3H2	1.3498273
215253_s_at	up	RCAN1	1.3321257
215747_s_at	up	RCC1	1.2344323
231290_at	up	RCCD1	1.2373477
1552378_s_at	up	RDH10	1.2376839
217042_at	up	RDH11	1.2581693
228815_s_at	down	REPIN1	-1.2646469
203696_s_at	down	RFC2	-1.200967
218430_s_at	up	RFX7	1.2080417
208492_at	up	RFXAP	1.2934612
232053_x_at	up	RHBDD2	1.3001236
1554586_a_at	up	RHOBTB2	1.2027346
225202_at	down	RHOBTB3	-1.2068317
1552734_at	up	RICTOR	1.290041
241820_at	up	RIF1	1.5660197
226164_x_at	up	RIMKLB	1.218177
227644_at	down	RIMS4	-1.2290297
202129_s_at	up	RIOK3	1.3536125
239843_at	up	RIT1	1.4179438
241343_at	up	RNASEH1	1.3129164
1568665_at	up	RNF103	1.240279
221430_s_at	up	RNF146	1.2100194
213038_at	down	RNF19B	-1.28376
244591_x_at	down	RNF207	-1.2251945
221194_s_at	up	RNFT1	1.2375506
211849_s_at	up	RNGTT	1.2514795
226975_at	up	RNPC3	1.2758292
235854_x_at	up	ROCK1	1.2846856
227849_at	down	RP9	-1.3087299
1555202_a_at	up	RPRD1A	1.2812835
1566720_at	up	RPS10P7	1.2844806
241241_at	up	RPS14	1.202032
218909_at	down	RPS6KC1	-1.2025446
201203_s_at	up	RRBP1	1.3286365
230952_at	down	RRN3P2	-1.2034118
214764_at	down	RRP15	-1.2728981
1554012_at	down	RSPO2	-1.2520705

216976_s_at	up	RYK	1.2388287
1553411_s_at	down	SALL3	-1.2206621
233001_at	up	SAMD10	1.3059096
1569599_at	up	SAMSN1	1.3768791
210790_s_at	up	SAR1A	1.2145623
234536_at	down	SARDH	-1.2055428
231895_at	down	SASS6	-1.2071501
213435_at	down	SATB2	-1.2463737
206667_s_at	up	SCAMP1	1.818395
201646_at	up	SCARB2	1.3623428
239454_at	down	SCARF2	-1.2787607
217600_at	up	SCUBE3	1.2554878
214166_at	up	SDHB	1.3223996
237598_at	down	SEC1	-1.3342972
224470_at	up	SEC22C	1.3926569
216392_s_at	up	SEC23IP	1.2186832
209889_at	up	SEC31B	1.2235045
222385_x_at	up	SEC61A1	1.2277613
208942_s_at	down	SEC62	-1.2364924
230265_at	up	SEL1L	2.1871579
223610_at	up	SEMA5B	1.2528108
223449_at	up	SEMA6A	1.2717769
1552812_a_at	up	SENP1	1.4154449
1569974_x_at	down	SEPT7P2	-1.2163311
212268_at	up	SERPINB1	1.2601981
210172_at	up	SF1	1.6793821
202036_s_at	down	SFRP1	-1.2926308
202773_s_at	up	SFSWAP	1.3958496
214354_x_at	up	SFTPB	1.3084375
220008_at	up	SGK269	1.2009856
235425_at	down	SGOL2	-1.2952648
227923_at	up	SHANK3	1.2362424
219493_at	down	SHCBP1	-1.3287972
1552806_a_at	up	SIGLEC10 /// SIGLEC12	1.2042744
1552911_at	up	SIGLEC11	1.2368029
208078_s_at	down	SIK1	-1.2145748
238006_at	down	SIN3A	-1.2486405
242129_at	up	SIN3B	1.2242844
228347_at	down	SIX1	-1.205419
217640_x_at	down	SKA1	-1.2202777
203626_s_at	up	SKP2	1.3183829
224854_s_at	up	SLAIN2	1.4008476
234306_s_at	up	SLAMF7	1.3527923
220740_s_at	up	SLC12A6	1.2041407
225043_at	up	SLC15A4	1.3279849
1557918_s_at	up	SLC16A1	1.3504031
202856_s_at	up	SLC16A3	1.2586629
206599_at	down	SLC16A5	-1.2317543
206396_at	up	SLC1A1	1.2255124
237741_at	up	SLC25A36	1.3550351
218136_s_at	up	SLC25A37	1.3462328
231187_at	down	SLC28A1	-1.2183646
204429_s_at	up	SLC2A5	1.3449061
239596_at	up	SLC30A7	1.2320589
207439_s_at	up	SLC35A2	1.3793849
238347_at	down	SLC38A10	-1.2498363
220413_at	up	SLC39A2	1.2097262
1556551_s_at	up	SLC39A6	1.324786
216504_s_at	up	SLC39A8	1.4625964
243894_at	up	SLC41A2	1.2238272
210692_s_at	up	SLC43A3	1.2657751

213167_s_at	up	SLC5A3	1.3581944
214860_at	up	SLC9A7	1.3646177
1553315_at	up	SLFNL1	1.3791132
224149_x_at	up	SLMAP	1.4679229
222441_x_at	up	SLMO2	1.2856749
213565_s_at	down	SMAD6	-1.2128699
212257_s_at	up	SMARCA2	1.5270525
217656_at	up	SMARCA4	1.2888131
1558747_at	up	SMCHD1	1.418564
238434_at	up	SMCR8	1.2463514
201793_x_at	up	SMG7	1.3407514
205309_at	up	SMPDL3B	1.4094319
209427_at	down	SMTN	-1.2547399
209131_s_at	up	SNAP23	1.5971612
1566403_at	up	SNORA68	1.2123123
226438_at	down	SNTB1	-1.2145936
220140_s_at	up	SNX11	1.2068441
229045_at	up	SNX20	1.22107
201085_s_at	up	SON	1.4134407
217576_x_at	up	SOS2	1.2219704
204432_at	down	SOX12	-1.2580765
1553685_s_at	up	SP1	2.0792925
239743_at	down	SP8	-1.2185072
219888_at	up	SPAG4	1.2389477
229075_at	down	SPATA5	-1.20239
214403_x_at	down	SPDEF	-1.288168
216119_s_at	down	SPEF1	-1.2985827
201996_s_at	up	SPEN	1.3673474
219257_s_at	up	SPHK1	1.207213
244439_at	up	SPRED1	1.2163281
208539_x_at	down	SPRR2B	-1.261287
232082_x_at	down	SPRR3	-1.3679508
224144_at	down	SPTBN4	-1.2943119
202278_s_at	up	SPTLC1	1.2286109
1557352_at	up	SQLE	1.5077261
244804_at	up	SQSTM1	1.220337
1553581_s_at	down	SREK1IP1	-1.2559975
1568957_x_at	up	SRGAP2P1	1.2570063
202199_s_at	up	SRPK1	1.2542448
216639_at	down	SRPX2	-1.2295233
208610_s_at	up	SRRM2	1.2696333
201742_x_at	up	SRSF1	1.3683664
213742_at	down	SRSF11	-1.2527901
212266_s_at	up	SRSF5	1.2475252
223635_s_at	down	SSBP3	-1.2413251
200890_s_at	up	SSR1	1.2888123
217790_s_at	up	SSR3	1.2095879
214555_at	down	SSTR5	-1.3298289
214971_s_at	up	ST6GAL1	1.4594728
207524_at	up	ST7	1.2825435
209622_at	up	STK16	1.2114447
215505_s_at	up	STRN3	1.60572
203768_s_at	up	STS	1.2445357
226655_at	up	STX17	1.3695139
230691_at	up	STX1B	1.2003728
235180_at	up	STYX	1.267792
237784_at	up	SUB1	1.5253067
207601_at	up	SULT1B1	1.2181348
240200_at	up	SULT1C2	1.2146759
233827_s_at	up	SUPT16H	1.4232175
216271_x_at	up	SYDE1	1.3616

242774_at	up	SYNE2	2.3912773
215860_at	down	SYT12	-1.2480085
205966_at	up	TAF13	1.3606119
202840_at	up	TAF15	1.355117
1553528_a_at	down	TAF5	-1.2176661
221618_s_at	up	TAF9B	1.7934484
242388_x_at	up	TAGAP	1.211516
1555565_s_at	up	TAPBP	1.3154305
1564883_a_at	down	TAS1R1	-1.213903
234042_at	up	TAS2R45	1.2266921
213913_s_at	down	TBC1D30	-1.2710199
222634_s_at	up	TBL1XR1	1.319699
1553132_a_at	down	TC2N	-1.220804
226388_at	up	TCEA3	1.2056544
202371_at	down	TCEAL4	-1.2451591
1553635_s_at	down	TCTEX1D1	-1.261144
202719_s_at	up	TES	1.209044
221035_s_at	up	TEX14	1.2990773
205688_at	down	TFAP4	-1.2406025
215863_at	down	TFR2	-1.3023049
233983_at	up	TGM6	1.2137678
203833_s_at	up	TGOLN2	1.4449682
235653_s_at	up	THAP6	1.2576196
209419_at	up	THOC5	1.5055614
222439_s_at	up	THRAP3	1.4155089
229758_at	down	TIGD5	-1.2184504
235765_at	up	TLE4	1.3426694
220639_at	down	TM4SF20	-1.239756
204426_at	up	TMED2	1.3311411
224496_s_at	up	TMEM107	1.4161645
1564067_x_at	down	TMEM151B	-1.3088908
213272_s_at	up	TMEM159	1.4152834
228671_at	down	TMEM201	-1.255594
226647_at	up	TMEM25	1.2335995
232591_s_at	up	TMEM30A	1.2167075
222736_s_at	up	TMEM38B	1.4124749
219462_at	up	TMEM53	1.2220973
226083_at	down	TMEM70	-1.3651098
242103_at	up	TMEM86A	1.2434255
225412_at	up	TMEM87B	1.2428396
232941_at	down	TMPRSS6	-1.3270667
208097_s_at	up	TMX1	1.6823621
202510_s_at	up	TNFAIP2	1.2287173
222562_s_at	up	TNKS2	1.684256
210585_s_at	up	TNPO2	1.3838719
234734_s_at	down	TNRC6A	-1.2421542
228998_at	up	TNRC6B	1.2436055
208900_s_at	up	TOP1	1.2583352
216100_s_at	up	TOR1AIP1	1.4923571
201683_x_at	up	TOX4	1.3020196
1569098_s_at	up	TP53BP1	1.2252831
211194_s_at	up	TP63	1.5798955
231978_at	up	TPCN2	1.2877588
238065_at	down	TPM3	-1.3146789
218876_at	down	TPPP3	-1.2144017
215220_s_at	up	TPR	1.789101
213575_at	up	TRA2A	1.4068787
201399_s_at	up	TRAM1	1.4255743
234440_at	down	TRDV3	-1.2984595
219937_at	down	TRHDE	-1.2900738
202479_s_at	up	TRIB2	1.2289041

210995_s_at	up	TRIM23	1.2798948
225868_at	down	TRIM47	-1.2430952
1568594_s_at	up	TRIM52	1.208695
235476_at	up	TRIM59	1.3533504
208178_x_at	up	TRIO	1.2142777
1568596_a_at	down	TROAP	-1.2470404
210438_x_at	down	TROVE2	-1.2332542
208589_at	down	TRPC7	-1.2523592
234351_x_at	up	TRPS1	1.3097113
222855_s_at	up	TRPV2	1.2624544
214908_s_at	up	TRRAP	1.3985238
235447_at	up	TRUB1	1.2014401
208763_s_at	up	TSC22D3	1.3510768
227610_at	up	TSPAN11	1.2390826
242595_at	down	TSSK4	-1.2497983
1569472_s_at	up	TTC3	2.0169203
208099_x_at	down	TTLL5	-1.22137
228883_at	up	TUB	1.2046323
215739_s_at	up	TUBGCP3	1.230523
214007_s_at	up	TWF1	1.4828343
219969_at	up	TXLNG	1.2185957
201008_s_at	up	TXNIP	1.474721
202954_at	down	UBE2C	-1.2116129
209041_s_at	up	UBE2G2	1.2502512
217799_x_at	up	UBE2H	1.2914091
225179_at	up	UBE2K	1.3215816
212404_s_at	up	UBE3B	1.2354816
202316_x_at	up	UBE4B	1.431709
243916_x_at	down	UBLCP1	-1.3228263
209088_s_at	up	UBN1	1.2053555
212008_at	up	UBXN4	1.3866497
217100_s_at	up	UBXN7	1.2896739
221765_at	up	UGCG	1.588019
1552656_s_at	up	UHMK1	1.444792
202707_at	up	UMPS	1.2337593
225869_s_at	up	UNC93B1	1.2116233
208947_s_at	up	UPF1	1.3120805
202412_s_at	up	USP1	1.2643702
209136_s_at	up	USP10	1.2028841
1552678_a_at	up	USP28	1.2405721
212065_s_at	up	USP34	1.4104941
224979_s_at	down	USP36	-1.2450577
226729_at	down	USP37	-1.2221959
244133_at	up	USP49	1.2217152
242242_at	up	USP6	1.2613249
238561_s_at	up	UTP23	1.3668115
218169_at	down	VAC14	-1.2132423
226029_at	down	VANGL2	-1.2071311
202549_at	up	VAPB	1.2119246
208844_at	down	VDAC3	-1.3201733
235237_at	up	VMA21	1.295458
1558549_s_at	down	VNN1	-1.2886419
231585_at	down	VPS13A	-1.2454926
1553852_at	down	VPS13B	-1.2053165
232386_at	up	VPS13C	1.3182617
244400_at	down	VPS33A	-1.2361475
222387_s_at	up	VPS35	1.5461305
219679_s_at	up	WAC	1.468996
224563_at	up	WASF2	1.3752091
205809_s_at	up	WASL	1.3241796
203597_s_at	down	WBP4	-1.2457443

1554410_a_at	up	WBSCR16	1.3194683
228326_at	up	WDR27	1.2001189
209216_at	down	WDR45	-1.2053094
228295_at	up	WDR59	1.3030602
1570033_at	down	WIPI2	-1.2018325
211993_at	up	WNK1	1.3538823
229547_s_at	down	WNK2	-1.2346348
201295_s_at	up	WSB1	1.5458132
1560274_at	up	WTAP	1.4658818
218110_at	down	XAB2	-1.2017821
1555785_a_at	up	XRN1	1.2922058
231550_at	up	XYLT2	1.2105894
200641_s_at	up	YWHAZ	1.275037
243648_at	up	ZBED6	1.7465087
226284_at	down	ZBTB2	-1.2484276
1554973_a_at	up	ZBTB26	1.3215235
223953_s_at	up	ZBTB37	1.2880547
230754_at	down	ZBTB38	-1.2643255
229691_at	down	ZBTB42	-1.273353
213303_x_at	down	ZBTB7A	-1.2313094
205787_x_at	up	ZC3H11A	1.2317656
226897_s_at	up	ZC3H7A	1.4115872
1556967_at	up	ZDHHC14	1.5948302
212758_s_at	up	ZEB1	1.2435546
234455_at	down	ZFP1	-1.2003193
224631_at	up	ZFP91	1.3623356
203730_s_at	up	ZKSCAN5	1.2241715
1555609_a_at	up	ZMAT3	1.728943
232508_at	down	ZMIZ1	-1.202388
210282_at	up	ZMYM2	1.4444937
243814_at	up	ZMYND8	1.218426
1567032_s_at	up	ZNF160	1.2833675
1558184_s_at	down	ZNF17	-1.2054374
207164_s_at	up	ZNF238	1.3226553
228185_at	up	ZNF25	1.20233
241720_at	up	ZNF326	1.2385306
220117_at	down	ZNF385D	-1.2603899
232117_at	down	ZNF471	-1.2525529
228138_at	up	ZNF498	1.2042183
1558995_at	up	ZNF547	1.3093116
1556187_at	up	ZNF555	1.3690343
228305_at	up	ZNF565	1.2549423
212620_at	up	ZNF609	1.2321737
227045_at	down	ZNF614	-1.3506169
1554249_a_at	up	ZNF638	1.2135195
235179_at	up	ZNF641	1.2808677
1554726_at	up	ZNF655	1.573
222760_at	down	ZNF703	-1.2891688
235079_at	down	ZNF704	-1.2210963
238606_at	down	ZNF747	-1.2295533
239441_at	down	ZNF780A	-1.2400992
227101_at	up	ZNF800	1.5563715
228130_at	up	ZSCAN30	1.386811
217592_at	up	ZSWIM1	1.2098714
227918_s_at	up	ZYG11B	1.322702

Table S3: GSEA "Aggressive multiple myeloma" signature

PROBE	GENE SYMBOL	GENE_TITLE	RANK IN GENE LIST	RANK METRIC SCORE	RUNNING ES	CORE ENRICHMENT
KIF14	*KIF14*	kinesin family member 14	3	0.751	0.0594	Yes
CCNB1	*CCNB1*	cyclin B1	5	0.727	0.1171	Yes
DEPDC1B	*DEPDC1B*	DEP domain containing 1B	20	0.652	0.1681	Yes
ASPM	*ASPM*	asp (abnormal spindle) homolog, microcephaly associated (Drosophila)	97	0.544	0.2075	Yes
UBE2C	*UBE2C*	ubiquitin-conjugating enzyme E2C	119	0.521	0.2478	Yes
NEK2	*NEK2*	NIMA (never in mitosis gene a)-related kinase 2	328	0.432	0.2720	Yes
C18ORF24	*C18ORF24*	chromosome 18 open reading frame 24	340	0.430	0.3055	Yes
FOXM1	*FOXM1*	forkhead box M1	501	0.394	0.3290	Yes
SLC19A1	*SLC19A1*	solute carrier family 19 (folate transporter), member 1	547	0.386	0.3575	Yes
MAGEA3	*MAGEA3*	melanoma antigen family A, 3	675	0.370	0.3806	Yes
SPBC25	*SPBC25*	spindle pole body component 25 homolog (S. cerevisiae)	762	0.359	0.4049	Yes
AURKA	*AURKA*	aurora kinase A	794	0.356	0.4317	Yes
CDCA1	*CDCA1*	cell division cycle associated 1	861	0.348	0.4561	Yes
BIRC5	*BIRC5*	baculoviral IAP repeat-containing 5 (survivin)	1039	0.330	0.4737	Yes
TTK	*TTK*	TTK protein kinase	1061	0.328	0.4987	Yes
TOP2A	*TOP2A*	topoisomerase (DNA) II alpha 170kDa	1262	0.312	0.5137	Yes
DLG7	*DLG7*	discs, large homolog 7 (Drosophila)	1304	0.309	0.5362	Yes
UBE2T	*UBE2T*	ubiquitin-conjugating enzyme E2T (putative)	1437	0.300	0.5536	Yes
LOC652689/FAM72A	*not available*		1813	0.276	0.5573	Yes
ZWILCH	*ZWILCH*	Zwilch, kinetochore associated, homolog (Drosophila)	2068	0.261	0.5656	Yes
GAGE1/GAGE2	*not available*		2088	0.260	0.5853	Yes
MAGEA1	*MAGEA1*	melanoma antigen family A, 1 (directs expression of antigen MZ2-E)	2155	0.257	0.6025	Yes
BUB1B	*BUB1B*	BUB1 budding uninhibited by benzimidazoles 1 homolog beta (yeast)	2341	0.249	0.6132	Yes
SPBC24	*SPBC24*	spindle pole body component 24 homolog (S. cerevisiae)	2596	0.237	0.6196	Yes
MAGEA6	*MAGEA6*	melanoma antigen family A, 6	3708	0.194	0.5810	Yes
E2F8	*E2F8*	E2F transcription factor 8	3781	0.191	0.5926	Yes
DCC1	*not available*		3913	0.187	0.6011	Yes
MCM10	*MCM10*	MCM10 minichromosome maintenance deficient 10 (S. cerevisiae)	3995	0.185	0.6119	Yes
CCDC34	*CCDC34*	coiled-coil domain containing 34	4039	0.184	0.6244	Yes
TRIP13	*TRIP13*	thyroid hormone receptor interactor 13	4150	0.181	0.6333	Yes
RFC4	*RFC4*	replication factor C (activator 1) 4, 37kDa	4317	0.176	0.6392	Yes
PRC1	*PRC1*	protein regulator of cytokinesis 1	4353	0.175	0.6513	Yes
TYMS	*TYMS*	thymidylate synthetase	4523	0.170	0.6566	Yes
TPX2	*TPX2*	TPX2, microtubule-associated, homolog (Xenopus laevis)	5070	0.154	0.6422	Yes
KIAA1794	*KIAA1794*	KIAA1794	5203	0.150	0.6477	Yes
CDC2	*CDC2*	cell division cycle 2, G1 to S and G2 to M	5245	0.149	0.6575	Yes

Table S4: GSEA "CD40 dependant B cell" signature

PROBE	GENE SYMBOL	GENE_TITLE	RANK IN GENE LIST	RANK METRIC SCORE	RUNNING ES	CORE ENRICHMENT
NFKB2	*NFKB2*	nuclear factor of kappa light polypeptide gene enhancer in B-cells 2 (p49/p100)	20573	-0.743	0.0016	Yes
NCKAP1L	*NCKAP1L*	NCK-associated protein 1-like	20563	-0.706	-0.0373	Yes
TNFAIP2	*TNFAIP2*	tumor necrosis factor, alpha-induced protein 2	20470	-0.517	-0.0702	Yes
CD40	*CD40*	CD40 molecule, TNF receptor superfamily member 5	20467	-0.515	-0.0975	Yes
CCR7	*CCR7*	chemokine (C-C motif) receptor 7	20438	-0.495	-0.1233	Yes
CD83	*CD83*	CD83 molecule	20429	-0.490	-0.1491	Yes
DUSP2	*DUSP2*	dual specificity phosphatase 2	20415	-0.481	-0.1744	Yes
HTR3A	*HTR3A*	5-hydroxytryptamine (serotonin) receptor 3A	20267	-0.423	-0.1927	Yes
SLAMF1	*SLAMF1*	signaling lymphocytic activation molecule family member 1	20177	-0.395	-0.2108	Yes
HLA-DQB1	*HLA-DQB1*	major histocompatibility complex, class II, DQ beta 1	19998	-0.363	-0.2230	Yes
TNFAIP8	*TNFAIP8*	tumor necrosis factor, alpha-induced protein 8	19949	-0.355	-0.2398	Yes
TRIP10	*TRIP10*	thyroid hormone receptor interactor 10	19874	-0.343	-0.2550	Yes
BCL2	*BCL2*	B-cell CLL/lymphoma 2	19667	-0.316	-0.2632	Yes
PHACTR1	*PHACTR1*	phosphatase and actin regulator 1	19586	-0.307	-0.2760	Yes
FAIM3	*FAIM3*	Fas apoptotic inhibitory molecule 3	19526	-0.298	-0.2893	Yes
MAP3K8	*MAP3K8*	mitogen-activated protein kinase kinase kinase 8	19506	-0.296	-0.3042	Yes
STAT5A	*STAT5A*	signal transducer and activator of transcription 5A	19496	-0.295	-0.3194	Yes
NFKBIA	*NFKBIA*	nuclear factor of kappa light polypeptide gene enhancer in B-cells inhibitor, alpha	19468	-0.292	-0.3337	Yes
DDIT4	*DDIT4*	DNA-damage-inducible transcript 4	19443	-0.290	-0.3480	Yes
CFLAR	*CFLAR*	CASP8 and FADD-like apoptosis regulator	19434	-0.289	-0.3630	Yes
CR2	*CR2*	complement component (3d/Epstein Barr virus) receptor 2	19401	-0.286	-0.3768	Yes
CCL4	*CCL4*	chemokine (C-C motif) ligand 4	19304	-0.278	-0.3872	Yes
EBI2	*EBI2*	Epstein-Barr virus induced gene 2 (lymphocyte-specific G protein-coupled receptor)	19233	-0.270	-0.3985	Yes
UNC119	*UNC119*	unc-119 homolog (C. elegans)	19195	-0.267	-0.4109	Yes
TNFAIP3	*TNFAIP3*	tumor necrosis factor, alpha-induced protein 3	18909	-0.244	-0.4112	Yes
LRRC32	*LRRC32*	leucine rich repeat containing 32	18894	-0.243	-0.4234	Yes
ZFP36L1	*ZFP36L1*	zinc finger protein 36, C3H type-like 1	18764	-0.235	-0.4300	Yes
ICAM1	*ICAM1*	intercellular adhesion molecule 1 (CD54), human rhinovirus receptor	18745	-0.233	-0.4416	Yes
KRT7	*KRT7*	keratin 7	18666	-0.228	-0.4501	Yes
NEF3	*NEF3*	neurofilament 3 (150kDa medium)	18243	-0.204	-0.4416	Yes
MEF2C	*MEF2C*	MADS box transcription enhancer factor 2, polypeptide C (myocyte enhancer factor 2C)	18204	-0.201	-0.4505	Yes
TRAF1	*TRAF1*	TNF receptor-associated factor 1	17831	-0.182	-0.4430	Yes
PLEK	*PLEK*	pleckstrin	17794	-0.180	-0.4509	Yes
LYN	*LYN*	v-yes-1 Yamaguchi sarcoma viral related oncogene homolog	17576	-0.170	-0.4498	Yes
NFKBIE	*NFKBIE*	nuclear factor of kappa light polypeptide gene enhancer in B-cells inhibitor, epsilon	17522	-0.168	-0.4563	Yes
RAC2	*RAC2*	ras-related C3 botulinum toxin substrate 2 (rho family, small GTP binding protein Rac2)	17513	-0.168	-0.4648	Yes
SYNGR2	*SYNGR2*	synaptogyrin 2	17446	-0.165	-0.4704	Yes
TNF	*TNF*	tumor necrosis factor (TNF superfamily, member 2)	17428	-0.165	-0.4783	Yes
HLA-DRB1 ///	*HLA-DRB1*	Major histocompatibility complex, class II, DR beta 1	17296	-0.158	-0.4807	Yes
BLR1	*BLR1*	Burkitt lymphoma receptor 1, GTP binding protein (chemokine (C-X-C motif) receptor 5)	17189	-0.154	-0.4839	Yes
ARID5B	*ARID5B*	AT rich interactive domain 5B (MRF1-like)	17158	-0.153	-0.4905	Yes
GADD45B	*GADD45B*	growth arrest and DNA-damage-inducible, beta	16998	-0.146	-0.4908	Yes
PIK3CD	*PIK3CD*	phosphoinositide-3-kinase, catalytic, delta polypeptide	16885	-0.141	-0.4931	Yes
HLA-F	*HLA-F*	major histocompatibility complex, class I, F	16880	-0.141	-0.5004	Yes
TMC6	*TMC6*	transmembrane channel-like 6	16866	-0.141	-0.5072	Yes

Table S5: Oligonucleotides used for this study

sh cloning

Duplex name	Oligo Name	Sequence 5' to 3'
shCY1 = shCYCLON	Cyclonsh2F	CGTGTACCGGTCCGTCCAAGAAGTTGAATAAAGCTCGAGCTTTATTCAACTTCTTGGACGTTTTTGAATTCCGTCGT
	Cyclonsh2R	ACGACGGAATTCAAAAACGTCCAAGAAGTTGAATAAAGCTCGAGCTTTATTCAACTTCTTGGACGGACCGGTACACG
shCY2	Cyclonsh3F	CGTGTACCGGTCCCCAGATGCTTCAGGACAAGCCTCGAGGCTTGTCCTGAAGCATCTGGGTTTTTGAATTCCGTCGT
	Cyclonsh3R	ACGACGGAATTCAAAAACCCAGATGCTTCAGGACAAGCCTCGAGGCTTGTCCTGAAGCATCTGGGGACCGGTACACG
shCtrl	CtrlshF	CGTGTACCGGTCCCTAAGGTTAAGTCGCCCTCGCTCGAGCGAGGGCGACTTAACCTTAGGTTTTTGAATTCCGTCGT
	CtrlshR	ACGACGGAATTCAAAAACCTAAGGTTAAGTCGCCCTCGCTCGAGCGAGGGCGACTTAACCTTAGGGACCGGTACACG

q-PCR

Oligo Name	Sequence 5' to 3'
CyclonF	AGCGAAAAGGTTCTTCATCC
CyclonR	TTCCTCTCCTGTCGTTCCTT
